# Montessori education's impact on academic and nonacademic outcomes: A systematic review

**DOI:** 10.1002/cl2.1330

**Published:** 2023-08-07

**Authors:** Justus J. Randolph, Anaya Bryson, Lakshmi Menon, David K. Henderson, Austin Kureethara Manuel, Stephen Michaels, debra leigh walls rosenstein, Warren McPherson, Rebecca O'Grady, Angeline S. Lillard

**Affiliations:** ^1^ Georgia Baptist College of Nursing Mercer University Atlanta Georgia USA; ^2^ Tift College of Education Mercer University Atlanta Georgia USA; ^3^ Fairfax County Public Schools Falls Church Virginia USA; ^4^ Gordon T. & Ellen West College of Education Midwestern State University Texas USA; ^5^ University Libraries University of North Georgia Watkinsville Georgia USA; ^6^ University Libraries Mercer University Atlanta Georgia USA; ^7^ Athens Montessori School Athens Georgia USA; ^8^ Department of Psychology University of Virginia Charlottesville Virginia USA

## Abstract

**Background:**

Montessori education is the oldest and most widely implemented alternative education in the world, yet its effectiveness has not been clearly established.

**Objectives:**

The primary objective of this review was to examine the effectiveness of Montessori education in improving academic and nonacademic outcomes compared to traditional education. The secondary objectives were to determine the degree to which grade level, Montessori setting (public Montessori vs. private Montessori), random assignment, treatment duration, and length of follow‐up measurements moderate the magnitude of Montessori effects.

**Search Methods:**

We searched for relevant studies in 19 academic databases, in a variety of sources known to publish gray literature, in Montessori‐related journals, and in the references of studies retrieved through these searches. Our search included studies published during or before February 2020. The initial search was performed in March 2014 with a follow‐up search in February 2020.

**Selection Criteria:**

We included articles that compared Montessori education to traditional education, contributed at least one effect size to an academic or nonacademic outcome, provided sufficient data to compute an effect size and its variance, and showed sufficient evidence of baseline equivalency–through random assignment or statistical adjustment–of Montessori and traditional education groups.

**Data Collection and Analysis:**

To synthesize the data, we used a cluster‐robust variance estimation procedure, which takes into account statistical dependencies in the data. Otherwise, we used standard methodological procedures as specified in the Campbell Collaboration reporting and conduct standards.

**Main Results:**

Initial searches yielded 2012 articles, of which 173 were considered in detail to determine whether they met inclusion/exclusion criteria. Of these, 141 were excluded and 32 were included. These 32 studies yielded 204 effect sizes (113 academic and 91 nonacademic) across 132,249 data points. In the 32 studies that met minimum standards for inclusion, including evidence of baseline equivalence, there was evidence that Montessori education outperformed traditional education on a wide variety of academic and nonacademic outcomes. For academic outcomes, Hedges' *g* effect sizes, where positive values favor Montessori, ranged from 0.26 for general academic ability (with high quality evidence) to 0.06 for social studies. The quality of evidence for language (*g* = 0.17) and mathematics (*g* = 0.22) was also high. The effect size for a composite of all academic outcomes was 0.24. Science was the only academic outcome that was deemed to have low quality of evidence according to the GRADE approach. Effect sizes for nonacademic outcomes ranged from 0.41 for students' inner experience of school to 0.23 for social skills. Both of these outcomes were deemed as having low quality of evidence. Executive function (*g* = 0.36) and creativity (*g* = 0.26) had moderate quality of evidence. The effect size for a composite of all nonacademic outcomes was 0.33. Moderator analyses of the composite academic and nonacademic outcomes showed that Montessori education resulted in larger effect sizes for randomized studies compared to nonrandomized studies, for preschool and elementary settings compared to middle school or high school settings, and for private Montessori compared to public Montessori. Moderator analyses for treatment duration and duration from intervention to follow‐up data collection were inconclusive. There was some evidence for a lack of small sample‐size studies in favor of traditional education, which could be an indicator of publication bias. However, a sensitivity analysis indicated that the findings in favor of Montessori education were nonetheless robust.

**Authors' Conclusions:**

Montessori education has a meaningful and positive impact on child outcomes, both academic and nonacademic, relative to outcomes seen when using traditional educational methods.

## 1 PLAIN LANGUAGE SUMMARY

### 1.1 Montessori education significantly impacts academic and nonacademic outcomes

Relative to traditional education, Montessori education has modest but meaningful positive effects on children's academic and nonacademic (executive function, creativity and social‐emotional) outcomes. This is indicated by a meta‐analysis of 32 studies in which it was possible to compare traditional business‐as‐usual education to Montessori education.

#### 1.1.1 What is this review about?

How best to educate children is an issue of enduring concern, and Montessori is the most common alternative to the conventional education system. Montessori includes a full system of lessons and hands‐on materials for children from birth to 18 years, presented individually, and embedded in a philosophical framework regarding children's development and its optimal conditions.

The term Montessori is not trademarked, and, therefore, its implementation can vary. We studied the range of variations included in the literature, which likely reflects the range of implementations encountered in the world. We also compared Montessori with a range of control conditions described in the literature as traditional (sometimes referred to as conventional, or business‐as‐usual), reflecting the implementation of traditional education in the real world.

**What are the main findings of the review?**

Using only studies with evidence of baseline equivalence, this review found that Montessori education had a significant positive impact on academic and nonacademic outcomes. Studies with random assignment, elementary school age level, and private Montessori schools had larger effects.

#### 1.1.2 What studies are included?

From a search yielding over 2,000 studies, the review evaluated 32 of the most rigorous Montessori studies, with publication dates ranging from 1967 to 2020.

Study participants were spread across age levels: preschool, elementary school and middle and high school.

The studies took place in eight countries: the USA (18 studies), Turkey (four studies), Switzerland (three studies) and one each in England, France, Malaysia, Oman, Iran, The Philippines, and Thailand.

#### 1.1.3 How effective is Montessori education?

On academic outcomes, Montessori students performed about 1/4 of a standard deviation better than students in traditional education. The magnitude of these effects could be considered small when compared to findings obtained in tightly‐controlled laboratory studies, but they could be considered to be medium‐large to large when compared to studies in real‐world school contexts involving standardized tests.

Most (28) of the included studies were conducted in schools implementing Montessori as a full program; the remaining Four studies were short‐term add‐ons to otherwise traditional school curricula.

The effect sizes for academic outcomes are similar to those obtained in other studies that compared “No Excuses” charter schools to business‐as‐usual urban schools.

The magnitude of Montessori education's nonacademic effects was slightly stronger than its effects on academic outcomes. Montessori students performed about 1/3 of a standard deviation higher than students in traditional education on nonacademic outcomes, including self‐regulation (executive function), well‐being at school, social skills, and creativity.

The magnitude of Montessori education's effects was greater for randomized than non‐randomized study designs, greater for preschool and elementary school than for middle and high school, and greater for private Montessori compared to public Montessori settings.

#### 1.1.4 What do the findings of this review mean?

Across a wide range of implementations (likely reflecting the range of Montessori implementations in the real world) and in studies of moderate to high quality, Montessori education has a nontrivial impact on children's academic and nonacademic outcomes.

#### 1.1.5 How up to date is this review?

The review authors searched for studies published through February 2020.

## 2 BACKGROUND

Montessori education is the most widespread alternative education, yet its effectiveness is uncertain. Here we describe the problem addressed by the review, then discuss the Montessori intervention and why it might be effective. Finally, we discuss why it is important to do this review.

### 2.1 Description of the condition

Dissatisfaction with education has been longstanding, with half or fewer American parents satisfied with K‐12 education in the United States over the last 30 years. The United States as a whole performs poorly on international tests like the PISA, and national tests like the NAEP show little progress over the years and a severe drop in performance with the COVID‐19 pandemic. Achievement is particularly concerning among lower‐income children and children of color (Duncan, 2014). A prominent education scholar recently stated, “Preparing all students to meet higher academic standards will require instruction that is different and much better than the instruction that most students receive today” (Duncan, 2014, p. 141). There is also concern about nonacademic outcomes of children, including how to increase their executive function and social‐emotional skills (Ahmed, 2021; Jones, 2015). Given that education, as it is usually implemented, has not yielded sufficiently positive outcomes to lead to widespread satisfaction, a question arises as to how alternative forms of education fare in terms of delivered outcomes. One such alternative is Montessori education.

Although Montessori education is the oldest continuously implemented, as well as the most widely implemented alternative education in the world (Lillard, 2019), currently used in over 550 public and 3000 private schools in the United States alone, evidence of its outcomes has not been rigorously compiled. Schooling techniques should be based on evidence of what works; this is why the Department of Education established the What Works Clearinghouse. And yet that Clearinghouse's current (yet outdated) entry for Montessori Method states that as of December 2005, it is unable to draw evidence‐based conclusions about the effectiveness of Montessori. Recent reviews also note that its effectiveness has not been clearly established (Ackerman, 2019; Marshall, 2017). This is a problem, given that parents and school districts want to and should make schooling decisions based on evidence.

The main objective of this review is to determine if Montessori education impacts academic and/or nonacademic outcomes of children, and, thus, whether it should be further explored as a possible type of school reform to address the shortcomings of traditional education. Secondary objectives are to determine if its impacts vary at different ages, in public versus private school settings, with the duration of a child's participation in Montessori, and with the length of time since a Montessori intervention (either fade‐out effects or, by contrast, sleeper effects where the impact strengthens with time). The knowledge gained from this analysis could be important to education policy at national, state, district, and school levels, as well as for parents making individual decisions about their children's education.

### 2.2 Description of the intervention

Maria Montessori and her collaborators developed a system of education based on observations first of atypically‐developing children, then of lower‐income children, and finally a variety of children in widely disparate cultures (from India to Europe to America) (Montessori, 2017). Montessori is currently available in over 150 countries.

Through observation, Montessori developed a distinct philosophy of education that was rooted in her medical training (Trabalzini, 2011). She viewed children as biological organisms driven toward their ultimate adult state by internal forces (Montessori, 2012; Montessori, 2017). When nothing perturbs this development (akin to poor nutrition or other environmental disturbances), she believed children would make optimal choices to propel their own development forward. Thus, in Montessori programs children choose which materials to use at any given time; teacher guidance is given only as needed (i.e., when left on their own, a child's choice is not constructive). Because they are rendered unnecessary (since children have a natural inclination to learn and develop), extrinsic motivators are not employed in Montessori programs. The teacher is able to work with children individually because the Montessori materials themselves do the teaching—they are self‐correcting. And, aligning with children's social tendencies, children are allowed to work together as much as they wish. The only requirement is that children are constructive and that they work through all the materials in a classroom environment during the years when they are in the classroom.

The Montessori system has three elements: the environment, the teacher or guide, and the child (Lillard, 2019a; Lillard, 2019b). The system is adapted for different developmental stages (0‐3, 3‐6, 6‐12, and 12‐18) and cultures (Montessori, 2012). Yet, because children are biologically the same everywhere and have been for many thousands of years (essentially speaking), the system is highly consistent, such that today's Montessori classroom in Kyoto looks very much the same as one in the highlands of Bhutan, the slums of Mexico City, following nomadic tribes in Kenya, or in 1910 in Rome. Ideally, Montessori classrooms have ample natural light and access to nature (plants and animals in the room, and/or easy access to an outdoor space). Within carefully prepared classroom environments, children encounter an array of brightly colored hands‐on materials, one of each type, arranged neatly on accessible shelves into classroom areas (Math, Language, Music, Art, Sensorial Activities, and so on). Each material is available to every child once they have been taught to use it. Material sets increase in difficulty, serving the youngest to the oldest children in each classroom.

The teacher's role in Montessori is to connect children to the environment by showing them (individually or in small groups) how to use the materials, at a time when each child is judged to be ripe to learn them (Elkind, 2003; Jones, 2005; Montessori, 1964; Montessori, 2012; Murray, 2010). The teacher spends a great deal of time simply observing the children, judging their ripeness, and figuring out when and how to stoke a child's interest. Teachers undergo a long preparation for their roles, learning about the Montessori philosophy and theory, the subject matter of the classroom, and how to present each material in what is deemed to be a clear and captivating way, as well as developing sensitivity to how children express readiness to learn. Montessori teachers keep records of each child's progress through the sequences of materials, overseeing their learning.

Although often thought of as a private school model for preschool, Montessori actually goes through high school and is also implemented in the public sector. Most of the over 500 public Montessori schools in the United States are Title 1 schools serving children of color (Debs, 2019). Initiatives like Educateurs sans Frontières are increasingly bringing Montessori to the global majority.

### 2.3 How the intervention might work

Montessori education includes philosophical and structural elements; the structural elements were judged by Maria Montessori to be the best way to implement her philosophy. The intervention might work through either or both of these avenues.

Nine philosophical elements of Montessori were described (along with supporting research) in *Montessori: The Science Behind the Genius* (Lillard, 2017). We briefly summarize these here, as important means through which the intervention might work. For an alternative model of how the intervention might work, see the logic model developed by Culclasure and colleagues (Culclasure, 2019).

1. Montessori education involves hands‐on learning; cognition and movement are therefore deeply aligned (see also Laski, 2015). Children use materials that convey important concepts and skills that might transfer to improved academic outcomes.
2. Children in Montessori get to pursue what they are interested in learning at the moment, rather than something a teacher (or state legislature) has chosen for an entire class to learn at once, at a particular moment in time. Interest enhances learning, and being able to do what one is interested in doing also could lead to better nonacademic outcomes, such as positive feelings about or wellbeing in school (Ryan, 2000).
3. Children in Montessori programs choose what they will learn about; they determine how they will spend their time. Research has shown that when children are in environments with more self‐determination, their academic performance improves (Cordova, 1996; De Charms, 1976). In addition, so does their perceived self‐worth, mastery orientation (Ryan, 1986), and creativity (Amabile, 1984).
4. Montessori also places a high priority on concentrated attention and developing executive function (see Diamond, 2011). Enhanced self‐regulation early in life predicts a wide range of health‐related and wealth‐related outcomes later in life (Moffitt, 2011).
5. Learning stems from intrinsic motivation; there are no extrinsic motivators encouraging children to work in Montessori classrooms. Intrinsic motivation is desirable in itself, and is also associated with lifelong learning (Cordova, 1996; De Charms, 1976; Ryan, 1986). Creativity is also enhanced by a lack of extrinsic rewards (Amabile, 1984).
6. Montessori learning is situated, so a child who is interested in bugs will study actual bugs, not just read about them in texts. In other cases, Montessori's specially‐developed hands‐on materials make learning situated; e.g., a child learning the Pythagorean theorem gets materials that make the theorem self‐evident. The children also embody the theorem in the schoolyard, using ropes to measure triangles, imagining themselves as ancient Egyptians measuring property lines. Learning is enhanced when it is situated in contexts (Cordova, 1996; Lillard, 2017).
7. In Montessori classrooms, children are able to work with peers at will; they learn through imitation, through collaboration, and through peer tutoring. Peers can inspire children to assimilate and accommodate academic and social skills exhibited by those peers (Turner, 1992). Many studies show that peer learning is associated with better outcomes (Topping, 2005).
8. Montessori teachers are counseled to work with children in specific ways, to cultivate the sensitive responsiveness that leads to secure attachment, and to take an authoritative approach; such approaches predict better child outcomes (Baumrind, 1989). Montessori teachers also facilitate student‐driven creative approaches to solving problems (Ultanir, 2012).
9. The Montessori environment is tightly ordered, with everything in its place. Although children have considerable freedom about how to use their time, exactly how a child uses each material is far from random; there are a series of prescribed steps, from which children are permitted to deviate only when the teacher perceives that deviation to be constructive for their learning and development. Order is also associated with better academic and nonacademic outcomes for children (Lillard, 2017).

An educational environment that embodies any or all of these philosophical elements might be expected to result in better outcomes since individual research studies involving each element individually have resulted in better outcomes. Well‐implemented Montessori has all nine of these elements, and thus might improve developmental outcomes.

Over her lifetime, as she developed these philosophical elements, Maria Montessori also arrived at a specific pedagogical structure that she believed was optimal for delivering the pedagogy. When the Montessori structure within which these philosophical elements are intended to be embedded is also included in the intervention, it might further enhance outcomes. These stuctural elements are listed below (Lillard 2019a, 2019b).

1. Teachers who are well‐trained to carry out the intervention, who have learned to be sensitively responsive and authoritative in their implementation of the philosophy, have learned to deliver well the full set of Montessori lessons for the age group they are teaching and know how to tend to the carefully prepared Montessori environment. Thorough Montessori teacher training takes a year and comes from teacher trainers who have undergone an extensive decade‐long preparation to convey the philosophy and approach to others.
2. Classrooms that have children of specific 3‐year‐age spans that are thought to embrace developmental stages (thus all the children in the classroom need particular materials and lessons) and are also thought to be particularly conducive to peer learning.
3. A full set of specially‐designed Montessori materials that enables hands‐on learning and embodied cognition. These materials might also enhance interest, situate cognition, and evoke a sense of order.
4. A 2.5–3 h work period in the morning and afternoon, during which children exercise free choice and concentrate deeply, might assist in the development of executive function.
5. A classroom composition in which there are few adults (and only one trained teacher) and many children, to allow for peer learning and self‐determination. Montessori's ideal ratio was about 1:35, with possibly a nonteaching assistant for young children.

These structural elements are part of what is typically considered high‐fidelity Montessori, but not all Montessori interventions include them. By contrast, the philosophical elements are likely to be in any intervention that is designated as Montessori.

Although we have described the ideal Montessori intervention based on descriptions in Montessori's books, implementation varies in the real world (Daoust, 2004; Daoust, 2018/; Daoust, 2019). Likewise, implementation would be expected to vary in research studies. The intervention being assessed here is Montessori as it appears in the body of research that purports to study it, which reflects the variation of Montessori in the real world. The actual implementations described in the included studies are covered in the Types of interventions section.

### 2.4 Why it is important to do this review

It is important to do this review because better evidence is needed for policymakers and parents to know if the Montessori system produces better or worse outcomes than business‐as‐usual approaches; no prior review has provided definitive evidence. We found only one quantitative meta‐analysis of Montessori education and it included only two studies of Montessori (Borman, 2003). This study reviewed nearly 30 comprehensive school reform programs and concluded that Montessori education serves as a reform with “promising evidence of effectiveness,” *d* = 0.27 [95% confidence interval (CI): 0.19, 0.35], *p* < 0.01. Other school reform programs that were also classified as having promising evidence of effectiveness in the Borman review were America's Choice, Atlas Communities, Paideia, and the Learning Network. Besides including very few Montessori studies, the Borman review only provided outcomes based on national standardized tests of academic achievement.

There have been several narrative reviews related to Montessori education's impact on academic and nonacademic outcomes, and they have focused especially on preschool‐aged children, termed *primary* in Montessori circles (e.g., Ackerman, 2019; Boehnlein, 1988; Boehnlein, 1990; Boehnlein, 1996; Jones, 2005; Marshall, 2017; Murray, 2010). Murray's (2010) narrative review of early (1960s and 1970s) and contemporary (2000s to present) Montessori research revealed that recent studies that employed improved statistical methods demonstrated results favorable to Montessori education. Jones (2005) reviewed Montessori education outcomes research in three areas: (1) the effects of the Montessori approach to at‐risk students, (2) the effects of the Montessori method on exceptional learners including learning disabled, developmentally delayed, and gifted/talented, and (3) comparative analyses of traditional schooling versus Montessori in student achievement and social development. Although thorough, Jones failed to take a systematic approach to searching the literature and did not quantitatively synthesize the research data. Boehnlein (Boehnlein, 1988; Boehnlein, 1990; Boehnlein, 1996) reviewed the Montessori education research that may be of interest to public schools, summarizing the results as follows:

- Early research provides evidence that the Montessori method and environment are beneficial to low‐ and middle‐SES children.
- Current research corroborates the early findings, in particular, the importance of the Montessori preschool experience.
- Of specific importance for best results long‐term are the full 3‐year preschool program, trained Montessori teachers, and multi‐age grouping (Boehnlein, 1988, p. 476).

Although relatively thorough, the Boehnlein reviews were narrative reviews without a systematic search strategy or quantitative synthesis. The same is true of recent narrative reviews by Ackerman (2019) and Marshall (2017). In sum, the narrative reviews of Montessori education lack the systematic quality and rigor afforded by a Campbell Collaboration review, and a systematic review of all the existing literature is needed to resolve the question of whether Montessori has an impact on child outcomes.

## 3 OBJECTIVES

The primary objective of this review was to examine the effectiveness of Montessori education, compared to traditional education, in improving academic and nonacademic outcomes for prekindergarten to high‐school‐aged students. The secondary objectives were to determine which of the following factors moderate the reported effectiveness of Montessori education: grade level, public versus private Montessori settings, type of assignment to experimental and contrast conditions, treatment duration, and length of follow‐up measurements.

## 4 METHODS

### 4.1 Criteria for considering studies for this review

#### 4.1.1 Types of studies

The Campbell protocol for this review can be found in Randolph (2016).

In this subsection, we describe the inclusion and exclusion criteria based on study design features.

- We included studies that used group experimental (i.e., with random assignment) and/or quasi‐experimental (i.e., without random assignment) research designs.
- We included pretest‐posttest with control group designs, posttest‐only with control group designs, and designs with case‐control matching on a measure of the same construct as the outcome construct.
- We excluded pretest‐posttest without control group designs. Studies that used single‐participant, correlational, quantitative descriptive, or qualitative designs were excluded. The portions of mixed‐methods studies that met study criteria were included and the other information was excluded.
- We excluded experimental/quasi‐experimental studies if they did not meet the following What Works Clearinghouse's (2014) study quality standards. For experimental and quasi‐experimental designs, those standards are listed below:
  o Group membership was determined through a random process, or
  o Equivalence was established at the baseline for the groups in the analytic sample.

Because of the potential for selection bias, we used strict criteria for establishing baseline equivalency in studies not using random assignment. Quasi‐experimental studies had to meet at least one of the following criteria to be considered for inclusion:

- The authors used covariate‐adjusted means where at least one covariate was a measure of the same construct as the outcome. For example, we would consider a study with a mathematics outcome to have baseline equivalency if the authors adjusted for mathematics pretest scores. However, we did NOT consider a study as having baseline equivalency if it only adjusted for covariates that are correlated with the outcome. For example, we would not consider a study with a mathematics outcome to have baseline equivalency if it only adjusted for family income, although family income is known to correlate with academic achievement (Duncan, 2014). In short, covariates had to measure the same construct as the outcome for the study to be considered to have baseline equivalency based on covariate adjustments.
- The authors matched participants based on a covariate that measures the same construct as the outcome construct.
- The authors used gain scores to establish baseline equivalency. The pretest and posttest scores had to use the same measure or an equatable, scaled measure.
- The authors provided evidence that there was not a statistically significant difference in pretest scores between Montessori education and traditional education groups.
- We excluded studies in which the author did not report enough information to compute standardized mean difference effect sizes. We did not include studies that required us to impute means and standard deviations from medians and ranges/interquartile ranges. For studies published since 2000, we attempted to contact authors to get this information if it was not reported in the study and kept documentation of that information in the inclusion/exclusion data set provided in Randolph (2021).

Studies were included in the current review if they described their intervention condition as *Montessori*. Not every study specified the elements of Montessori that were implemented and those that described it to varying degrees. The descriptions provided yielded implementations ranging from full (well‐aligned with what is described in Montessori's books; 10 studies) to weak (four that were merely add‐ons to otherwise traditional programs). This range reflects the wide range of implementation of programs called Montessori in the real world: the term is not trademarked. To be objective, the label alone was used to determine study inclusion. In the Types of Interventions section, we report an analysis of the range of implementations used in the included studies; we also note our grade of the implementation quality in the Intervention characteristics column of Table 1.

**Table 1:** Characteristics of included studies.

Study	Participants	Design	Intervention characteristics	Outcomes/measures	Dates	Funding sources
Alburaidi 2019	62 4th‐grade students in a Middle Eastern (Oman) primary school	Quasi‐experimental PPCG design	Nonspecific Montessori approach; add on to conventional program; use of sensorial learning stations	Science achievement as measured by a researcher‐made test in the curriculum area	2017–2018 academic year	None specified
Ansari 2014	7045 Hispanic and 6700 Black low‐income students in two types of Title‐1 Public preschools in Miami, Florida	Quasi‐experimental PPCG design	Montessori classrooms have serious implementation flaws: single age (4), not the recommended 3‐year age span (3–6). Teachers were Montessori trained	Cognitive, Language, and Fine Motor skills (Learning Accomplishment Profile‐Diagnostic). Parent and Teacher Report of Social Skills and Behavioral Problems (Devereux Early Childhood Assessment)	Not specified	Project funded by the Early Learning Coalition of Miami‐ Dade/Monroe and supported by Grant T32 HD007081‐35 (PI: Kelly Raley) provided by the Eunice Kennedy Shriver National Institute of Child Health and Human Development
Aydoğan 2016	35 preschool children (14 boys, 21 girls) in Turkey	Quasi‐experimental PPCG design	Nonspecific Montessori. The intervention lasted 7 weeks	Children's language development was measured by DVT (Descoeudres Vocabulary Test), VLT (Vocabulary and Language Test), and the PPWT (Peabody Picture‐Word Test—Turkish version of PPVT)	Not specified	None specified
Besançon 2013	*N* = 80 6–11‐year‐olds. Paris	Quasi‐Experimental PPRM, test‐retest design	Nonspecific Montessori including theatre workshops; serious implementation issue in using just 1 or 2 ages per classroom	Torrance Test of Creative Thinking (Divergent‐exploratory thinking tasks‐toy improvement and parallel lines; Convergent‐integrative thinking tasks—drawing and story)	Not specified	None specified
Coyle 1968	*N* = 131, 3–5‐year‐olds from 4 schools (low‐income diverse free school, lower‐middle income White neighborhood daycare, upper‐income suburban school with tuition, modified Montessori school with lower‐middle income families who paid a small tuition fee). 72 Montessori students, 59 non‐Montessori	Quasi‐experimental PCG	Private “modified‐Montessori” where 3–5‐year‐olds attend either a morning or afternoon session. Serious implementation issue where classrooms used group instruction. The intervention lasted 12 months with post‐tests occurring at 6 and 12 months; final scores were used here	Five tests of Haptic Perception: Test 1 (identify/name geometric forms), Test 2 (match two geometric forms), Test 3 (match a shape presented in a haptic form to drawing of the same shape), Test 4 (draw a picture of the shape presented haptically), Test 5 (verbally describe the form of the shape presented haptically). Twelve‐month posttest scores used	1965–1967	US Department of Health, Education, and Welfare
Culclasure 2018	*n* = 7401 third through eighth‐grade public Montessori students, the control group was matched for demographics. In the Montessori group, 55% of students were White, 34% were Black, and 6% were Hispanic. 10% of Montessori students had a special education designation	Quasi‐experimental PPRM	All Montessori schools met a minimum standard of fidelity set by the authors; within those included, half scored as high fidelity in classroom observations, and half scored as low or medium fidelity. Considered a medium implementation overall	Writing, ELA, Math, Social Studies, Science, Executive Function, Social Skills, Creativity	2012–2013 school year to 2015–2016 school year	The Self Family Foundation and the South Carolina Education Oversight Committee
Denervaud 2019	*N* = 201 children (99 Montessori (42 girls, 57 boys), 102 Traditional (54 girls, 48 boys)) ages 5–13. Switzerland	Quasi‐experimental PCG	13 classrooms in 5 private AMI Montessori schools in Switzerland. Full implementation	Creativity (Convergent, Divergent), Executive Function (Working Memory, Selective Attention, Cognitive Flexibility), Inner Experience of School (Well‐Being), Language/Literacy, Math	Not specified	National Center of Competence in Research, Swiss National Science Foundation
Denervaud 2020	10 traditional and 13 Montessori schools in Switzerland were selected, *N* = 234 affluent 4–15‐year‐old students (114 girls and 120 boys; 111 Conventional, 123 Montessori). Switzerland	Quasi‐experimental PCG design	13 Swiss Montessori schools meeting AMI structural criteria. Full implementation	Child‐friendly version of the Flanker task: post‐error slowing, post‐error improvement in accuracy	Not specified	The Boninchi Foundation in Geneva, The Department of Radiology of Lausanne University Hospital (CHUV)
Doğru 2015	*N* = 15 5–6‐year‐olds with ADHD or AD, 6 girls, 9 boys; 8 in the experimental group, 7 in the control group. Turkey	Experimental PPCG	Add on to the traditional curriculum. Montessori “tactile boards, sound boxes, binomial cubes, and color tablets”; each material presented and used for 15‐min sessions/day, 3 days/week, for 2 weeks each (8 weeks total)	FTFK Attention Test (Concentration, test–retest)	Not specified	None specified
Elben 2015	*N* = 42 6–7 year‐olds in Switzerland (22 girls, 20 boys). 18 control, 17 Montessori including K. 7 new to Montessori not included	Quasi‐Experimental PPCG	Lower elementary bilingual Montessori classes (analyzed children started Montessori by Kindergarten), accredited by the Swiss Montessori Association, private school. Three‐hour work period in AM. Supplementary “specials” (music, P.E., crafts) in PM. Montessori classrooms have 3 teachers and aides for about 30 students. “Several of the teachers have the Zurich teaching diploma and the Montessori elementary teaching diploma, and the others have Montessori diplomas.” The intervention lasted 6 months. Full implementation	ELFE 1–6 (reading comprehension); other reading measures (phoneme—Bako; letter recognition) given but not used (no pretest for latter)	November 2014–May 2015	None specified
Faryadi 2017	180 Malaysian Kindergarteners	Quasi‐Experimental PPCG design	Unspecified Montessori school. Photographs in the article suggest a mix of Montessori and non‐Montessori materials. The intervention lasted 4 months. Medium implementation	Math ability, using a variety of measures to assess components of Critical Thinking, Problem Solving, “responsible learners”	2015	None specified
Fleege 1967	*n* = 21 Montessori (13 boys, 8 girls) matched for IQ, SES, and a range of other variables with 21 control at a non‐Montessori preschool in the same high‐income Chicago community	Experimental PPCG	Montessori pre‐school; no details given on implementation	PPVT administered at the beginning and end of the school year	1963	Office of Education, US Department of Health, Education, and Welfare
Galindo 2014	600 low‐income bilingual prekindergarten students (300 Montessori, 300 Traditional) ages 4–5 years in Houston, TX. The majority of students in both centers were Hispanic and low‐income (1% White students in Montessori, 0.7% White in Traditional)	Quasi‐Experimental PPCG design	Public bilingual Montessori with 36 classrooms; 94% had some Montessori training, but intervention quality is unknown. The intervention lasted 26 weeks	BBCS:E (Bracken School Readiness) test of concept development administered at the beginning and end of the school year	2012–2013	None specifies
Hoseinpoor 2014	60 preschoolers ages 5–6 in Iran	Quasi‐Experimental PPCG design	Add on to the conventional program. The experimental group went through a series of 12 lessons involving some Montessori materials and practices	Wechsler Intelligence Scale (Attention and Concentration), 4th ed. Wiland Questionnaire of Growth of Social Skills Scale	2013–2014 school year	None specified
Jones 1979	6th and 7th grade follow‐up included 18 who attended M at age 4 (all Black, 75% single parent homes); 24 attended traditional preschool (90% Black; 45% single parent homes); Louisville Head Start study (lower‐SES)	Experimental PPCG	As described in Miller, Dyer, et al. 1975 SRCD monograph. Consultants rated program implementation 6.5/10. Only 4‐year‐olds in the program and teacher training very limited	Vocabulary, Reading Comprehension, Word Study Skills, Math Concepts, Math Computation, Math Applications, Spelling, Language (measures taken from Weschler Intelligence Scale for Children ‐ Revised and Stanford Achievement Test—6th and 7th‐grade points)	1968–1969	None specified
Juanga 2015	Two classes (each *n* = 120) of Kindergarten students in each of two schools (one private, one public) ages 5–6 years. Not all students at each school took both tests. Philippines	Quasi‐Experimental PPCG	The researcher and assistant implemented Montessori workstations in traditional classrooms with help of a Montessori consultant, focusing on science and math. Length of intervention unclear. It might have been a single day/lesson or add on	Math and science achievement tests created for the study	Not specified	None specified
Kayili 2016a	Montessori (*n* = 19, 6 girls, 13 boys, avg. 3.68 years), Control Group 2 (*n* = 16, 11 girls, 5 boys, avg. 3.63 years). Turkey	Experimental PPCG	3–6 Montessori classroom assoc with Selcuk U. in Turkey; other features unclear resulting in unknown implementation classification. A third group was a Montessori classroom supplemented with social skills training composed of 64 lessons; pretest and posttest were around that intervention, with timing unclear	Social (Wally Feelings Test, Wally Social Problem Solving Test)	2013–2014 school year	None specified
Kayili 2016b	63 children 48–72 months old, in Montessori (*n* = 40, 19 girls, mean age 63.33 mos) or Preschool Education Program created by the Ministry of National Education (*n* = 23, 13 girls, mean age 63.61 mos) at a single school. Turkey	Quasi‐Experimental PPCG design	See the other Kayili 2016 study; 3–6 Montessori classroom assoc with Selcuk U. in Turkey; other features unclear. Tested at the beginning and end of the school year	Kansas Reflection‐Impulsivity Scale for Preschool	2015–2016 school year	None specified
Kirkham 2017	40 students ages 6–11. 20 Montessori (8 girls avg. age 92 mos, high SES), 20 National Curriculum (12 girls, avg age 97 mos, lower SES). UK	Quasi‐Experimental PPRM design	4 Classrooms in 4 Montessori schools accredited by the UK Montessori Schools Association (MSA) with additional input from the Montessori St. Nicholas Charity. One classroom had fantasy toys (like dolls) available	Test of Creative Thinking—Drawing Production. Groups were equivalent on Expressive Vocabulary Test and Raven's. Also administered a pretense production task	Not specified	None specified
Lillard 2006	*N* = 112: 5‐year‐olds (25 control (15 boys, 10 girls), 30 Montessori (15 boys, 15 girls)); 12‐year‐olds (28 control (18 boys, 10 girls), 29 Montessori (12 boys, 17 girls)), in Milwaukee WI schools serving a diverse population. The controls were at 27 public inner city schools (40 children) and 12 suburban public private/voucher or charter schools (13 children). Both control and Montessori children had similar family income (average $20,000 to $50,000 annually)	Lottery controlled, PCG	Public Montessori schools recognized by AMI. Full implementation	Woodcock‐Johnson IIIR Applied Problems, Picture Vocabulary, Letter Word, Word Attack, False Belief, Social Problem‐Solving Test, Dimensional Change Card Sort, Playground behavior, School Liking, Narrative task	2005–2006 school year	Jacobs Foundation, Ovid Foundation
Lillard 2017	*N* = 141 Hartford, CT preschool students, 70 in public Montessori Schools and 71 in Non‐Montessori Schools, followed 3 years. Age 41.15 months avg. at start. 3 cohorts. Half White, half higher income	Lottery controlled PPCG	11 Montessori 3 to 6 classrooms with AMI‐trained teachers in 2 AMI‐affiliated public magnet schools. The intervention lasted for 3 years and the children were tested four times. Full implementation	Woodcock–Johnson IIIR Letter Word, Picture Vocabulary, Math (composite of Applied Problems and Calculation), Theory of Mind Scale, Rubin's Social Problem‐Solving Test, Executive function (Head‐Toes‐Knees‐Shoulders task and Copy Figures from the Visuospatial Processing section of the NEPSY‐II), Mastery orientation (puzzle task), School enjoyment (preference questionnaire), and Creativity (Alternative Uses)	2010‐2015	Brady Education Foundation
Lillard 2012	*N* = 172 3–6‐year‐olds enrolled in private Montessori and conventional private schools where Montessori parents said would send children if Montessori was not available. Majority White (90%), 5% Black, 3% Asian, 1% Hispanic	Quasi‐experimental PPCG	All teachers in the Classic Montessori group were all AMI‐trained. Tested children at the beginning and end of the school year. Full implementation	Executive function (Head‐Toes‐Knees‐Shoulders, Theory of Mind Scale, Social Problem Solving Task, Woodcock‐Johnson IIIR Applied Problems, Letter Word, Picture Vocabulary	Not specified	None specified
Mallett 2015	*N* = 1035 students (518 at 2 Montessori schools, 517 non‐Montessori), 1st through 5th grade, approx. 100 at each age level in each group, urban Texas public school district	Quasi‐experimental, PCG	Montessori classrooms had a “full array of specialized Montessori materials.” Montessori teachers also held or were training for Montessori teaching certification. Montessori classrooms had both a teacher and a teaching assistant; adult child ratio same as in all public district schools. 50% of incoming Montessori grade 1 students had no prior Montessori experience. Medium implementation	First and second graders: Iowa Tests of Basic Skills (Reading Vocabulary and Reading Comprehension made up “Reading,” Math Concepts and Math Computation made up “Math”), Third, fourth, and fifth graders: Texas Assessment of Knowledge and Skills. Control for SES, race, gender, and prior year's scores	2011	None specified
Manner 1999	Third‐graders in Broward Country, Florida. Students either went to a Montessori magnet school or a traditional school, both with diverse student bodies. Matched for the third‐grade score in either math (30 pairs) or reading (37 pairs), followed through 5th grade. Within the math group, there were a total of 60 students, 30 pairs, and within the reading group there were 74 students, 37 pairs	Quasi‐experimental PPRM	The study included public magnet Montessori schools with teachers with AMS training completed or in progress. The intervention lasted for three years with the same participants. Medium implementation	Stanford Achievement Tests Total Reading and Math scores. The author only reported combined math across the three years, but reading was separated out	1996–1998	None specified
Miller 1983	Recovered 20 Montessori and 29 Traditional preK Head Start participants in 1978 when in 8th grade; able to view 7th‐grade test scores too. About 90% of the students in the experimental group (students who were assigned a program) were Black and came from low‐income families	Experimental PPCG	See Jones above	Seventh grade: WISC‐R. Eighth Grade: Stanford‐Binet Intelligence Test	1978 follow‐up; intervention in 1965	None specified
Miller 1984	Recovered 20 Montessori and 22 Traditional preK Head Start participants in 1980 when in 10th grade. About 90% of the students in the experimental group (students who were assigned a program) were Black and came from low‐income families	Experimental PPCG	See Jones above	STEP‐Locator achievement tests and the Comprehensive Test of Basic Skills	1979–1980	None specified
Mix 2017	Experiment 2 sample included 68 children ages 5 and 7, half attended a Montessori school since age 3, half attended a non‐Montessori preschool and one of the three elementary schools (2 public, one private) in the same community	Experiment 1‐ Quasi‐experimental PPCG; Experiment 2‐ Quasi‐experimental PPRM	Experiment 2‐ Three enduring (25 years plus) private Montessori schools. One AMI certified and at others all teachers had AMI or AMS training. Full implementation	Children matched for PPVT. Place value, number line estimation, number ordering, number interpretation, school sale, and multi‐digit calculation	Not specified	The research was funded by a grant from the Institute of Education Sciences
Prendergast 1969	43 children in a Montessori, 41 conventional, and 42 children who did not attend either. All children were from upper‐middle‐class families	Quasi‐experimental PPCG	The intervention lasted 7 months. The Montessori schools were all members of a local association. “The children's experiences followed as closely as possible the activities developed by Maria Montessori.” Medium implementation	Eye‐hand coordination, Figure‐ground, Position in space, Auditory discrimination, Receptive language	1966–1967	None specified
Rathunde 2005a	Montessori (150 students, 60% female, 40% male, 72.6% white, 10.2% Asian, 12.7% Black, 1.9% Latino, 2.6% other races, majority of students from suburban schools in middle‐ or upper‐middle‐class communities). Traditional middle school students (*N* = 400, half of the students from “ethnic minority families,” matched a subset of this group that was mostly white and middle‐ or upper‐middle‐class to the Montessori group, *n* = 160, 55% female, 45% male, 74.9% White, 7.8% Asian, 3.6% Latino, and 1.2% other race)	Quasi‐experimental PPRM	Five Montessori schools in four states, selected with consultation with the North American Montessori Teachers Association. AMI teachers. Full implementation	Student Perceptions of their Schools and Teachers (support, order, safety, fairness), Time Use at School, Classroom Activities (passive listening, collaborative work, individual work, media), Time with Friends, Classmates, Teachers, and Alone, Classmates and Friends	Unspecified, but data collection at the traditional schools occurred several years before data collection at the Montessori schools	The O'Shaughnessey Foundation, Dekko Foundation, and Hershey Foundation
Rathunde 2005b	See Rathunde 2005a	Quasi‐experimental PPRM	See Rathunde above	Affect (happy, relaxed, sociable, proud), Potency (strong, active, excited), Intrinsic Motivation (e.g., Did you enjoy what you were doing?, Salience (e.g., Was this activity important to you?)	Unspecified, but data collection at the traditional schools occurred several years before data collection at the Montessori schools	The O'Shaughnessey Foundation, Dekko Foundation, and Hershey Foundation
Tobin 2015	66 refugee children (ages 3–6) in two classrooms, one traditional Thai teacher‐directed (*n* = 27) and one Montessori (*n* = 29)	Quasi‐Experimental PPCG	The intervention lasted 54 months. Both Montessori and traditional classrooms have 60 children, one teacher, and one assistant. Unknown implementation	54‐month Ages and Stages Questionnaire: Fine and Gross Motor, Communication, Problem Solving, Personal‐Social	Not specified	None specified
Yussen 1980	Experiment 1: *N* = 60 4–5‐year‐olds. Half attended Montessori, half attended other private nursery schools. Half boys in each group. Middle and upper‐middle‐class families. Madison, WI	Quasi‐Experimental PPRM	The Montessori schools were AMS or AMI certified. Full implementation	Experiment 1‐ Communication was measured by social cues in different trials, mean length of utterance (MLU) and syntactic complexity of utterances. Emotional perspective–taking measured with tasks to identify and explain different emotions. Peabody Picture Vocabulary pretest for equivalence	Not specified	A grant was received by the Wisconsin Research and Development Center for Cognitive Learning which is further funded by the National Institute of Education. Financial assistance was given by the Spencer Foundation

*Note*: None of the included studies had disclosed any declarations of interest.

Abbreviations: PCG, posttest‐only with control group design; PPCG, pretest–posttest with control group design; PPRM, pretest–posttest with matched controls.

#### 4.1.2 Types of participants

We included studies in which the participants were in preschool, elementary, middle school/junior high school, and/or high school. When a study had participants in two or more of these groups, we classified the age group for the study as the age group with the greatest number of participants. See the coding book in the supplemental information (Randolph, 2021). The “location, setting, status, or definition of the condition and demographic factors” (Campbell Collaboration, 2019b, p. 8) were not considered as inclusion or exclusion factors. We created some exploratory emergently‐coded variables for demographic factors (e.g., gender, family income, etc.) and country of origin, but those factors were not included as moderators in this analysis. See the Data extraction and management section for more details on the demographic information collected.

#### 4.1.3 Types of interventions

The intervention was defined broadly as Montessori education. We operationalized Montessori education as an intervention in which the study authors claimed to have used the Montessori method of education; this occurred in both public and private school settings. In all studies, Montessori education was a separate measurable intervention. Traditional or business‐as‐usual education was the comparison condition for all studies in the analysis. Next, we describe what these interventions were in the included studies; they align with the range of implementations of Montessori and traditional education in the real world, given that neither term is trademarked.

##### Montessori interventions

Although the included studies designated the intervention condition as “Montessori,” the studies' methods sections provide varying levels of description of the intervention and although most of those descriptions indicated that the intervention had the philosophical elements of Montessori education, some revealed that they deviated in certain ways from the structural ones. Because of this variation, in response to the first set of reviews we categorized (post‐hoc) the Montessori conditions into five categories. All of the Montessori interventions appeared sensitive to the philosophical elements of Montessori, for example touching on free choice and hands‐on materials in the article Introduction if not also in Methods. Where they varied the most was in structural elements.

Although we performed this categorization, we do not advise considering how effect sizes might vary with the implementation levels because effect sizes stem from many sources including the different effectiveness of the Montessori intervention relative to its control condition; the control or traditional conditions also varied, as they do in the real world. A second reason not to consider these levels for analysis is that the variables that led to the categorizations are our best estimates based on what was reported; they lack precision. In sum, we provide the levels and their descriptions here only to give readers a sense of the range of implementations that were considered in the meta‐analysis.

At the highest level was full implementation, meaning that the school was recognized by a respected association like the Association Montessori Internationale (AMI) or the Swiss Montessori Association or at least had teachers who were fully trained by AMI or the American Montessori Society before the intervention took place. AMI recognition entails the structural elements of having AMI‐trained teachers (trained to implement the philosophy to a high degree), a specific 3‐year‐age range in each classroom (e.g., children ages three to six or six to nine), a 2.5–3 h uninterrupted work period during which children are free to choose their own work, large class sizes and few adults, and a full set of Montessori materials. There are also no grades; the materials are self‐correcting. The Montessori condition in 10 of the 32 studies appeared to meet these criteria (Denervaud, 2019, 2020; Elben, 2015; Lillard, 2006; Lillard, 2012; Lillard, 2017; Mix, 2017; Rathunde, 2005a; Rathunde, 2005b; Yussen, 1980).

The next highest level, observed in six studies, was medium implementation; for these, the article mentions some accreditation or teacher training, but the accrediting organization was unspecified (“a local Montessori association,” Prendergast, 1969), and teacher training was done by a respected organization like AMS but not all teachers had completed the training at the time of data collection (Mallett, 2015). In one case the description of Montessori suggested good implementation, but photos included in the article incorrectly called some commercial toys “Montessori materials” (Faryadi, 2017), which put the study in a lower category. In another case, implementation was discussed and a rubric was developed to measure it, and the measure indicated a nontrivial level of deviance from the highest level of implementation among the schools studied (Culclasure, 2018). In another case, the UK Montessori Schools Association had accredited the Montessori schools, but they deviated by including pretend play; this may not be a major deviation but did suggest not fully implementing Montessori (Kirkham, 2017). One study considered to have a Montessori condition at this medium level had AMS‐trained teachers or teachers in training but used only two ages of children per class instead of three (Manner, 1999).

At the next level, observed in seven studies, Montessori was claimed to be implemented as a full classroom program, but there was at least one very serious deviation from the full implementation of the structure, and other elements were unspecified or suggested other problems. For five of these, classrooms had only one age level: 4‐year‐olds (Ansari, 2014; Galindo, 2014; Jones, 1979; Miller, 1983; Miller, 1984); three of these also had teachers with only a 6‐week training course. For one study, it was stated that some classrooms had mixed grades and it specified two grades, suggesting some other classrooms had only single ages (Besançon, 2013). Another study at this level called the intervention *modified Montessori* and stated that some children had group instruction during part of the session (Coyle, 1968; description taken from Concannon, 1966). These all suggest some serious deviations from high‐fidelity Montessori.

There were five studies in which the level of Montessori implementation could not be determined: Fleege (1967), Kayili (2016a), Kayili (2016b), Tobin (2015), Aydoğan (2016). In these studies, Montessori philosophy was described appropriately, but there was not enough information about the classrooms themselves to determine whether the structural elements were in place; one was left with very little sense of how Montessori was actually implemented. In the fifth study in this category, not only was the Montessori implementation unspecified, but the intervention appeared to occur over a 7‐week period (Aydoğan, 2016); it is unclear if children had had Montessori programming before the 7‐week‐long study.

Finally, there were four studies in which the Montessori condition appeared to be an add‐on curriculum implemented for a limited period of time. Alburaidi (2019) set up a school Science Hall that had six centers offering hands‐on materials, choice, meaningful activities, order, and other Montessori characteristics; intervention students spent their 45‐min science classes covering specific topics in this hall with a teacher taught to guide in a Montessori manner. Doğru 2015 taught children with ADHD to use Montessori sensorial materials (e.g., tactile boards, binomial cubes) over 8 weeks, for 45 min each week. Hoseinpoor (2014) offered 12 sessions with a variety of sensorial activities and role‐play activities; it appeared that these built on each other and that each session included the activities from prior sessions. From their introduction, it seems the intervention children were free to choose among these activities. The final study in this group (Juanga, 2015) implemented Montessori for a single day using assigned workstations; the environment was prepared, the activities were hands‐on, and a Montessori consultant guided the teacher.

Although there is tremendous variety in these implementations, they reflect the variety of implementations of Montessori in the real world; the term is not trademarked.

##### Comparison condition: Traditional education

Detailed descriptions of the comparison condition were rarely provided, as if the terms *conventional* or *traditional* made clear what they were. At the elementary school level (studies with children ages 6–12), some studies specified that the control condition had teacher‐directed, whole‐class learning involving textbooks, teachers checking and grading work, single‐aged classrooms, and class periods of limited duration (in some cases 45 min). Most studies outside of the United States referred to a national program (such as traditional Swiss or French or Thai pedagogy, or the UK National Curriculum). At the preschool level, some non‐western studies referred to (for example) traditional Malaysian or Philippine, or Iranian conventional education, without reference to a set curriculum; further details suggested these were also teacher‐centered, lecture‐style approaches. By contrast, for some (especially older) studies conducted in the United States, traditional education at the preschool level was described as comprised of free play and pretense (Coyle, 1968; Miller, 1983; Miller, 1984; Prendergast, 1969). One study specified that its conventional condition implemented HighScope, which involves centers offering various art, play, and learning activities; this method is modeled after the Perry Preschool Project (Ansari, 2014). Others provided little to no information about their conventional preschool condition, referring simply to the control program as pre‐K, traditional, business‐as‐usual, or non‐Montessori. In recent years preschool programs in the United States are more likely to be teacher‐centered and involve little play (Bassok, 2016).

In sum, traditional and Montessori conditions reflected a range, across studies, that is reflective of the range of implementations of each in the real world. Although we categorized the Montessori implementation into levels post‐hoc, we advise against considering the relation between effect sizes and these estimated levels of implementation, first because our categorization is imprecise, and second because effect sizes reflect the relative difference across Montessori and control conditions in each study, and control conditions varied as well in ways that are impossible to rank precisely.

#### 4.1.4 Types of outcome measures

We included two broad categories of outcomes: academic and nonacademic. Because of the wide range of nonacademic outcomes, we used an emergent approach to arrive at the specific outcomes being measured in the Montessori literature. We considered academic outcomes to be primary measures and nonacademic outcomes to be secondary measures.

##### Primary outcomes

The variety of academic outcomes was reduced to five categories of outcomes:

- General academic ability. This outcome included measures that did not clearly fit one of the other categories (like math or reading), such as the cognitive subscale of the learning Accomplishment Profile‐Diagnostic (Lap‐D) (see Ansari, 2014); this subscale included counting as well as matching and did not report scores separately. It also included three subtests of the Bracken scale (see Galindo, 2014) including identifying colors and comparing sizes. Tests of problem solving that were not explicitly mathematical were also categorized as General Academic Ability. Finally, different academic abilities concatenated into a general academic score were included in this category.
- Mathematics. Grouped for the mathematics analysis were tests of computation and math concepts, tests of number line estimation and of counting, the Woodcock‐Johnson Applied Problems subtest, and tests administered by states to examine math achievement. Most of the math tests were also standardized, normed measures.
- Language/literacy. This cast a broad net around all measures pertaining directly to reading, vocabulary, language use, and language perception, including the Stanford Achievement Tests' language measures, the Woodcock‐Johnson Letter‐Word and Word Attack subtests, and tests of reading comprehension, speech sound discrimination, spelling, and vocabulary. Some tests were administered by states to examine English Language Arts proficiency. Virtually all the literacy tests were standardized, normed measures.
- Science. Just three studies included measures of science achievement using a pre‐post design or controlling for pretest scores. Two studies created science tests for the study; the third, which measured children over 3 years, used the South Carolina state tests of science achievement.
- Social studies. This outcome included measures such as South Carolina state assessment in social studies.

Individually the academic outcomes had too few studies to do a moderator analysis with cluster–robust variance estimation; therefore, we also created an aggregated academic outcomes variable that comprised studies that contributed at least one effect size to one or more of the academic outcomes listed above.

##### Secondary outcomes

Although the nonacademic outcomes are listed as secondary outcomes here, we do not intend to assign a hierarchal structure to academic and nonacademic outcomes. After examining the variety of nonacademic outcomes, we found that there were four major nonacademic outcomes:

- Creativity. This included measures like Alternate Uses, where children must come up with all possible uses for a common object like a paper clip, the Torrance Test of Creativity (which includes Alternate Uses as one of several tasks), and tests where children need to create a drawing or story and a panel of judges rates their creativity.
- Executive function. Executive function was measured directly with a wide variety of tasks and also indirectly with parent or teacher report questionnaires. The direct tasks include, for example, the Simon‐Says‐like game “Head Toes Knees Shoulders”, the Flanker task, and reciting a string of digits backward. An example of a teacher‐ or parent‐report measure of executive function is the Behavior Rating Inventory of Executive Function or BRIEF.
- Inner experience of school. Some studies addressed how children experience school, asking how much they like school on a survey or using the experience sampling method, whereby pagers randomly beep children and ask them to rate their immediate emotional and cognitive experience; in such studies, the in‐school data were used from children attending different types of schools.
- Social skills. Included in this category were both tests assessing children's social knowledge, including tests of basic social cognition (like tests of emotion recognition and the Theory of Mind scale) and tests assessing knowledge about managing peer relations (e.g., asking how one would respond to a social conflict, as with Rubin's Social Problem Solving test). Also included were teacher and parent ratings of children's social behavior (such as the Deveraux Early Childhood Assessment), and live coding of social behavior (e.g., ambiguous rough and tumble play on a playground).

Individually, the nonacademic outcomes had too few studies to do a moderator analysis with cluster–robust variance estimation; therefore, we created an aggregated nonacademic outcomes variable from studies that contributed at least one effect size to one or more of the nonacademic outcomes listed above.

### 4.2 Search methods for identification of studies

#### 4.2.1 Search methods

The initial electronic search was conducted in March 2014 with no limits on the date of publication. A follow‐up search was conducted in February 2020 to find any studies published since January 2014. (To be cautious, we intentionally created a 3‐month overlap of the first and second rounds of searching.) No geographic limitation was placed on the search. Results were gathered from electronic databases, the open web, online gray literature websites, and directly from authors and experts in the field.

A comprehensive search strategy was designed and implemented by an academic librarian in an attempt to retrieve all experimental and quasi‐experimental studies that adhere to the inclusion and exclusion criteria. We examined both published and unpublished literature. Articles and gray literature were gathered using online databases that cover education, sociology, and psychology, and recommendations from experts in the field of Montessori education. Only results written in English were considered for inclusion, although no language limiters were utilized in the searches. We employed both free‐text and controlled vocabulary terms in the searches. All permutations of search terms were used during the search process. We complemented our search with a thorough examination of reference lists of relevant retrieved studies, both included and excluded, and contacted experts in the field to identify any ongoing or unpublished studies.

Studies were identified using the following electronic databases and online sources:

- Academic Search Complete (EBSCO)
- AERA Online Paper Repository
- American Montessori Society Montessori Research Library
- Arts & Humanities Citation Index (Web of Science)
- Dissertations & Theses Global (ProQuest)
- Education Full‐Text/Education Research Complete (EBSCO)
- Education Journals (ProQuest)
- ERIC (EBSCO)
- Google Scholar
- JSTOR
- Open Grey
- PsycINFO (EBSCO)
- Professional Development Collection (EBSCO)
- Research Library (ProQuest)
- Social Sciences Citation Index (Web of Science)
- Social Sciences Journals (formerly Social Science Database) (ProQuest)
- SocINDEX with Full Text (EBSCO)
- Sociological Collection (EBSCO)
- Teacher Reference Center (EBSCO)

During the search process, we utilized phrase searching and truncation methods to find all variations of relevant search terms. Database thesauri, when available, were used to find controlled vocabulary descriptors and related descriptors which were integrated into search iterations.

The search strategy was customized as needed for each database and was changed to include Montessori classrooms of all grade levels. Details on the search strategy for each source are provided in the Appendix. Search strategies were tailored to the unique controlled vocabularies of each database and were used in conjunction with free text search terms, which can be found in Supporting Information: Appendix 1. The search strategy included keywords and subject headings pertaining to setting (school), intervention (Montessori), and outcome (academic and nonacademic). The number of search results from each source is provided in Table 2.

**Table 2:** Search results by academic database.

	Records for initial search	Records for follow‐up search
*Database*		
EBSCO Academic Search Complete (1887–current)	11	12
EBSCO Education Full‐Text [Education Research Complete] (1881–current)	85	7
EBSCO PsycINFO (1887–current)	103	21
EBSCO Professional Development Collection (1940–current)	33	10
EBSCO SocINDEX with Full Text (1881–current)	18	5
EBSCO Sociological Collection (1947–current)	5	0
EBSCO Teacher Reference Center (1984–present)	21	3
ERIC [EBSCO and web] (1966–current)	242	97
JSTOR (1800s–current)	46	4
ProQuest Education Journals [ProQuest Education Database] (1991–current)	65	97
ProQuest Dissertations & Theses (1637–current)	52	20
ProQuest Research Library (1971–current)	75	89
ProQuest Social Sciences Journals [Social Science Database] (1994–current)	247	217
Web of Science Arts & Humanities Citation Index (1975–current)	158	61
Web of Science Social Sciences Citation Index (1900–current)	27	33
*Grey literature source*		
AERA Online Paper Repository (2010‐current) https://www.aera.net/Publications/Online-Paper-Repository/AERA-Online-Paper-Repository	16	40
American Montessori Society Montessori Research Library (coverage undetermined) https://amshq.org/Research/Research-Library	8	12
Open Grey [discontinued] (1980‐2020) http://www.opengrey.eu/	10	0
*Internet searches*		
Google Scholar (coverage undetermined) https://scholar.google.com	*titles and abstracts of first 500 results were examined before relevancy dropped off significantly	
Google (coverage undetermined) https://www.google.com	*titles and abstracts of first 500 results were examined before relevancy dropped off significantly	
Bing (coverage undetermined) https://www.bing.com	*titles and abstracts of first 500 results were examined before relevancy dropped off significantly	

There were limitations related to this search. Namely, the search was limited by the omission of studies published since February 2020 and the studies were also limited to English.

#### 4.2.2 Electronic searches

##### Databases

A comprehensive database search included the following online subscription databases:

- EBSCO Academic Search Complete
- EBSCO Education Full‐Text
- EBSCO Professional Development Collection
- EBSCO PsycINFO
- EBSCO SocINDEX with Full Text
- EBSCO Sociological Collection
- EBSCO Teacher Reference Center
- ERIC
- JSTOR
- ProQuest Dissertations & Theses
- ProQuest Education Journals
- ProQuest Research Library
- ProQuest Social Sciences Journals
- Web of Science Arts & Humanities Citation Index
- Web of Science Social Sciences Citation Index

##### Gray literature

Professional Montessori association websites, gray literature, digital repositories, and social science research websites were identified through expert referrals and Internet searches, and conference proceedings, reports, working papers, white papers, and preprints were reviewed to find relevant research. Government documents were identified primarily through the Education Resources Information Center (ERIC) and the US Department of Education's Institute of Education Sciences (IES) and various subsidiaries, projects, and digital libraries. ERIC was searched both using the EBSCOhost platform and government‐hosted websites to ensure an exhaustive search. Websites were searched for relevant studies, reports, and citations. Sources for gray literature included:

- American Educational Research Association (http://www.aera.net/)
- American Montessori Society (https://amshq.org/)
- Association Montessori Internationale (https://montessori-ami.org/)
- Association Montessori Internationale/USA (https://amiusa.org/)
- ERIC (https://eric.ed.gov/)
- Institute of Education Sciences (http://ies.ed.gov/)
- Montessori Educational Programs International (https://www.mepiinc.com/)
- National Center for Montessori in the Public Sector (http://public-montessori.org/)
- OpenGrey [discontinued] (http://www.opengrey.eu/)
  Pan American Montessori Society [discontinued] (The website no longer exists.)
- Social Science Research Network (http://papers.ssrn.com/sol3/DisplayAbstractSearch.cfm)
- Society for Research on Educational Effectiveness (https://www.sree.org/)

##### Internet search

Google, Google Scholar, and Bing were used to search the open web in an attempt to fill in any gaps left after searching the specialized sources. Results were analyzed until a saturation point was reached, (i.e., until further searching led to no new articles for inclusion).

See Table 2 and Supporting Information: Appendix 1 for further information on Internet searches.

#### 4.2.3 Searching other resources

##### Contacting other researchers

Authors of prior studies and other experts in the Montessori method were contacted to obtain unpublished research or to get further clarification on published studies. A record of attempts to contact authors can be found in the notes section of the inclusion and exclusion data set provided in the supplemental information to this review (Randolph, 2021).

##### Prior reviews and reference lists

The prior reviews that were searched are mentioned in the Why it is important to do this review section and there is additional information in the supplemental information provided in Randolph (2021).

During both the search and selection processes, studies determined to be relevant enough for closer inspection of reference lists were searched for citations to additional studies. For example, a study examining Montessori and non‐Montessori outcomes that had insufficient data and was therefore not included would be examined for references to other studies.

#### 4.2.4 Hand search

Hand searches were conducted with available journals and conference proceedings that had not been adequately electronically indexed. The *Journal of Montessori Research & Education, Journal of Montessori Research*, and *AMI Journal* were hand searched by examining tables of contents for all available issues with no data limitations, both in 2014 and 2020.

### 4.3 Data collection and analysis

In general, we followed the data collection methods in the Cochrane Handbook (Higgins, 2021), the MECCIR Conduct Standards (Campbell Collaboration, 2019b), the MECCIR Reporting Standards (Campbell Collaboration, 2019b), metafor documentation (Viechtbauer, 2010), and the cluster‐robust methods described in Tanner‐Smith (2014), Tanner‐Smith (2016), and Tipton (2015). The metafor (Viechtbauer, 2010), metaviz (Kossmeier, 2020), robumeta (Fisher, 2017), and ClubSandwich (Pustejovsky, 2020) packages in R (R Core Team, 2019) were used for all meta‐analyses. Supporting R packages used for data preparation can be found in the R code provided in the supplemental information (Randolph, 2021).

Following the suggestions of the American Statistical Association (Wasserstein, 2019), we refrained from null‐hypothesis statistical significance testing when possible. However, for readers interested in interpreting our results in the null‐hypothesis testing paradigm, we provide Benjamini‐Hochberg‐adjusted critical alpha values (Benjamini, 1995; Benjamini, 2005). These adjusted critical values and confidence intervals are meant to keep the false discovery rate and false coverage rates, respectively, at an overall 0.05 level. We report the adjusted values separately for primary objectives with 11 main‐effects estimates in the supplemental information (Randolph, 2021).

#### 4.3.1 Selection of studies

After the information retrieval expert for this review initially identified potential studies for inclusion based on titles and abstracts, at least two reviewers independently reviewed the full text and/or abstracts of the study to make decisions about study inclusion and exclusion. Disagreements were resolved by consensus.

#### 4.3.2 Data extraction and management

The data specified on the coding sheet were extracted independently by at least two reviewers and a consensus decision was reached when there was disagreement.

Our coding sheet was divided into the following categories: publication characteristics, setting, outcomes, participants, research design (including an indicator of methodological quality and risk of bias), statistical analysis of the study, and characteristics of the effect size. The research design, statistical analysis of the study, and characteristics of effect size were based on the Campbell Collaboration Methods Policy Briefs (Campbell Collaboration, 2019a; Campbell Collaboration, 2019b).

We did not include the risk of bias variables related to blinding because blinding was not possible in the educational studies we reviewed. We assumed a high risk of blinding bias for all studies; therefore, we do not have the risk of bias codes related to blinding. We expected quasi‐experimental studies, so we intended to measure what methods and confounding variables the study authors controlled for.

The list below indicates the main variables used in the coding sheet to extract data. The revised coding sheet can be found in the supplemental information provided in Randolph (2021). This list may differ slightly from the one in the protocol because we used an emergent coding approach to create some variables. We created some emergent codes when we were unsure what categories we would find.

1. Identification of Studies (e.g., study id)
2. Publication and Participant Characteristics (e.g., the format of publication, type of school, grade level of participants)
3. Experimental/Statistical Controls (e.g., covariates)
4. Effect Size Information (e.g., the *g* effect size and its variance)
5. Equivalency at Baseline (e.g., evidence on how equivalency at baseline was established: nonstatistically significant *t* tests on a pretest, gain scores, covariate adjustments.)
6. Effect Size Calculations (e.g., the standardized raw mean change approach.)
7. Notes/other

#### 4.3.3 Assessment of risk of bias in included studies

The RoB 2 tool (Sterne, 2019) was used to assess risk of bias in experimental studies and the Robbins‐I tool (Sterne, 2016) was used to assess risk of bias in nonexperimental studies. Two authors (DKH, AKM) independently applied these tools to each article and reached consensus agreement in the case of disagreement. A third author (ASL) reviewed the accuracy of the risk‐of‐bias ratings. Documentation of risk‐of‐bias ratings can be found in the supplemental information (Randolph, 2021).

RobVis (McGuinness, 2020) was used to create traffic‐light plots. Risk of bias includes allocation (selection bias), blinding (performance bias and detection bias), incomplete outcome data (attrition bias), selective reporting (reporting bias), and other potential sources of bias explained in Higgins (2021). A helpful table explaining the various risk bias domains from Higgins (2021) is reproduced in Supporting Information: Appendix 2.

#### 4.3.4 Measures of treatment effect

We used a standardized mean difference effect size (Hedges' *g*) as the measure of treatment effect. The effect size was calculated in one of three ways:

- using the standardized mean difference approach in the metafor package (Viechtbauer, 2010) in R (R Core Team, 2019),
- using the standardized mean change in raw score approach in the metafor package in R,
- or using an “other” approach with Wilson's (Wilson, n.d.) Practical Meta‐Analysis Effect Size Calculator.

For studies using the posttest‐only with control group design, we used the escalc function (measure = “SMD”) of the metafor package in R to calculate the effect size and its unbiased estimate of the sampling variance (vtype = “UB”). For pretest–posttest designs with control groups and covariate adjustments, we used the same method described above using covariate‐adjusted means.

For pretest‐posttest designs with control groups that did not use covariate adjustment or for studies that used case‐control matching on a baseline measure of the outcome construct, we assumed the pretest standard deviation was an unbiased estimate of σ and used a raw mean change score approach as suggested in Morris (2002) (Equation 6). (The lack of pretest–posttest correlations in many studies was the primary reason we adopted Morris's raw mean change score approach.) Namely, we calculated the posttest–pretest mean differences for each group and used the pretest standard deviation of that group as the measure of variance. For designs with case‐control matching, we used the case‐control group standard deviation as the measure of variance. We then used the standardized mean change raw score parameter (measure = “SMCR”) in the escalc function of the metafor package of R to calculate the standardized mean difference effect size.

Finally, for a small number of studies where there was insufficient information to use the metafor package to calculate an effect size and its variance, we used Wilson's (Wilson, n.d.) Practical Meta‐Analysis Effect Size Calculator to calculate standardized mean difference effect sizes and their variances. The supplemental information provided in Randolph (2021) has notes on the calculation of effect sizes for these “other” effect size calculations and the R code that was used to calculate effect sizes.

#### 4.3.5 Unit of analysis issues

One of the main difficulties in synthesizing the Montessori literature was accounting for the multiple measures of an outcome's effect size within individual studies. To do this we used the cluster‐robust meta‐analytic approach suggested in Tanner‐Smith (2014), Tanner‐Smith (2016), and Tipton (2015). This allowed us to use every effect size while still accounting for the dependency of effect sizes within studies. Specific information on the cluster‐robust approach can be found in the Data synthesis section.

#### 4.3.6 Dealing with missing data

If outcome data were missing, we attempted to contact the study author(s) to get access to the missing data. If the author(s) never responded to our query, we excluded the study from the analysis. See the supplemental information (Randolph, 2021) for a record of which studies were excluded because of missing data and for documentation of attempts to contact study authors. In some cases, the authors sent the original data set and we used that data to generate the data missing in the study itself. A record of these instances is recorded in the data set and spreadsheet of included/excluded studies in the supplemental information as well.

#### 4.3.7 Assessment of heterogeneity

We used several methods to address study heterogeneity largely following the methods in Higgins (2021). For each outcome, we examined a forest plot containing the effect size estimate and its 95% confidence interval for each study and the weighted mean effect size and its 95% confidence interval. For outcomes with too many effect sizes to visualize in a forest plot with R software, we created a graphic display of study heterogeneity (i.e., a GOSH plot) (Olkin, 2012).

Although not included here, we also examined Baujat plots, radial plots, residual plots, and various other diagnostic and leverage tables included in the metafor package to identify studies with atypical heterogeneity. Funnel plots were examined for outcomes with 10 or more studies. We investigated kernel density plots of unweighted effect sizes to examine the distributional characteristics of the individual outcomes. In addition to the visual analysis of heterogeneity, we also examined several statistical measures of heterogeneity: the value of *Q,* its *df*, and its related *p* value, and the value of the I^2^ statistic. The R code and data set for these analyses can be found in Randolph (2021).

#### 4.3.8 Assessment of reporting biases

We used two methods to examine the presence and magnitude of publication bias: funnel plots and a trim and fill analysis,

First, as suggested in Higgins (2021), we assessed the degree of potential publication bias by visually analyzing funnel plots for outcomes with more than 10 studies and we did not carry out null‐hypothesis‐based statistical significance tests of publication bias.

Second, we used a trim and fill method as an additional tool to detect the presence of bias and to quantify the degree and direction of the bias. A clear explanation of the trim and fill method, from Murad (2018, p. 85) follows:

The trim and fill method is based on the funnel plot in which missing studies are imputed by creating a mirror image of opposite corresponding studies. The adjusted effect size accounting for the missing studies can be used as a sensitivity analysis to determine the presence and magnitude of publication bias. It is important to note that this adjusted effect size is based on strong assumptions about the missing studies and should only be used for the purpose of sensitivity analysis (i.e., should not be considered as a more accurate effect size to be used for decision making).

Specifically, we used the trimfill() function, which is based on the work of Duval (2000), in R's metafor package (Viechtbauer, 2010) using L0, R0, and Q0 parameters and using the Sidak‐Johnson method for random‐effect synthesis. A sensitivity analysis of those parameters, which is not reported here, clearly indicated that the bias was left‐sided. Duval (2000) recommends using either the L0 or RO estimate; for the sake of brevity, we only report the trim and fill results using the L0 parameter. The results did not differ substantively between L0 and R0.

As suggested in Shi (2019), we carefully considered how outlying studies might covary with publication bias results through a careful investigation of outlying studies as identified through funnel plots, Baujat plots, residual plots, leverage statistics, and forest plots. If a study was suspected of being an outlier, we reread the full text of the article to find insights into potential sources of heterogeneity; the results of the outlier investigation can be found threaded throughout the Results and Discussion sections.

Bias in the selection of the reported result was assessed using the RoB2 tool (Sterne, 2019) for studies with random assignment and the ROBBINS‐I tool (Sterne, 2016) for studies without randomized assignment.

#### 4.3.9 Data synthesis

For studies that reported multiple effect sizes for a single outcome, we used a cluster‐robust method for synthesizing effect sizes as described in Tanner‐Smith (2014), Tanner‐Smith (2016), and Tipton (2015) using the robumeta package (Fisher, 2017) in R. We used Tipton (2015)'s small‐sample correction and assumed the within‐study effect size correlation (*ρ*) to be 0.80. As suggested in Tipton (2015), we investigated this assumption with a sensitivity analysis using a range of values of ρ. We also estimated that dependencies between within‐study effect sizes were based more on correlations of effect sizes within studies than hierarchical effects, so we used “correlations” as model weights. The social studies outcome comprised effect sizes from only one study, so we used a random‐effects model there instead of a cluster–robust model. The complete R code for this analysis can be found in the supplemental information provided in Randolph (2021); see in particular the robust_main() function for details of the cluster–robust variance estimation and diagnostic methods.

For any outcomes with effect sizes from just one study (e.g., social studies) we used a random‐effects model (with REML estimator). See the random_main() function in the R code of Randolph (2021) for more details.

No data transformations were conducted and no missing data were imputed. (We excluded studies that had insufficient information to calculate an effect size and its variance.) We assessed model quality through an examination of residual plots, leverage charts, funnel plots, forest plots, radial plots, and Baujat plots; see the robust_main() and random_main() functions in the R code in Randolph (2021) for details.

#### 4.3.10 Subgroup analysis and investigation of heterogeneity

For aggregated academic and aggregated nonacademic outcomes, we conducted the following a priori subgroup analyses using cluster‐robust meta‐regression with the robumeta package (Fisher, 2017) using the methods described in Tanner‐Smith (2014). See the corresponding functions in the R code written for this meta‐analysis (Randolph, 2021) for more information; the corresponding functions are listed in italics below.

- Duration of follow‐up (in years); *mod_followup()*
- Treatment duration (in weeks); *mod_treatment()*
- Intervention setting (i.e., private or public Montessori); *mod_setting()*
- Student's grade level (preschool, elementary, middle school, or high school); *mod_level()*
- Assignment (random vs. nonrandom); *random_method()*

For the two continuous moderators (duration of follow‐up and treatment duration), we first performed centering and estimated both between‐study and within‐study effect size estimates. We did not conduct a subgroup analysis for each outcome individually because of the sample size requirements for cluster‐robust meta‐regression. For multinomial outcomes, we conducted an omnibus test of statistical significance as suggested in Tanner‐Smith (2014).

The social studies outcome only had one study with multiple outcomes so a random‐effects model was used. See the R code in the supplement (Randolph, 2021) for more details.

As mentioned in the protocol, we extracted information on student demographic characteristics, such as race/ethnicity, at‐risk status, gifted/talented, or measures of socioeconomic status, but we did not intend to examine these characteristics as moderators in this review. We intended to use these demographic data to richly characterize study participants, to examine whether these variables were used as covariates in study analyses, and to facilitate follow‐up reviews that might examine demographic characteristics as moderators.

#### 4.3.11 Sensitivity analysis

We conducted the following sensitivity analysis. See the related R functions in the supplemental information (Randolph, 2021) for more information; the function names are given in italics below.

- We compared results between cluster‐robust, random‐effects, and fixed‐effects models; *es_calc_method()*
- We examined how ρ (i.e., the correlation of within‐study effect sizes) covaried with effect sizes; *robust_main()*
- We conducted a leave‐one‐out analysis using the leave1out function in metafor the package; *robust_main()*

#### 4.3.12 Summary of findings and assessment of the certainty of the evidence

We used the GRADE approach described in Higgins (2021) to assess the certainty of evidence and summarize findings. The GRADE approach results in one of four ordinal ratings of the certainty of evidence in an outcome: *high, moderate, low*, or *very low*.

The first step in the GRADE approach is to establish an initial level of certainty. Randomized studies or studies evaluated using the ROBINS‐I tool (Sterne, 2016) for examining risk of bias in nonrandomized studies are given an initial certainty of *high certainty*. Observational studies not using the ROBINS‐I tool are given an initial certainty rating of *low certainty*.

Next, review authors downgrade or upgrade the quality of evidence based on several GRADE factors. They downgrade the certainty of evidence based on *risk of bias*, *inconsistency*, *indirectness*, *imprecision*, and *publication bias*. Review authors can also upgrade evidence based on a *large effect size*, *evidence of a dose response*, or if there are *plausible confounding factors* that may work together to underestimate a treatment effect. (More detailed information on those factors causing upgrades or downgrades can be found later in this section). The final step is to establish a final level of certainty into one of the four categories mentioned above.

In terms of the number of levels to downgrade or upgrade, we used this guidance from Higgins (2021):

The highest certainty rating is a body of evidence where there are no concerns in any of the GRADE factors…. Review authors often downgrade evidence to moderate, low, or even very low certainty evidence, depending on the presence of the five [GRADE] factors. Usually, certainty ratings will fall by one level for each factor, up to a maximum of three levels for all factors. If there are severe problems for one domain…., evidence may fall by two levels due to that factor alone… Review authors will generally grade evidence from sound nonrandomized studies as low certainty, even if ROBINS‐I is used. If, however, such studies yield large effects and there is obvious bias explaining those effects, review authors may rate the evidence as moderate or‐‐if the effect is large enough‐‐even as high certainty. (p. 391)

The first author independently assessed the certainty of evidence and other authors reviewed those certainty assessments. Any disagreements were resolved through consensus. The fine details of the GRADE approach used in this review are explained below.

All included studies initially were assumed to have an initial level of “high certainty” because they were all randomized trials or were evaluated with the ROBINS‐1. We then downgraded or upgraded the level of certainty based on the various GRADE factors.

First, we considered risk of bias. We assumed all studies to have low risk of bias. We then downgraded the certainty of evidence by one or two levels based on the RoB 2 (Sterne, 2019) for studies using random assignment and the ROBINS‐I tool (Sterne, 2016) for studies not using random assignment. We used the following criteria for downgrading studies based on their risk of bias:

A rating of high certainty evidence can be achieved only when most evidence come from studies that meet the criteria for low risk of bias. The certainty of evidence might be downgraded by one level when most of the evidence comes from individual studies either with a crucial limitation for one item, or with some limitations for multiple items. (Higgins, 2021, p. 392)

Furthermore, it was possible to downgrade two levels when there were very serious limitations defined as a “crucial limitation for one or more criteria sufficient to substantially lower their confidence of an effect” (Higgins, 2021, p. 393). See the Assessment of risk of bias in included studies section for more details on risk of bias.

After downgrading for risk of bias, we then downgraded for other GRADE factors: inconsistency, indirectness, imprecision, or publication bias and upgraded for large effects, dose–response effects, or opposing residual bias and confounding as described in Higgins (2021).

We downgraded for inconsistency if an outcome met Higgins' (2021, p. 259) definition of *considerable heterogeneity: I* ^2^ values equal to or above 75%.

We downgraded for indirectness when there were indirect comparisons or that could cause “a restricted version of the main review question in terms of population, intervention, comparator, or outcome” as described in Higgins (2021, pp. 393–394).

We downgraded for imprecision up to two levels if an outcome did not meet each of the three following criteria:

- The number of participants for that outcome was below the optimal information size. We calculated the optimal information size as the sample size needed for a study given the oft‐used Cohen (1988) convention for a “small” effect size (i.e., a standardized mean difference effect size of 0.20), one predictor, *α* = 0.05, *β* = 0.80, and a two‐sided test. In this case, the optimal information size was 387 as suggested in a sample size table from Randolph (2019).
- The 95% confidence intervals for the standardized mean difference for the outcome included 0.00. We downgraded up to two levels if the 95% CI included small positive effects and small negative effects.
- The *df* resulting from a cluster‐robust analysis was less than 4.00 (Tanner‐Smith, 2014; Tanner‐Smith, 2016; Tipton, 2015).

For outcomes with more than 10 studies, we downgraded for publication bias if the funnel plots showed marked asymmetry.

We upgraded for large effects if the standardized mean difference effect size was greater than 0.80 in absolute value, which corresponds with Cohen (1988)'s convention for a “large” effect size in laboratory studies in the behavioral sciences.

We upgraded if there was evidence of a dose–response effect (i.e., if there was evidence from a meta‐regression that treatment duration had a positive correlation with the effect size).

Finally, we upgraded for opposing residual bias and confounding if we found strong evidence that “all plausible biases from randomized or nonrandomized studies may be working to underestimate an apparent intervention” (Higgins, 2021, p. 397).

When assigning textual descriptions of the magnitude of effect sizes, we used the conventions of Cohen (1988) such that standardized mean difference effect sizes of 0.20 are *small*, 0.50 are *medium*, and 0.80 or greater are *large*.

## 5 RESULTS

### 5.1 Description of studies

#### 5.1.1 Results of the search

**Figure 1:**
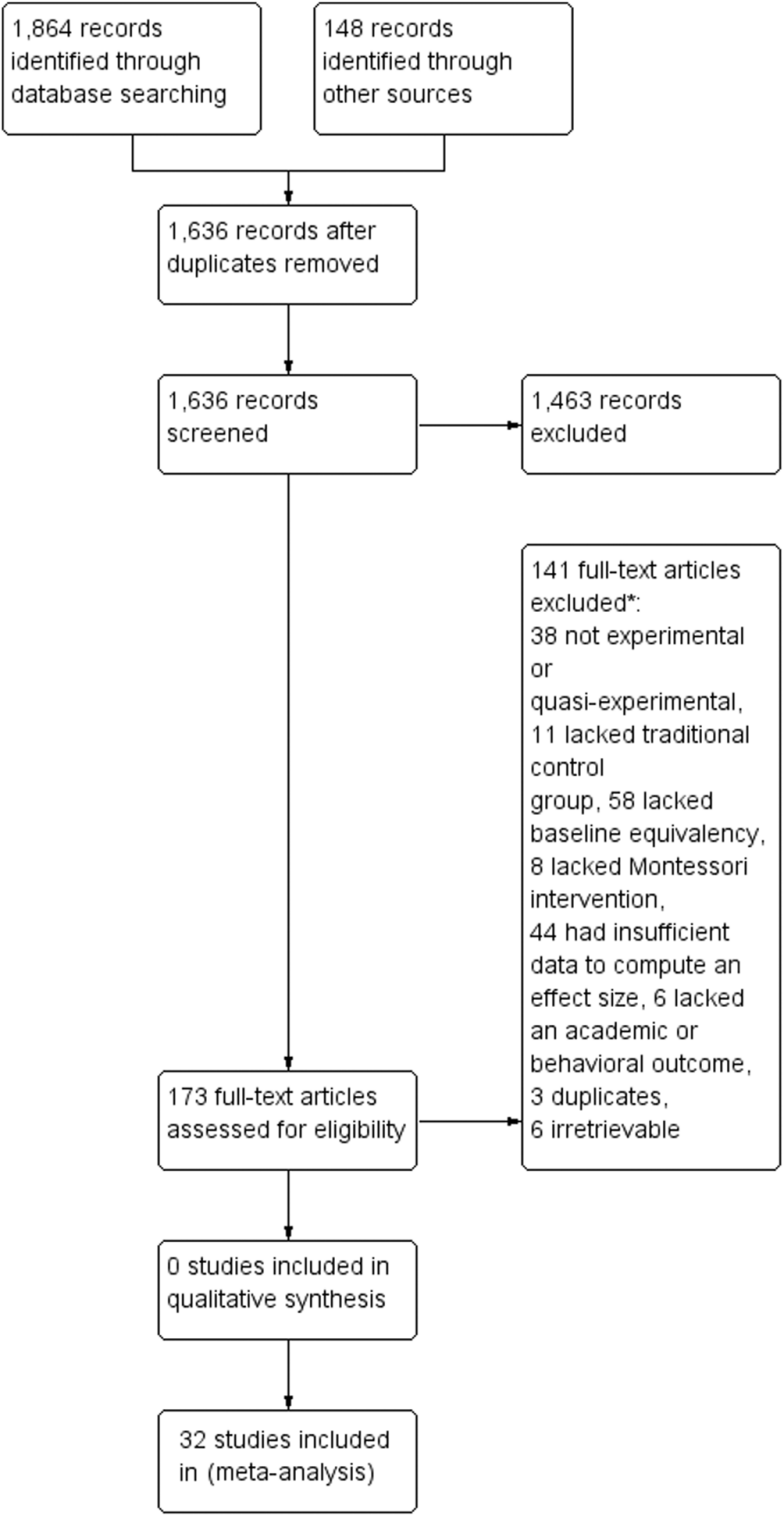
Flow diagram of the literature search for studies included in meta‐analysis. *Studies could have been excluded for more than one reason so the sum of exclusion reasons does not equal 141.

The systematic search including the initial 2014 and the follow‐up 2020 search yielded 2,012 records from all sources. As shown in the flow diagram in Figure 1, after the removal of duplicates, abstract screening, and full‐text screening, 32 studies met the criteria for inclusion and were included in this meta‐analysis.

The PRISMA flow diagram combines results from the initial and follow‐up searches of online academic databases (*n* = 1864). Also displayed are records retrieved from other sources, including gray literature websites, Montessori professional association websites, hand‐searched journals, reference searches, expert referrals, and open web search engines (*n* = 148). After duplicate removal, 1,636 results were screened at the title and abstract level, excluding 1463 records based on inclusion criteria. The full texts of the remaining articles (*n* = 173) were screened and 141 were excluded, leaving 32 studies included for quantitative synthesis. Bibliographic data for retrieved studies were managed using Zotero bibliographic software. A complete list of the included and excluded studies, along with detailed information on why each study was excluded is provided in the supplemental information in Randolph (2021).

#### 5.1.2 Included studies

Table 1 summarizes the studies and key characteristics of the 32 included studies. It includes each study's in‐text citation, a brief description of the participants including country (the default is the United States because over half the studies were conducted there), the study design, a brief description of the intervention, the outcomes used in the meta‐analysis, the dates when the study was done if noted in the article, and the funding source. No study declared a conflict of interest.

Table 3 provides information, in the aggregate, on the characteristics of studies that contributed at least one academic or nonacademic effect size. A summary of those study characteristics is provided in the lists below:

**Table 3:** Frequencies and percentages of effect sizes by study characteristics.

Study characteristic	Academic effect sizes (%)	Nonacademic effect sizes (%)
Experimental design		
Posttest‐only with control group	20/113 (17.7)	20/91 (22.0)
Pretest‐posttest with control group	79/113 (69.9)	58/91 (63.7)
Matched pairs	14/113 (12.4)	39/91 (42.9)
Rationale for baseline equivalency		
Nonstatistically significant pretest differences	58/113 (51.3)	55/91 (60.4)
Gain scores	30/113 (26.5)	46/91 (50.5)
Covariate‐adjusted means	57/113 (50.4)	32/91 (35.2)
Random assignment	72/113 (63.7)	15/91 (16.5)
Follow‐up at least 1 year after intervention	37/113 (32.7)	13/91 (14.3)
Public Montessori school	76/107 (71.0)	36/70 (51.4)
Public control‐group school	60/108 (55.6)	36/70 (51.4)
Standardized test used as measure	71/113 (62.8)	0/91 (0.0)
Grade level		
Preschool or kindergarten	36/113 (31.9)	39/91 (42.9)
Elementary school	44/113 (38.9)	44/91 (48.4)
Middle school	19/113 (16.8)	8/91 (8.8)
High school	14/113 (12.4)	0/91 (0.9)
Geographic region		
Asia	5/113 (4.4)	5/91 (5.5)
Europe	8/113 (7.1)	40/91 (44.0)
Middle East	1/113 (0.9)	0/91 (0.0)
North America	99/113 (87.6)	46/91 (50.5)
Peer‐reviewed publication	81/113 (71.7)	78/13 (85.7)

##### Studies with academic outcomes

- Studies with academic outcomes tended to use the pretest‐posttest with control group design (Shadish 2002). (Studies that used pretest‐posttest without control group designs were excluded).
- The majority of effect sizes of academic outcomes came from studies that used random assignment as evidence of baseline equivalency. Other common sources of evidence of baseline equivalency were nonstatistically significant differences on a pretest or using the pretest as a statistical covariate.
- Most studies with academic outcomes collected their data within one year of completion of the intervention.
- In terms of setting, most studies with academic outcomes were conducted in public settings for both the traditional and Montessori conditions.
- Standardized measures of academic achievement were the most frequently used type of measure in studies with academic outcomes.
- Studies with academic outcomes tended to be conducted in elementary or pre‐K settings.
- The vast majority of studies with academic outcomes were conducted in North America.
- Most studies with academic outcomes were published in peer‐reviewed publications.

##### Studies with nonacademic outcomes

- Similar to studies with academic outcomes, nonacademic studies most frequently used the pretest‐posttest with control group design.
- In contrast to studies with academic outcomes, studies with nonacademic outcomes tended to use nonstatistically significant pretest measures and/or gain scores as evidence of baseline equivalency. Studies with nonacademic outcomes tended not to use random assignment.
- Like studies with academic outcomes, studies with nonacademic outcomes tended to collect data within one year of completion of the intervention.
- Studies with nonacademic outcomes were conducted in an approximately equal proportion of public and private settings.
- As expected, studies with nonacademic outcomes did not use standardized tests of achievement.
- Similar to studies with academic outcomes, the most frequently used settings were in elementary and pre‐K.
- Most studies with nonacademic outcomes had first authors from North America or Europe.
- Most studies with nonacademic outcomes were published in peer‐reviewed forums.

The list below provides a link to each of the 32 included studies. The supplemental information in Randolph 2021 contains a data set where we extracted the information specified in the coding book; specific details on each study can be found there.

1. Alburaidi 2019
2. Ansari 2014
3. Aydoğan 2016
4. Besançon 2013
5. Coyle 1968
6. Culclasure 2018
7. Denervaud 2019
8. Denervaud 2020
9. Doğru 2015
10. Elben 2015
11. Faryadi 2017
12. Fleege 1967
13. Galindo 2014
14. Hoseinpoor 2014
15. Jones 1979
16. Juanga 2015
17. Kayili 2016b
18. Kayili 2016a
19. Kirkham 2017
20. Lillard 2006
21. Lillard 2012
22. Lillard 2017
23. Mallett 2015
24. Manner 1999
25. Miller 1983
26. Miller 1984
27. Mix 2017
28. Prendergast 1969
29. Rathunde 2005a
30. Rathunde 2005b
31. Tobin 2015
32. Yussen 1980

#### 5.1.3 Excluded studies

Of the 173 studies that underwent full‐text screening, 141 studies were excluded. See the Excluded studies section for references to the 141 excluded studies. The following list summarizes how many studies were excluded based on each exclusion criterion:

- a lack of proof of equivalency of Montessori and traditional groups at baseline (*n* = 58),
- did not use experimental or quasi‐experimental research design (*n* = 38),
- insufficient information to calculate an effect size (*n* = 44),
- a lack of a Montessori‐based intervention (*n* = 8),
- the absence of a traditional, control group (*n* = 11),
- a lack of an academic or behavioral outcome (*n =* 6*)*,
- was a duplicate study (*n* = 3),
- or was irretrievable (*n =* 6).

Note that exclusion criteria were not mutually exclusive, so a study could have been excluded for one or more reasons. If a study met at least one exclusion criterion, the other exclusion criteria may not have been assessed. An online data set in the supplement (Randolph, 2021) to this review has a list of each article considered for inclusion, which inclusion criteria were met by each study, and notes on selected studies. Six studies were irretrievable as shown in the sheet labeled as *irretrievable* in the included/excluded studies data set in the supplemental online information.

### 5.2 Risk of bias in included studies

**Figure 2:**
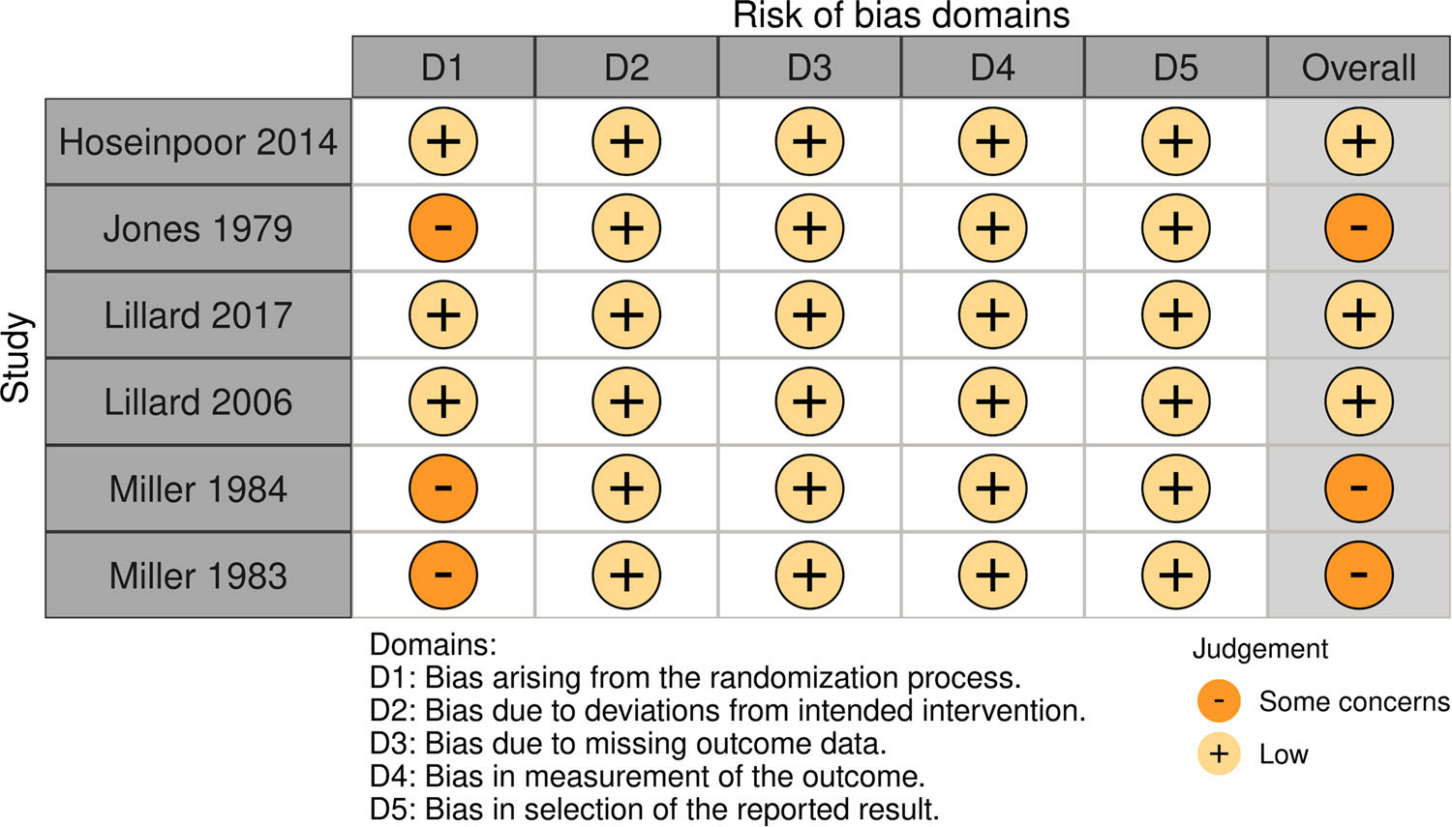
Risk of bias for studies with random assignment.

**Figure 3:**
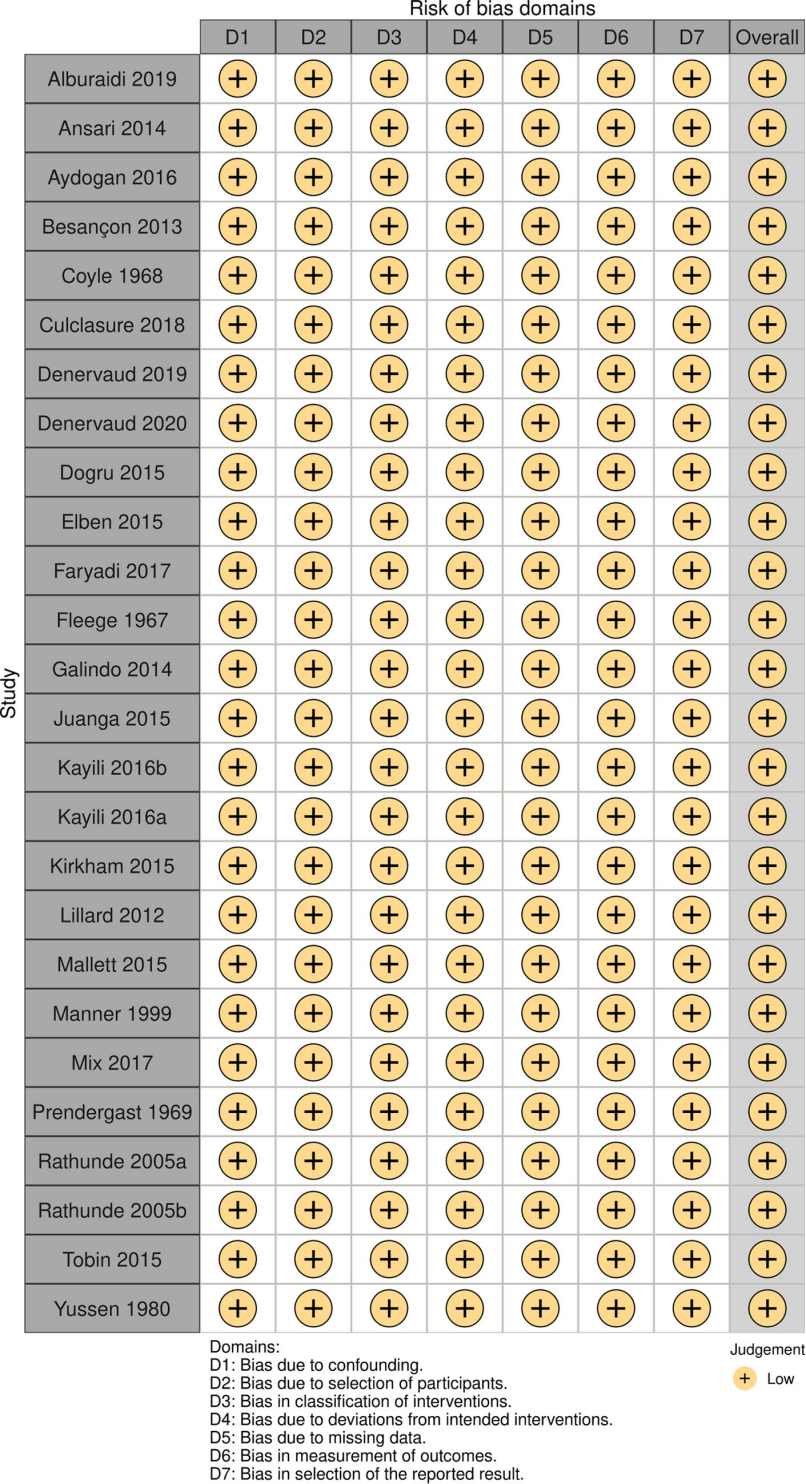
Risk of bias for studies with nonrandomized assignment.

Overall, the risk of bias for the six randomized studies (Figure 2) was considered to be low. Similarly, the risk of bias for 26 nonrandomized studies was low (Figure 3). Although it is typical for nonrandomized studies to have overall risk‐of‐bias ratings of *some concerns* or *high* risk of bias, we believe that our nonrandomized studies typically were at *low risk* of bias because of the strict inclusion criteria we set. For example, nonrandomized studies were excluded if there was not strong evidence for baseline equivalency, which addresses the domains of confounding and selection of participants in the Robbins‐I tool (Sterne, 2016).

#### 5.2.1 Allocation (selection bias)

Selection bias was deemed to be low in the 32 included studies. For randomized studies, three of the six studies were rated as having low risk for the randomization process; the exceptions were Jones (1979), Miller (1983), and Miller (1984), which were rated as having “some concerns.” For the nonrandomized studies, all 26 studies were deemed to have low risk in terms of confounding and selection of participants. Documentation on these decisions can be found in the online supplemental information (Randolph, 2021).

#### 5.2.2 Blinding (performance bias and detection bias)

Because of the nature of the intervention, it was not possible to blind participants to whether they were receiving Montessori or traditional education. Therefore, we did not assess the risk of bias in this domain.

#### 5.2.3 Incomplete outcome data (attrition bias)

All 32 included studies were rated as low risk in terms of attrition bias.

#### 5.2.4 Selective reporting (reporting bias)

All 32 included studies were rated as low risk in terms of selective reporting bias.

#### 5.2.5 Other potential sources of bias

Information on other potential sources of bias not listed above can be found in Figures 2 and 3. In short, we deemed there to be low risk of bias from other potential sources.

### 5.3 Effects of interventions

#### 5.3.1 Academic outcomes

##### Main effects for academic outcomes

**Figure 4:**
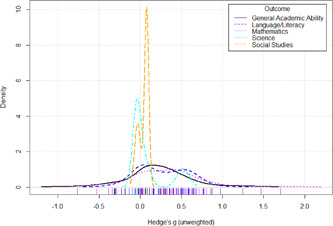
Kernel density plot of unweighted values of Hedges’ g for individual academic outcomes.

Table 4 and Figure 4 (a histogram of raw effect sizes for academic outcomes) summarize the main effects of Montessori education versus traditional education for academic outcomes. For readers unfamiliar with the interpretation of meta‐analytic main‐effects tables, we describe the interpretation of each column in Table 4 here before discussing the specific results in the following paragraphs. The first column in Table 4 indicates the outcome. The second column indicates the standardized mean difference effect size, Hedges' *g*, which is a sample‐size corrected version of Cohen's *d*. Positive effect sizes favor Montessori education over traditional education, effect sizes of zero indicate the equality of Montessori and traditional education, and negative effect sizes favor traditional education over Montessori education. Since all of the effect sizes in Table 4 are positive, one can interpret the effect size as the number of standard deviations that Montessori students on average performed better than traditional students performed. See (Cohen, 1988; Kraft, 2020) for additional resources for interpreting the magnitude of an effect size. The third column indicates the 95% confidence intervals for the effect size, which can be interpreted as the plausible range of the population effect size, given chance (Higgins, 2021). The fourth column, *p*, is the two‐tailed probability of the effect size's being equal to 0.00 given chance; a Hedges' *g* effect size of 0.00 (the null value) would indicate that Montessori education and traditional education are equal. Values of *p* below 0.05 are typically regarded as *statistically significant*. The fourth column indicates the *df* (degrees of freedom) when a cluster‐robust model was used. Tanner‐Smith (2014) cautioned that cluster–robust results should not be interpreted as being reliable when the *df* is less than 4.00. The fifth column, *I* ^2^, is a measure of study heterogeneity–the difference in effect sizes among studies–expressed as a percentage. Higgins (2021, p. 259) gives the following rules of thumb for interpreting *I* ^2^:

- 0%–40%: might not be important;
- 30%–60%: may represent moderate heterogeneity;
- 50%–90%: may represent substantial heterogeneity;
- 75%–100%: considerable heterogeneity.

**Table 4:** Main effects of Montessori education versus traditional education academic outcomes.

Outcome	Hedges’ *g*	95% CI of *g*	*df*	*p*	*I* ^*2*^	*N* of studies	*N* of effect sizes
All academic outcomes	0.24	0.13, 0.36	17.20	0.000	76.54	24	113
General academic ability	0.26	0.06, 0.46	7.03	0.018	85.03	9	24
Language/literacy	0.17	0.03, 0.31	7.94	0.022	71.61	16	45
Mathematics	0.22	0.06, 0.39	7.31	0.015	64.58	12	36
Science	0.15	−0.61, 0.90	1.95	0.480	82.48	3	5
Social studies^a^	0.05	−0.02, 0.12	–	0.187	83.67	1	3

*Note*: Cluster robust estimates with *df* < 4.00 should be interpreted with caution (Tanner‐Smith, Tipton, & Polanin, 2016). Positive effect sizes favor Montessori over traditional education.

Abbreviation: CI, confidence interval.

^a^ A cluster‐robust model was used for all outcomes except social studies, in which a random‐effects model was used. The social studies outcome had effect sizes from only one study.

Finally, the fifth and sixth columns of Table 4 indicate the number of studies and the number of effect sizes, respectively, that contributed to each outcome.

As Table 4 shows, with all academic outcomes combined across 24 studies and 113 effect sizes, Montessori students on average performed 0.24 standard deviations higher than students in traditional education programs, 95% CI [0.13, 0.36], with high study heterogeneity, *I* ^2^ = 76.54%.

**Figure 5:**
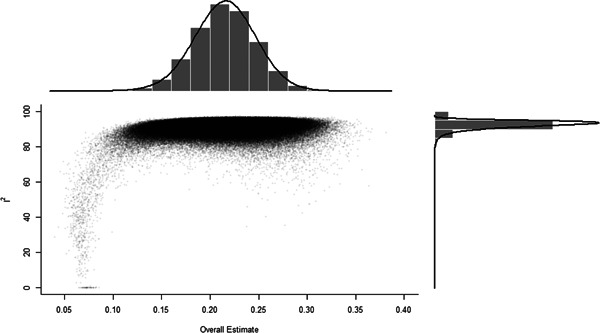
Graphical display of study heterogeneity for all academic outcomes combined. In the GOSH plot above, the *X*‐axis (Overall Estimate) shows the Hedges’ *g* effect size. Positive effect sizes favor Montessori education over traditional education. The Y‐axis represents a measure of study heterogeneity, *I* ^2^, where higher values represent more study heterogeneity. The black data points are simulated values of the population effect size and heterogeneity.

See Figure 5 for a graphical display of study heterogeneity (i.e., a GOSH plot) and effect sizes of academic outcomes from a bootstrap analysis of 1,000,000 samples of academic effect sizes. That GOSH plot shows the results of plotting the effect size and heterogeneity of random subsets of studies. The measure of heterogeneity (*I* ^2^) is plotted on the vertical axis and the measure of effect size is plotted on the horizontal axis. So, each data point on the GOSH plot represents the heterogeneity and summary effect size of a random sample of studies. The darker the area, the more likely it is that that is the range in which the population effect size resides. (The population effect size is the unknowable effect of Montessori education that generalizes to all Montessori programs, not just the ones investigated in the studies included here). In Figure 5, the darkest area varies horizontally between approximately 0.10 and 0.32 in effect size, so, this is the plausible range of the effect of Montessori academic outcomes. This dark area is positioned vertically at around an *I* ^2^ value of 90%, indicating high study heterogeneity. On the left‐hand side of Figure 5, there is a small, light tail extending down to 0% heterogeneity and with an effect size of 0.07, meaning that there is a small statistical probability that the population summary effect size is actually 0.07 with very low heterogeneity. See Olkin (2012) for more information on the interpretation of the GOSH plot.

We believe there is moderate quality of evidence for this finding as discussed in the Summary of Findings table. (Note that all of our ratings of quality of evidence are based on the GRADE system for rating quality of evidence; see Higgins, 2021).

**Figure 6:**
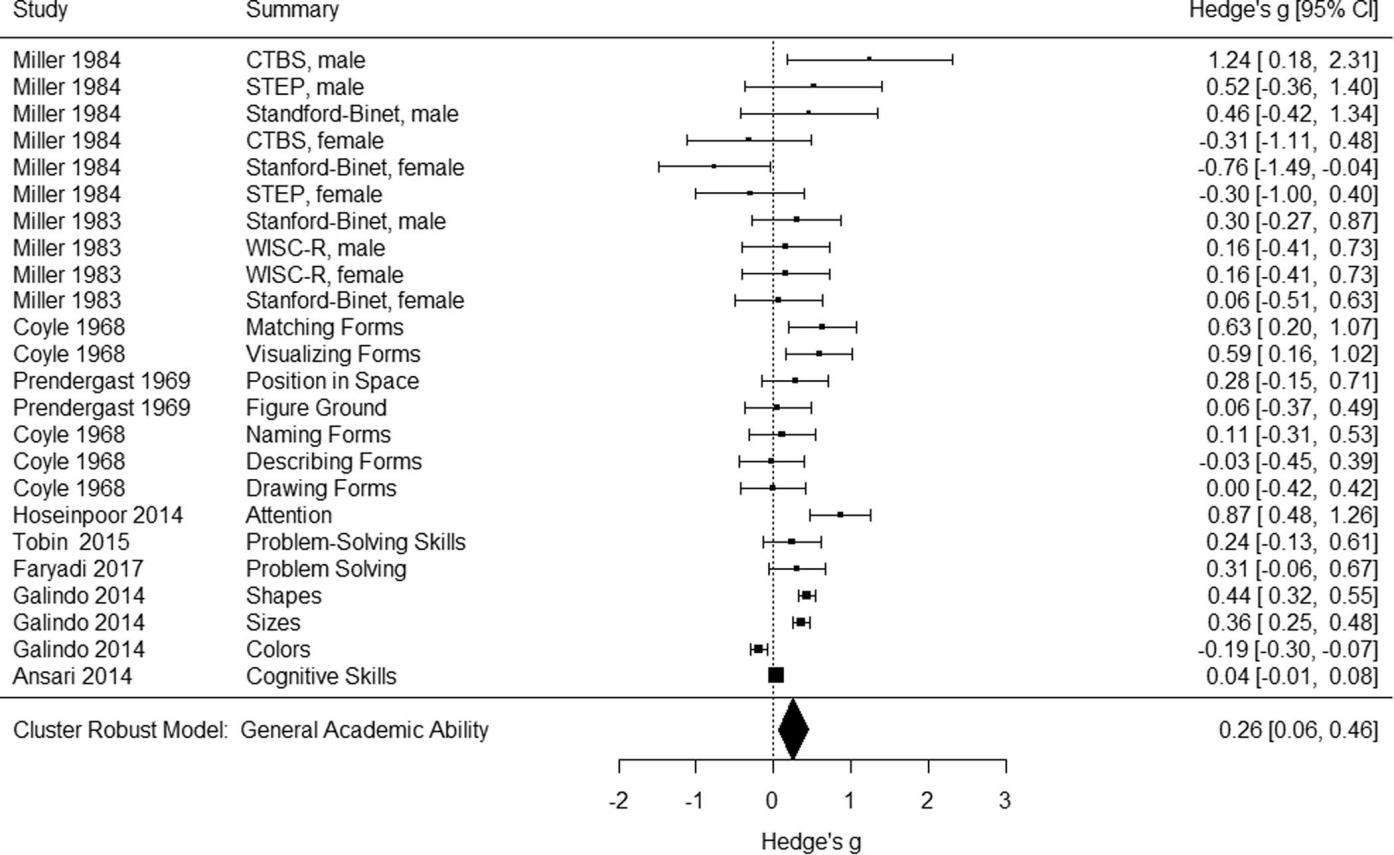
Forest plot for general academic ability.

**Figure 7:**
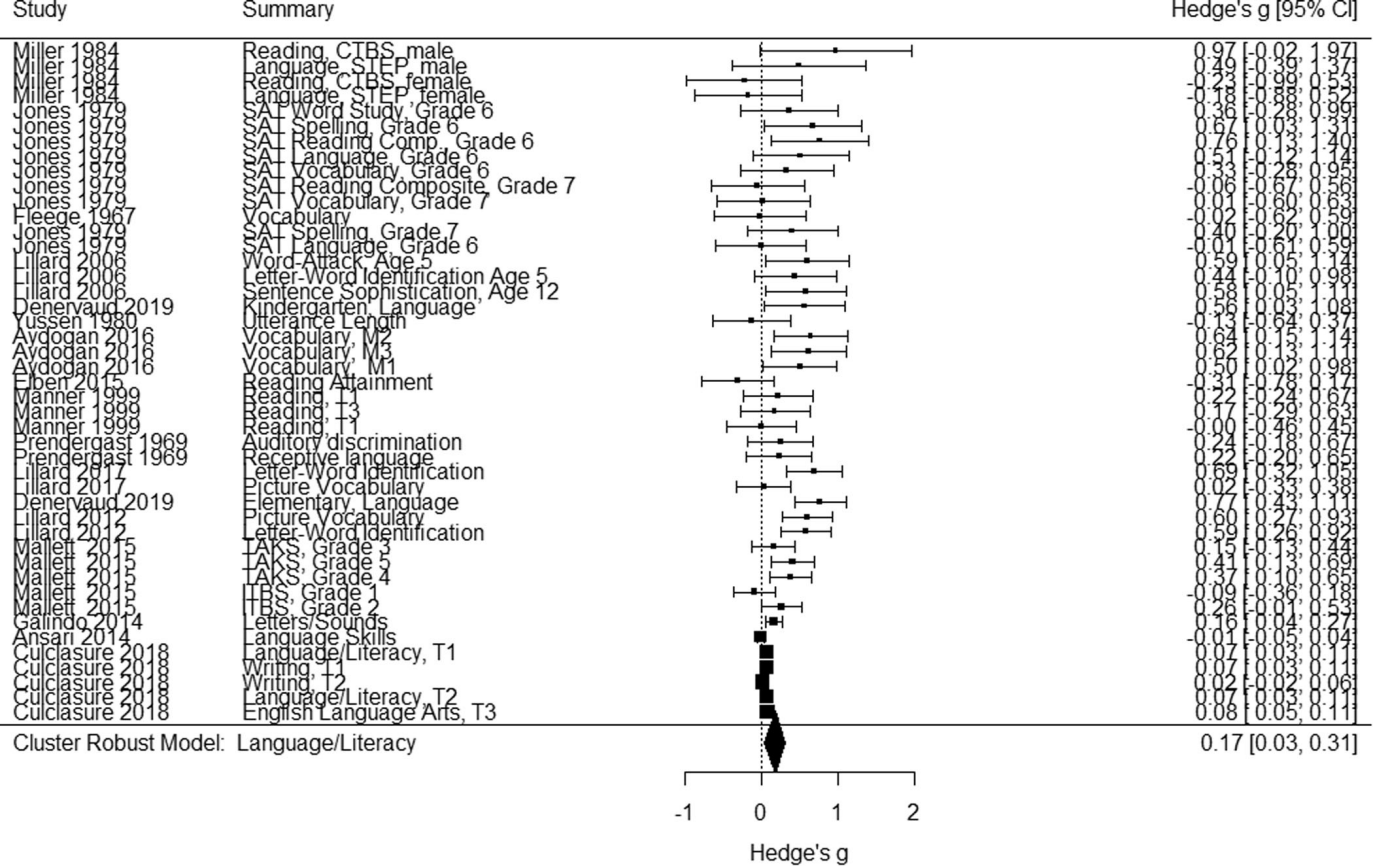
Forest plot for language/literacy. Positive effect sizes favor Montessori education over traditional education.

**Figure 8:**
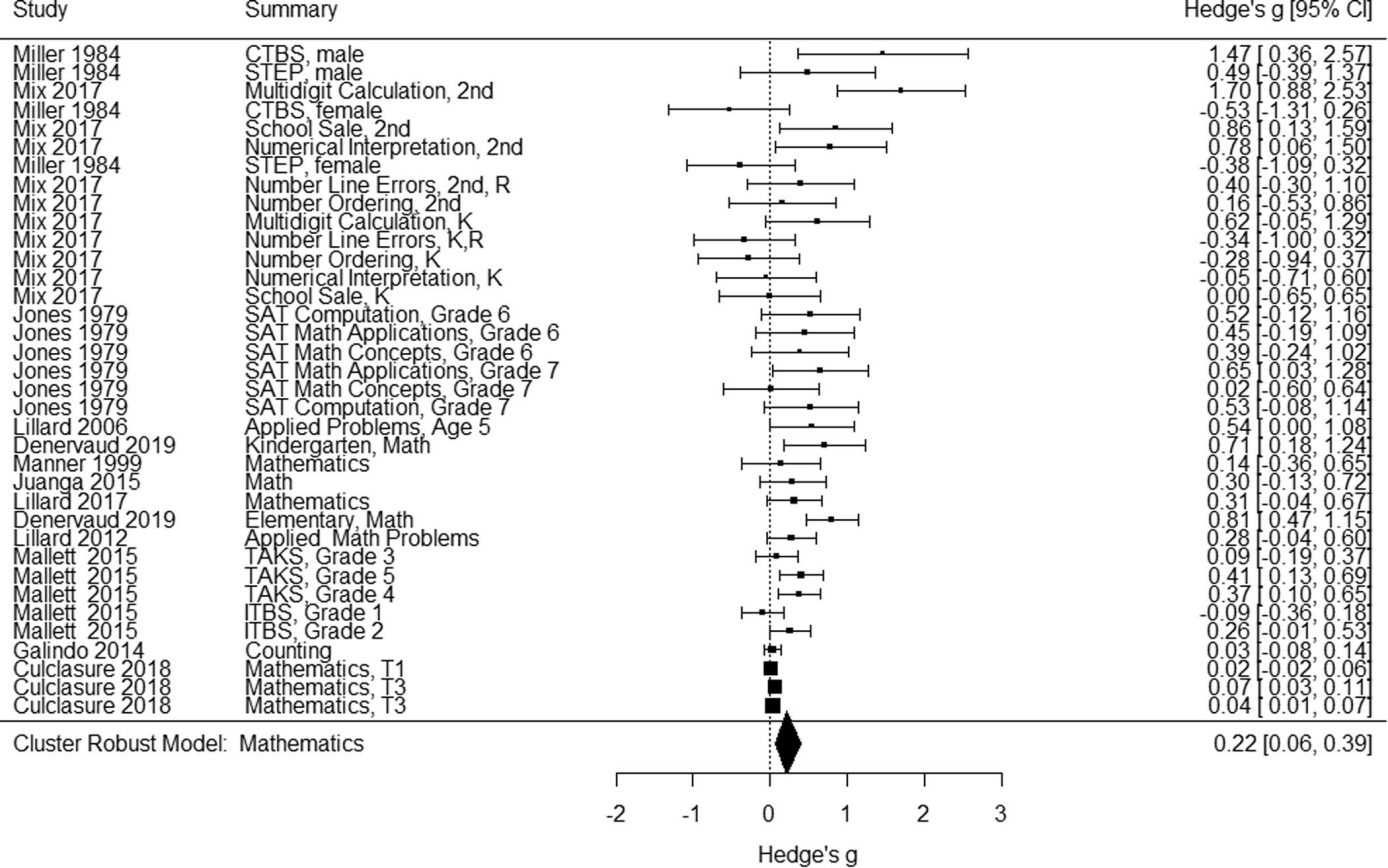
Forest plot for mathematics. Positive effect sizes favor Montessori education over traditional education.

**Figure 9:**
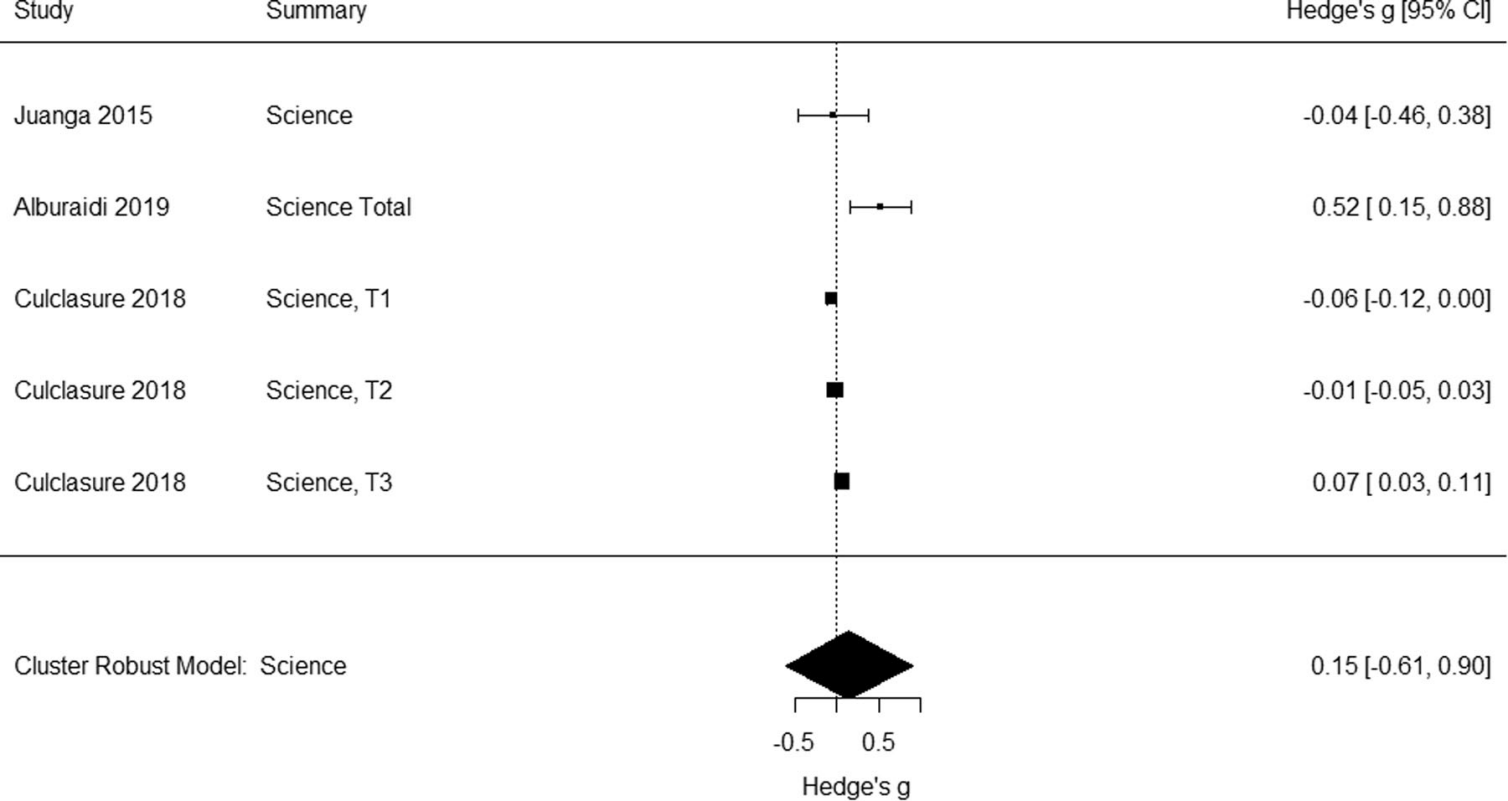
Forest plot for science. Positive outcomes favor Montessori education over traditional education.

**Figure 10:**
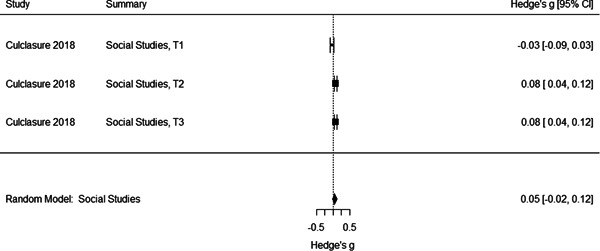
Forest plot for social studies. Positive effect sizes favor Montessori education over traditional education.

In terms of the individual academic outcomes presented in Table 4, all academic outcomes were in favor of Montessori education. In summary, there was moderate quality of evidence that Montessori education outperformed traditional education in terms of general academic ability (Figure 6) (Hedges' *g* = 0.26, 95% CI [0.06, 0.46]), high quality of evidence for language/literacy (Figure 7) (Hedges' *g* = 0.17, 95% CI [0.03, 0.31]), high quality of evidence for mathematics (Figure 8) (Hedges' *g* = 0.22, 95% CI [0.06, 0.39]), low quality of evidence for science (Figure 9) (Hedges' *g* = 0.15, 95% CI [−0.61, 0.46]), and moderate quality of evidence for social studies (Figure 10) (Hedges' *g* = 0.05, 95% CI [−0.01, 0.12]). Note that the three social studies outcomes only come from Culclasure (2018). See the Summary of findings Table 1 for more information on the certainty of evidence. Note that judgments of the quality of evidence throughout this review are based on the GRADE system; see the Quality of the evidence section for details.

##### Moderator analysis for academic outcomes

###### Random assignment

A moderator analysis investigates the degree to which the relation between two outcome variables is influenced by a different study variable. A sensitivity analysis investigates the degree to which methodological or analytical decisions are differentially associated with effect sizes (Higgins, 2021). In the following section, we report the results of a moderator analysis for all academic outcomes combined for the following attributes: random vs. nonrandom assignment, grade level, Montessori setting (public vs. private), treatment duration, and length of follow up. We were unable to reliably conduct a moderator and sensitivity analysis for each individual outcome because there was an insufficient number of effect sizes to do so.

Moderator analyses are typically conducted via a technique called meta‐regression (Higgins, 2021). An example of a meta‐regression results table comparing the effect sizes of studies with nonrandom assignment to random assignment is presented in Table 5. For readers who lack familiarity with results from cluster‐robust meta‐regression, we provide some guidance in the paragraphs below.

**Table 5:** Cluster robust meta‐regression for random assignment for academic effect sizes.

Effect (*n* of effect sizes)	Hedges’ *g*	95% CI of *g*	*df*	*p*
Nonrandom assignment (72)^a^	0.19	0.07, 0.31	12.06	0.004
Random assignment (41)	0.28	−0.07, 0.63	5.76	0.095

*Note*: *N* of studies = 24, *N* of effect sizes = 113, assumed *ρ* = 0.8, *I* ^2^ = 76.90. Cluster robust estimates with *df* < 4.00 should be interpreted with caution (Tanner‐Smith, Tipton, & Polanin, 2016). Positive effect sizes favor Montessori over traditional education.

Abbreviation: CI, confidence interval.

^a^ Reference category/intercept.

The first column of Table 5 shows the various levels or categories of the attribute that are being investigated and the number of effect sizes included for that category. Here we are investigating the attribute of assignment (i.e., random vs. nonrandom assignment). There were 72 effect sizes from studies with nonrandom assignment and 41 effect sizes from studies with random assignment. The row category denoted with a superscript a is the reference category.

The regression coefficient in the second column of Table 5 is the standardized mean difference effect size (i.e., Hedges' *g*). In this case, the effect size for the nonrandom assignment category was 0.19, which means that, when considering only effect sizes from studies with nonrandom assignment, Montessori students on average performed 0.19 standard deviations higher than traditional education students. The effect size coefficient (Hedges' *g*) for any row category besides the reference category can be interpreted as the mean effect size difference between that row category and the reference category. In this case, the effect size coefficient for random assignment is 0.28, meaning that the average effect size was 0.28 units higher in randomized studies than in nonrandomized studies. One can find the effect size for any row category besides the reference category by adding the coefficient from the reference category to the coefficient from the row category. In this case, the estimated effect size for randomized studies was 0.47, since 0.19 + 0.28 = 0.47.

The third column in Table 5 shows the 95% confidence interval for the effect size coefficient. Put simply, the 95% confidence intervals show the plausible values of the mean effect size in the entire population. See Higgins (2021) for a more detailed explanation of the interpretation of confidence intervals around a meta‐analytic point estimate.

The fourth column in Table 5 (*df*) shows the degrees of freedom for each coefficient. According to Tipton 2015, coefficients with a *df* less than 4 may not be reliable and should be interpreted with caution. Finally, the last column, *p*, gives the probability that the population parameter for the effect size coefficient in the second column might be zero, given chance. In short, Hedges' *g* for the reference category, which will always be the first‐row category in the meta‐regression tables presented here, is the effect size for that reference category. The value in the Hedges' *g* for any category besides the reference category is the mean effect size difference between that category and the reference category.

Finally, in terms of the substantive interpretation of Table 5, there are three points of interest. Both randomized and nonrandomized studies had positive academic effect sizes in favor of Montessori education versus traditional education. Randomly assigned studies had significantly larger academic effect sizes than nonrandomized studies; however, the 95% CI [−0.07, 0.63] indicated some statistical uncertainty regarding the degree to which one could accurately generalize these results to the entire population of students.

###### Grade level

Table 6 shows the meta‐regression results for academic outcomes when disaggregated by grade level. Montessori education outperformed traditional education in all grade levels. The greatest academic effects of Montessori education were found at the elementary level (Hedges' *g* = 0.36). The mean preschool, middle school, and high school academic effect sizes were 0.16, 0.09, and 0.15 smaller, respectively, than the effect sizes for elementary school. However, all of these mean effect size differences had a high degree of imprecision as evidenced by their wide 95% CIs. A Wald test–an omnibus test for detecting statistically significant differences among means—yielded a *p* value of 0.761, which indicates that the differences in the effect sizes among grade levels would have been likely given chance. The middle school statistical results had a low *df* indicating that this moderator effect should be interpreted with caution. In summary, Montessori education had greater academic effects than traditional education at all grade levels, with the strongest effect being at the elementary level; however, there is much statistical uncertainty regarding this result.

**Table 6:** Cluster robust meta‐regression for grade level for academic effect sizes.

Effect (*n* of effect sizes)	Hedges’ *g*	95% CI of *g*	*df*	*p*
Elementary school^a^ (36)	0.36	(0.09, 0.62)	6.41	0.017
Preschool (44)	−0.16	(−0.43, 0.11)	13.44	0.233
Middle school (19)	−0.09	(−1.14, 0.96)	1.35	0.625
High school (14)	−0.15	(−0.41, 0.12)	6.41	0.234

*Note*: *N* of studies = 24, *N* of effect sizes = 113, assumed *ρ* = 0.8, *I* ^2^ = 78.83. Cluster robust estimates with *df* < 4.00 should be interpreted with caution (Tanner‐Smith, Tipton, & Polanin, 2016). Wald test for null difference between preschool, middle and high school grade levels, *F*(3, 2.82) = 0.41, *p* = 0.761. Positive effect sizes favor Montessori over traditional education.

Abbreviation: CI, confidence interval.

^a^ Reference category/intercept.

###### Public Montessori versus private Montessori

Both private and public Montessori education had better academic outcomes than traditional education, as Table 7 shows. Relative to traditional education, public Montessori education had academic outcomes 0.13 standard deviations less than private Montessori programs (Hedges' *g* = 0.28, 95% CI [0.03, 0.52]. However, in terms of the academic effectiveness of public Montessori over traditional education and the relative effectiveness of private Montessori over public Montessori, the wide 95% CI indicates that there is much statistical uncertainty regarding the degree to which these results would generalize to the entire population of students.

**Table 7:** Cluster robust meta‐regression for public versus private Montessori setting for academic effect sizes.

Effect (*n* of effect sizes)	Hedges’ *g*	95% CI of *g*	*df*	*p*
Private Montessori (31)^a^	0.28	(0.03, 0.52)	7.38	0.031
Public Montessori (76)	−0.13	(−0.36, 0.12)	12.64	0.238

*Note*: *N* of studies = 20, *N* of effect sizes = 107, assumed *ρ* = 0.8, *I* ^2^ = 71.89. Cluster robust estimates with *df* < 4.00 should be interpreted with caution (Tanner‐Smith, Tipton, & Polanin, 2016). Positive effect sizes favor Montessori over traditional education.

Abbreviation: CI, confidence interval.

^a^ Reference category/intercept.

###### Treatment duration and follow‐up measurements

**Figure 11:**
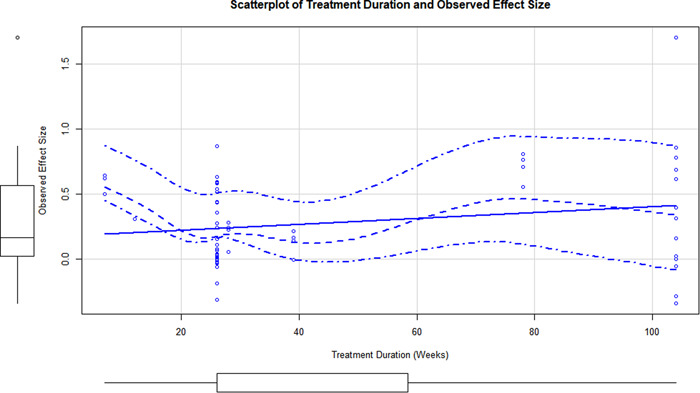
Scatterplot of treatment duration and observed effect size for academic outcomes. Positive effect sizes favor Montessori education over traditional education.

Table 8 is a meta‐regression table showing Montessori education's relative academic effectiveness over traditional education as a function of treatment duration (i.e., the duration of the Montessori intervention in number of weeks). Within‐study effects refer to effects that estimate growth over time on the same set of students within a study. The interpretation of the within‐subjects effect (Hedges' *g* = 0.007, 95% CI [−0.087, 0.100]) is that for every 1 week in Montessori education, the effect size increased by 0.007 standard deviations. Figure 11 is a scatterplot of treatment duration and between‐study effect size. Between‐study effects estimate growth over time by comparing treatment duration effects between different studies. The interpretation of the between‐subjects effect (Hedges' *g* = 0.002, 95% CI [−0.005, 0.009]) is that for every 1 week in Montessori education, the effect size increased by 0.002 standard deviations.

**Table 8:** Cluster robust meta‐regression for treatment duration (weeks) for academic effect sizes.

Effect	Hedges’ *g*	95% CI of *g*	*df*	*p*
Intercept^a^	0.188	(−0.045, 0.420)	9.13	0.101
Within‐study effect by week	0.007	(−0.087, 0.100)	1.00	0.538
Between‐study effect by week	0.002	(−0.005, 0.009)	2.36	0.353

*Note*: *N* of studies = 18, *N* of effect sizes = 67, assumed *ρ* = 0.8, *I* ^2^ = 80.72. Cluster robust estimates with *df* < 4.00 should be interpreted with caution (Tanner‐Smith, Tipton, & Polanin, 2016). Positive effect sizes favor Montessori over traditional education.

Abbreviation: CI, confidence interval.

^a^ Reference category/intercept.

While both within‐study and between‐study estimates indicate a slightly positive linear relationship between effect size and duration in Montessori education, the wide 95% CIs, the *df*s less than four, the nonlinearity, and nonconstant variance of the relationship between effect size and duration lead us to conclude that there is too much uncertainty and ambiguity to interpret these treatment duration results with any meaningful degree of certainty. In short, the results for this moderator were inconclusive.

Four studies included in this review used follow‐up measurements of academic outcomes after the Montessori intervention period had ended. We present the results of that analysis in Table 9. The results ostensibly show a slight fading effect on academic outcomes the longer a student had been out of Montessori education. Specifically, academic effect sizes decreased by 0.07 standard deviations per year after a student finished Montessori education (Hedges' *g* = −0.07, 95% CI [−0.16, 0.01]). Because of the low *df* (1.35) and the nonlinearity and nonconstant variance in the scatterplot, we suggest that readers interpret these results as largely inconclusive.

**Table 9:** Cluster robust meta‐regression for follow‐up years for academic effect sizes (between).

Effect	Hedges’ *g*	95% CI of *g*	*df*	*p*
Intercept^a^	0.92	(0.15, 1.68)	1.06	0.041
Follow‐up years	−0.07	(−0.16, 0.01)	1.35	0.062

*Note*: *N* of studies = 4, *N* of effect sizes = 37, assumed *ρ* = 0.8, *I* ^2^ = 31.85. Cluster robust estimates with *df* < 4.00 should be interpreted with caution (Tanner‐Smith, Tipton, & Polanin, 2016). Positive effect sizes favor Montessori over traditional education.

Abbreviation: CI, confidence interval.

^a^ Reference category/intercept.

##### Sensitivity analysis for academic outcomes

A sensitivity analysis examines the degree to which methodological or analytical decisions covary with outcomes (Higgins, 2021). In this review, we conducted several types of sensitivity analyses, the results of which would be too voluminous to detail here in entirety. Therefore, we summarize some of the results of the sensitivity analysis narratively in this paragraph and concentrate on one particularly important sensitivity analysis in the remainder of this section.

In terms of the *ρ* parameter (i.e., the estimated correlation between dependent effect sizes) used in cluster‐robust effect size estimation, the results were consistent regardless of the value of the parameter for *ρ* (i.e., the estimated correlation between dependent effect sizes) we chose. We conducted leave‐one‐out analyses for applicable outcomes and also found that the leave‐one‐out results were consistent with what was reported here.

For main effects analyses, we compared random‐effects and cluster‐robust synthesis methods. The overall results were consistent in terms of point estimates of effect size; however, as expected, the variance estimates differed slightly between random‐effects and cluster‐robust models. The random‐effects models tended to have lower variance than the cluster‐robust models, but we used cluster‐robust models nonetheless because we believed that the benefits of taking into account the dependencies between effect sizes outweighed slightly more accurate variance estimates, as discussed in Tanner‐Smith (2014).

Fixed‐effect models had smaller 95% CIs than cluster‐robust or random‐effects models, as expected, and were less favorable to Montessori education. We attribute this to the fact that fixed‐effect models tend to weight studies with large samples heavily compared to other models and fixed‐effect models do not take into account studies with multiple effect sizes (Higgins, 2021). We believe that Culclasure (2018) was overweighted in fixed‐effect models because it tended to contribute many effect sizes and had sample sizes in the thousands. As we discuss in the Discussion section, Culclasure (2018), as well as Ansari (2014), lacked consistently high treatment fidelity in the implementation of Montessori education and, thus, these studies might likely suppress the effect size in favor of traditional education.

One sensitivity analysis of particular importance was related to our method of effect size estimation. We used either a method for standardized mean differences, a standardized mean raw score method, or an *other* method where we calculated an effect size using Wilson (n.d.)'s Practical Meta‐Analysis Effect Size Calculator. The results of that sensitivity analysis are shown in Table 10. For academic outcomes, the mean difference in effect sizes between the reference category and other categories was less than 0.04 standard deviations in absolute value and the results of the omnibus Wald test yielded a *p* value of 0.955, *F*(2, 2.26) = 0.05. In this analysis the *dfs* were low and, therefore, these estimates should be interpreted with caution. Nonetheless, with all of the evidence taken together, we believed that it was reasonable to conclude that the method of effect size estimation was not an important moderator of effect size results and, therefore, it is justifiable to aggregate effect sizes across estimation methods as we did in the majority of analyses in this review.

**Table 10:** Cluster robust meta‐regression for effect size calculation method for academic effect sizes.

Effect (*n* of effect sizes)	Hedges’ *g*	95% CI of *g*	*df*	*p*
Other method^a^ (5)	0.26	(−0.41, 0.93)	1.00	0.929
Standardized mean raw score change (31)	−0.04	(−0.84, 0.76)	1.31	0.763
Standardized mean difference (77)	−0.00	(−0.52, 0.51)	1.44	0.509

*Note*: *N* of studies = 24, *N* of effect sizes = 113, assumed *ρ* = 0.8, *I* ^2^ = 72.71. Cluster robust estimates with *df* < 4.00 should be interpreted with caution (Tanner‐Smith, Tipton, & Polanin, 2016). Positive effect sizes favor Montessori over traditional education.

Abbreviation: CI, confidence interval.

^a^ Reference category/intercept.

Finally, for those interested in null hypothesis testing, we calculated the Benjamini–Hochberg (Benjamini, 1995; Benjamini, 2005) adjusted α to help readers account for the multiplicity of statistics tests (see Wasserstein, 2019), at least for main effects. A list of those adjustments can be found in the online supplement (Randolph, 2021).

##### Possible publication bias for academic outcomes

**Figure 12:**
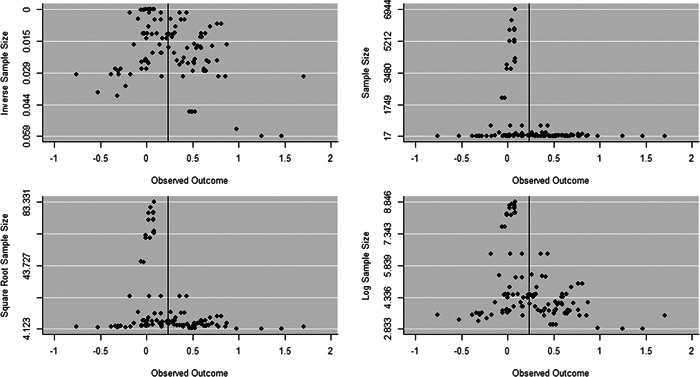
Four sample‐size based funnel plot variations for a composite of all academic outcomes combined.

Publication bias is a type of systematic bias that occurs when studies with null or negative effect sizes are withheld from the research record, either because of authors not submitting null or negative results for publication or journal reviewers or editors tending to not recommend studies with null or negative results for publication. To examine publication bias, we first conducted a visual analysis of a variety of funnel plots for all academic outcomes combined (see the four panels in Figure 12). In these funnel plots, the vertical axis represents some measure of sample‐size‐related variability (inverse sample size, raw sample size, square root of the sample size, log sample size) and the horizontal axis represents the effect size with positive effect sizes favoring Montessori education. Each data point within each funnel represents the correspondence of the standard error and effect size for each effect size. In general, larger studies will have less variance and, therefore, will be located near the top of the funnel whereas small studies will be located near the bottom.

Deviations from a funnel shape *may* be potential indicators of publication bias. See Higgins (2021) for more information on the visual analysis of funnel plots. One particular deviation to note is when a funnel plot is asymmetrically in favor of the intervention. Asymmetrical funnel plots with a large number of data points outside of the funnel only on the side favoring the intervention may be an indicator of publication bias in favor of the intervention. As a result of too many data points falling outside the funnel, there may be blank areas on the other side of the funnel. This deviation is seen to some degree in many of the main‐effect funnel plots presented in Figure 12. There is a preponderance of small studies in favor of Montessori education and an absence of small to medium size studies in favor of traditional education. The result is that the center reference line is also slightly to the right of the couple of studies with many effect sizes with very large sample sizes (e.g., Culclasure, 2018). As we explain in the Discussion section, there are some characteristics of the large sample studies that might attenuate their effect sizes in favor of traditional education. In addition, some of the variability of the handful of extreme outliers on the right can be explained through an analysis of study characteristics. The supplemental information (Randolph, 2021) contains funnel plots for individual academic outcomes.

In addition to a visual examination of funnel plots, we also conducted a trim and fill analysis. Using Duval's (2000) trimandfill algorithm enabled us to determine the number of effect sizes that may have been missing due to publication bias, impute the missing effect sizes to minimize funnel plot asymmetry, and then estimate a new effect size with the imputed effect sizes included.

**Figure 13:**
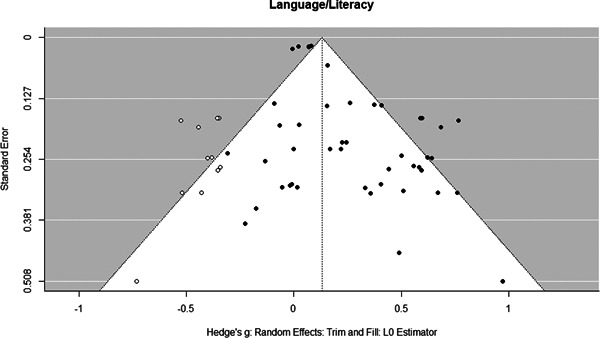
Funnel plot with trim and fill for language/literacy. The black data points represent observed effect sizes. The white data points represent effect sizes that were imputed using Duval's (2000) trim and fill algorithm to bring symmetry to the funnel plot. Without imputation, Hedges’ *g* was 0.24 compared to 0.13 when effect sizes were imputed.

We provide an example of that procedure here starting in terms of the language/literacy outcome. Figure 13 is a funnel plot in which the black data points represent the 46 observed effect sizes for language/literacy. The 11 data points in white on the lefthand side of the funnel plot represent the effect sizes imputed by the trim and fill algorithm to bring symmetry to the funnel plot as a whole. The value of Hedges' *g* for language/literacy was 0.24 (95% CI: 0.06, 0.36) for the 46 black data points. After imputing the 11 white data points and pooling them with the original effect sizes, the value of Hedges' *g* decreased to 0.13 (95% CI: 0.03, 0.23) for the new set of 57 effect sizes (i.e., the 46 original effect sizes and 11 imputed effect sizes). Duval (2000) notes that the trim and fill effect size should *not* be interpreted as a publication‐bias‐adjusted effect size. Instead, they recommend that one should use the difference between the imputed and unimputed effect sizes to gauge the degree of potential bias for a given outcome. For example, the 0.11 standard deviation difference between the imputed and unimputed effect size estimates is a rough estimate of the degree to which publication bias may be present in the Montessori research regarding language and literacy.

Table 11 shows the trim and fill results for the other academic outcomes. Since language/literacy contributed 45 effect sizes when all academic outcomes were combined, it is no surprise that there was statistical evidence of asymmetry for that composite outcome. After imputing 20 left‐side effect sizes, the values of Hedges' *g* for all academic outcomes combined decreased by about 1/10th of a standard deviation‐‐from 0.24 (95% CI: 0.17, 0.29) to 0.14 (95% CI: 0.07, 0.23).

**Table 11:** Academic effect sizes with and without left‐side trim and fill imputation.

Outcome	*N* of effect sizes	Estimated *N* of missing effect sizes (*SE* of *N*)	Hedges’ *g* (95% CI) without Imputation	Hedges’ *g* (95% CI) with L0 trim and fill imputation
Composite of academic outcomes	113	21 (6.99)	0.23 (0.17, 0.29)	0.14 (0.06, 0.22)
General academic ability	24	0 (2.99)	0.21 (0.06, 0.36)	NA
Language/literacy	46	11 (4.42)	0.24 (0.16, 0.33)	0.13 (0.03, 0.24)
Mathematics	36	0 (3.64)	0.28 (015, 0.42)	NA

*Note*: Outcomes with an *NA* indicate that there was enough symmetry that the trim and fill algorithm did not estimate any effect sizes to be missing and, therefore, no effect sizes were imputed. Random‐effects effect size estimates were for imputed and nonimputed estimates to facilitate comparison; therefore, these effect size estimates may differ from the cluster‐robust estimates presented elsewhere. Social studies and science were not included in this analysis because they had less than 10 effect sizes. L0 means left–side imputation.

Abbreviation: CI, confidence interval.

In summary, we found strong evidence for a publication bias effect on the language literacy outcome. We estimate that the publication bias effect for language/literacy is on the order of about 1/10th of a standard deviation in a way that is systematically biased in favor of Montessori education. However, we believe that the degree of systematic bias in favor of Montessori education is not sufficient to negate the overall finding that Montessori education increases language/literacy outcomes when compared to traditional education.

#### 5.3.2 Nonacademic outcomes

##### Main effects for nonacademic outcomes

**Figure 14:**
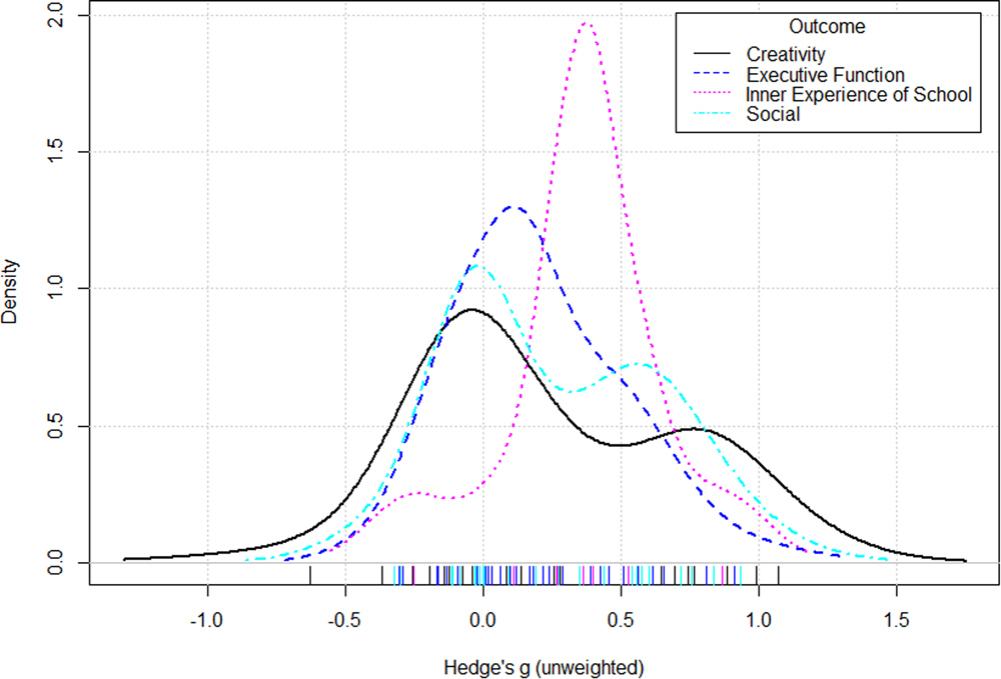
Kernel density plot of unweighted values of Hedges’ *g* for individual nonacademic outcomes.

Table 12 and Figure 14 (a histogram of effect sizes for nonacademic outcomes) show the main effects for all nonacademic outcomes combined and individually for the nonacademic outcomes of creativity, executive function, inner experience of school, and social skills. On average, Montessori students performed 0.33 standard deviations higher than traditional students on nonacademic outcomes, 95% CI [0.16, 0.50], with moderate quality of evidence.

**Table 12:** Main effects of Montessori education versus traditional education for all nonacademic.

Outcome	Hedges’ *g*	95% CI of *g*	*df*	*p*	*I* ^*2*^	*N* of studies	*N* of effect sizes
All nonacademic outcomes	0.33	0.16, 0.50	16.2	0.001	85.73	18	91
Creativity	0.26	−0.21, 0.74	4.76	0.209	74.49	6	24
Executive function	0.36	0.15, 0.58	9.26	0.004	80.41	11	34
Inner experience of school	0.41	0.19, 0.62	3.92	0.007	66.61	5	10
Social skills	0.23	−0.02, 0.49	7.30	0.070	82.92	9	23

*Note*: Cluster robust estimates with *df* < 4.00 should be interpreted with caution (Tanner‐Smith, Tipton, & Polanin, 2016). Positive effect sizes favor Montessori over traditional education.

Abbreviation: CI, confidence interval.

^a^ A cluster‐robust model was used for all outcomes except social studies, in which a random‐effects model was used. The social studies outcome had effect sizes from only one study.

**Figure 15:**
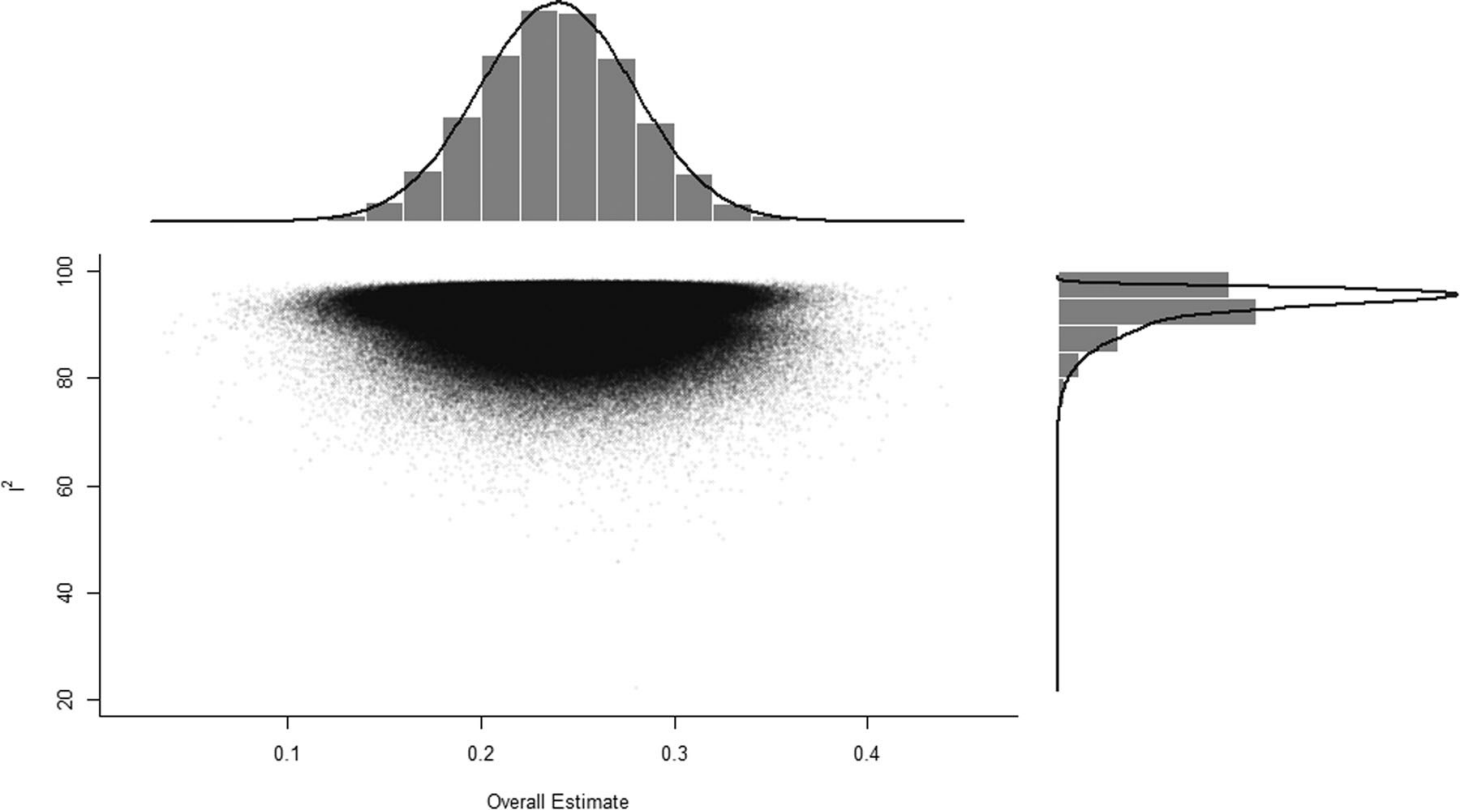
Graphical display of study heterogeneity for all nonacademic outcomes combined. In the GOSH plot above, the *X*‐axis (Overall Estimate) shows the Hedges’ *g* effect size. Positive effect sizes favor Montessori education over traditional education. The *Y*‐axis represents a measure of study heterogeneity, *I* ^2^, where higher values represent more study heterogeneity. The black data points are simulated values of the population effect size and heterogeneity.

See Figure 15 for a graphical display of study heterogeneity and effect sizes of academic outcomes from a bootstrap analysis of 1,000,000 samples of our nonacademic effect sizes. This plot shows that the plausible values of the summary effect size for nonacademic outcomes range approximately from 0.10 to 0.35 with high study heterogeneity.

**Figure 16:**
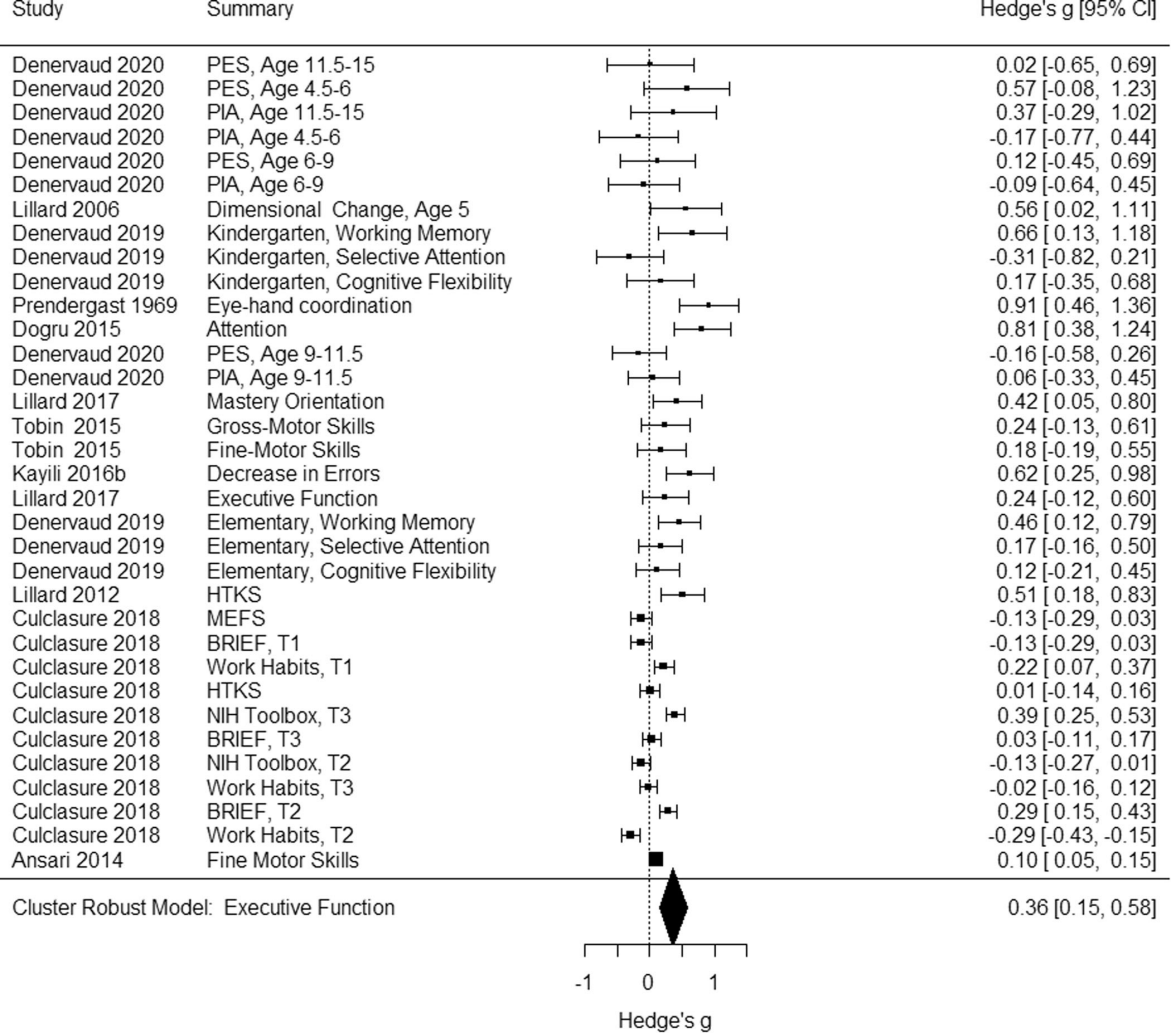
Forest plot for executive function. Positive effect sizes favor Montessori education over traditional education.

**Figure 17:**
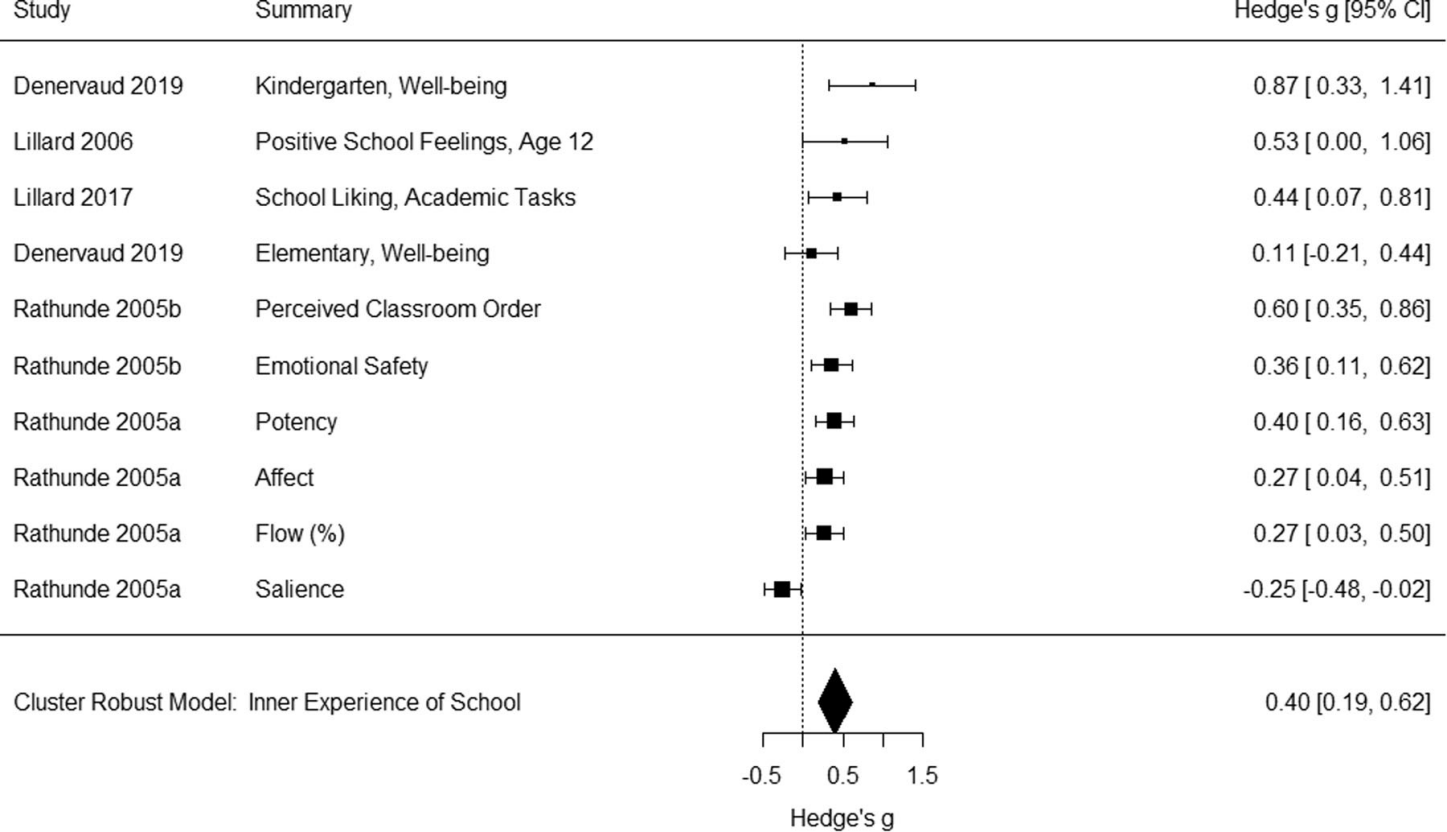
Forest plot for inner experience of school. Positive effect sizes favor Montessori education over traditional education.

**Figure 18:**
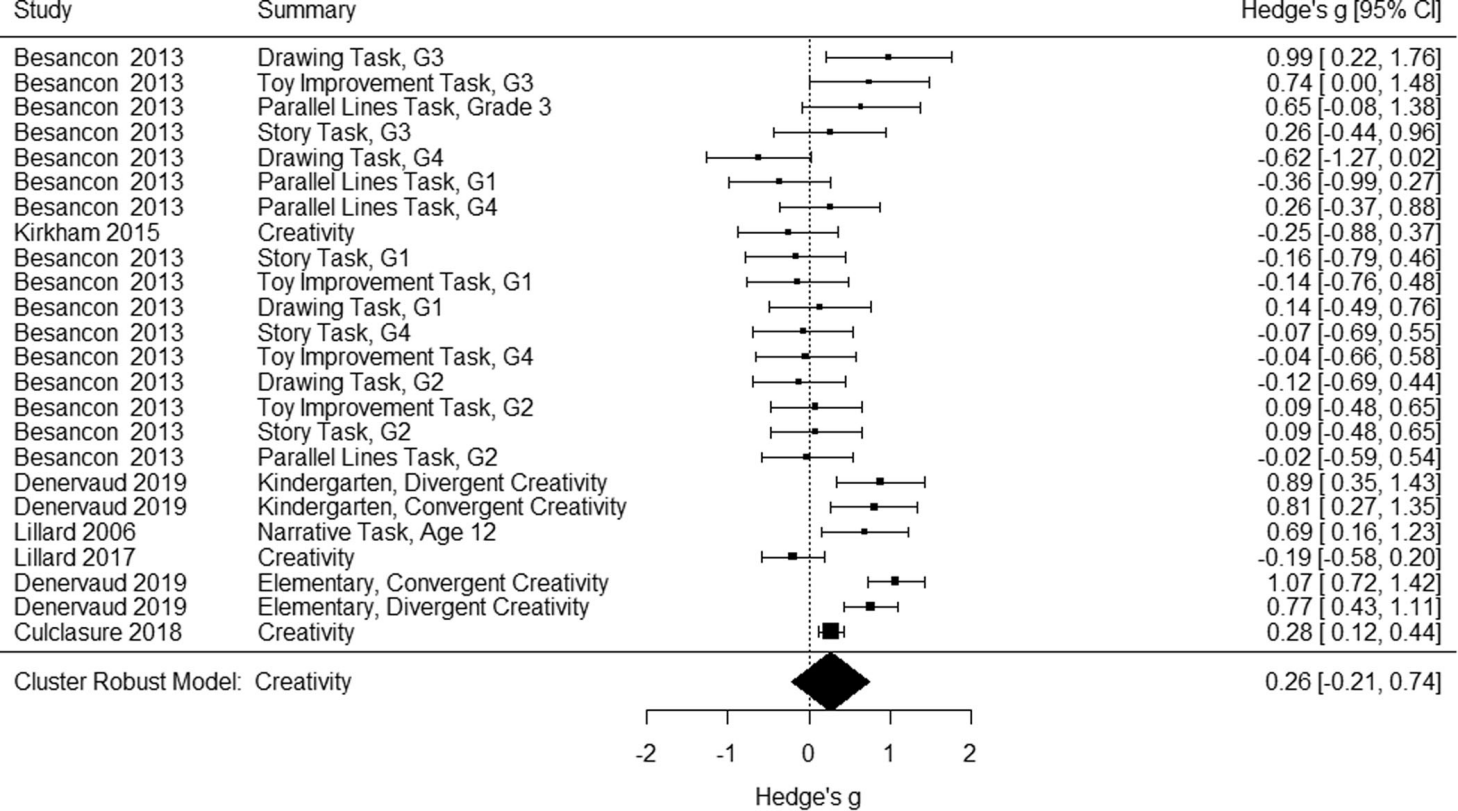
Forest plot for creativity. Positive effect sizes favor Montessori education over traditional education.

**Figure 19:**
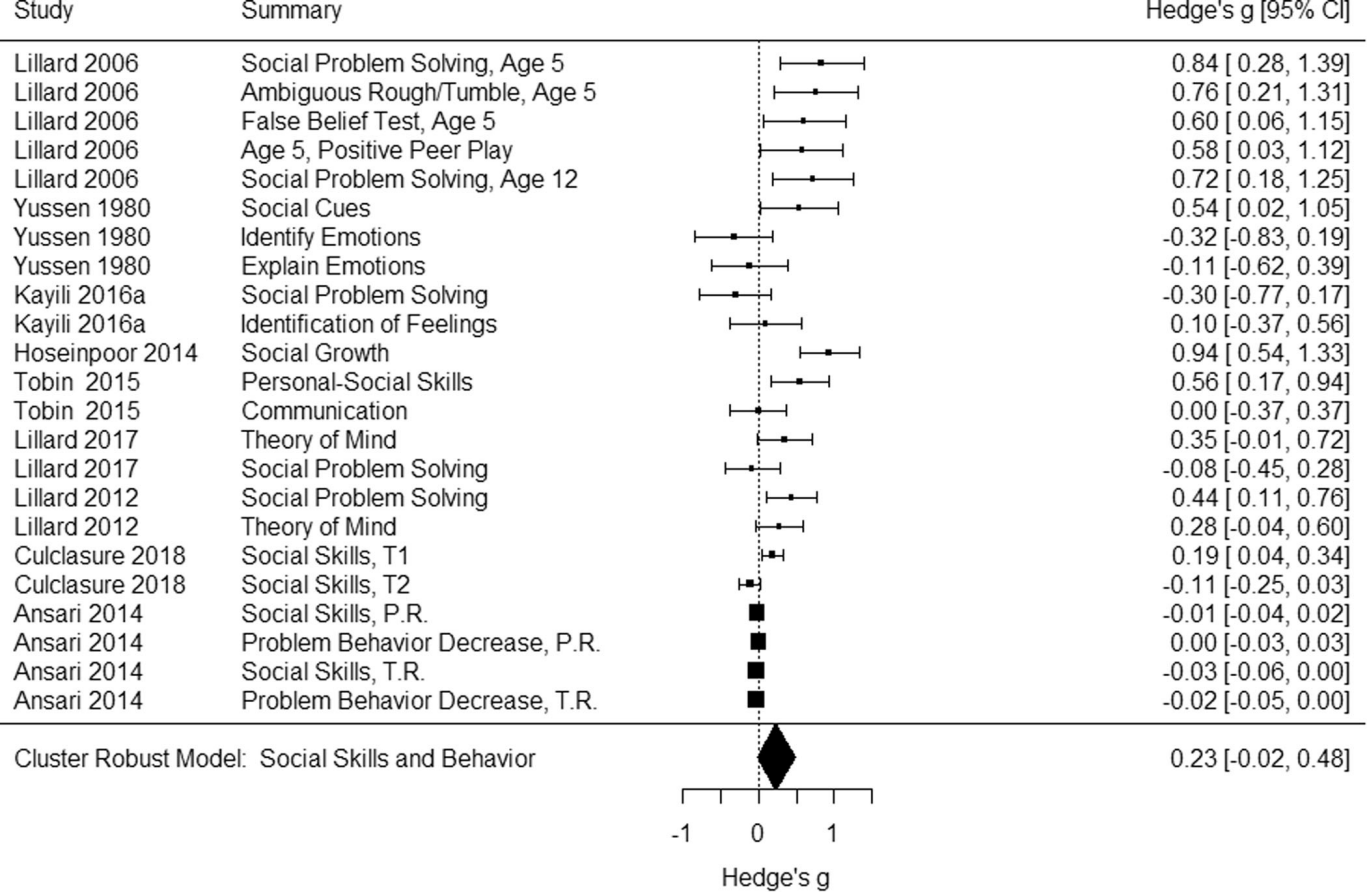
Forest plot for social skills and behavior. Positive effect sizes favor Montessori education over traditional education.

In terms of the individual four nonacademic outcomes shown in Table 12, there was moderate quality evidence that Montessori education outperformed traditional education. The largest effect sizes were found for executive function (Figure 16) (Hedges' *g* = 0.36, 95% CI [0.15, 0.58]) and inner experience of school (Figure 17) (Hedges' *g* = 0.41, 95% CI [0.19, 0.62]). However, the cluster‐robust *df* for inner experience of school (*df* = 3.79) was slightly under 4.00, indicating that the result should be interpreted with caution (Tanner‐Smith, 2016). While there were also small to moderate effects in favor of Montessori education for creativity (Figure 18) (Hedges' *g* = 0.26, 95% CI [−0.21, 0.74]), and social skills (Figure 19) (Hedges' *g* = 0.23, 95% CI [−0.02, 0.49]), there was significant variation in the 95% confidence intervals. There was also evidence of high study heterogeneity for nonacademic outcomes as demonstrated by the high values of *I* ^2^ in Table 10, which ranged from 82.92% for social skills to 66.61% for inner experience of school. Further, while creativity and executive function had moderate quality of evidence, inner experience of school and social skills both had low quality of evidence. See the Summary of findings Table 1 for more information on the certainty of evidence. Overall, there is moderate quality of evidence that Montessori education leads to better performance on nonacademic outcomes than traditional education.

##### Moderator analysis for nonacademic outcomes

Table 13 shows the meta‐regression results for random versus nonrandom assignment for all nonacademic outcomes combined. Similar to the results for nonacademic outcomes, effect sizes from studies with random assignment had an effect size 0.31 standard deviations greater than effect sizes without random assignment; however, there was significant variance in the plausible range of values for this estimate, 95% CI [−0.54, 1.16] and the *df* < 4.0.

**Table 13:** Cluster‐robust meta‐regression for random assignment for nonacademic effect sizes.

Effect (*n* of sizes)	Hedges’ *g*	95% CI of *g*	*df*	*p*
Nonrandom assignment^a^ (76)	0.28	(0.10, 0.45)	13.20	0.005
Random assignment (15)	0.31	(−0.54, 1.16)	2.75	0.315

*Note*: *N* of studies = 18, *N* of effect sizes = 91, assumed *ρ* = 0.8, *I* ^2^ = 84.12a. Cluster‐robust estimates with *df* < 4.00 should be interpreted with caution (Tanner‐Smith, Tipton, & Polanin, 2016). Positive effect sizes favor Montessori over traditional education.

Abbreviation: CI, confidence interval.

^a^ Reference category/intercept.

Similar to the effects for academic outcomes, Montessori education had greater impacts than traditional education at all measured grade levels on nonacademic outcomes as shown in Table 14. (There were no included studies with nonacademic effect sizes for high school students). The greatest impacts of Montessori education for nonacademic outcomes were seen at the preschool level (Hedges' *g* = 0.36, i.e., 0.26 + 0.10 = 0.36). However, a Wald test for an omnibus effect of a grade‐level moderator was not statistically significant (*p* = 0.863). In summary, Montessori education had the greatest impacts over traditional education on nonacademic outcomes at the preschool level; however, there was much statistical uncertainty about grade level as a moderator of the effectiveness of Montessori education.

**Table 14:** Cluster‐robust meta‐regression for grade level for nonacademic effect sizes.

Effect (*n* of effect sizes)	Hedges’ *g*	95% CI of *g*	*df*	*p*
Elementary school^a^ (39)	0.26	(−0.24, 0.76)	4.93	0.235
Preschool (44)	0.10	(−0.39, 0.59)	9.29	0.646
Middle school (8)	0.05	(−0.72, 0.83)	2.83	0.834

*Note*: *N* of studies = 18, *N* of effect sizes = 91, assumed *ρ* = 0.8, *I* ^2^ = 85.60. Cluster‐robust estimates with *df* < 4.00 should be interpreted with caution(Tanner‐Smith, Tipton, & Polanin, 2016). Wald test for null difference between preschool and middle school grade levels, *F*(2, 3.39) = 0.10, *p* = 0.911. Positive effect sizes favor Montessori over traditional education.

Abbreviation: CI, confidence interval.

^a^ Reference category/intercept.

As Table 15 shows, both private and public Montessori education showed greater impacts on nonacademic outcomes than traditional education. This result was especially strong for private Montessori education (Hedges' *g* = 0.43, 95% CI [0.18, 0.67]). Private Montessori outperformed public Montessori on nonacademic outcomes. There is statistical uncertainty, however, about the degree to which these results would accurately generalize to the population of students.

**Table 15:** Cluster‐robust meta‐regression for public versus private Montessori setting for nonacademic effect sizes.

Effect (*n* of effect sizes)	Hedges’ *g*	95% CI of *g*	*df*	*p*
Private Montessori (34)^a^	0.43	(0.18, 0.67)	6.63	0.005
Public Montessori (36)	−0.26	(−0.52, 0.06)	8.55	0.100

*Note*: *N* of studies = 13, *N* of effect sizes = 70, assumed *ρ* = 0.8, *I* ^2^ = 78.75. Cluster‐robust estimates with *df* < 4.00 should be interpreted with caution (Tanner‐Smith, Tipton, & Polanin, 2016). Positive effect sizes favor Montessori over traditional education.

Abbreviation: CI, confidence interval.

^a^ Reference category/intercept.

**Figure 20:**
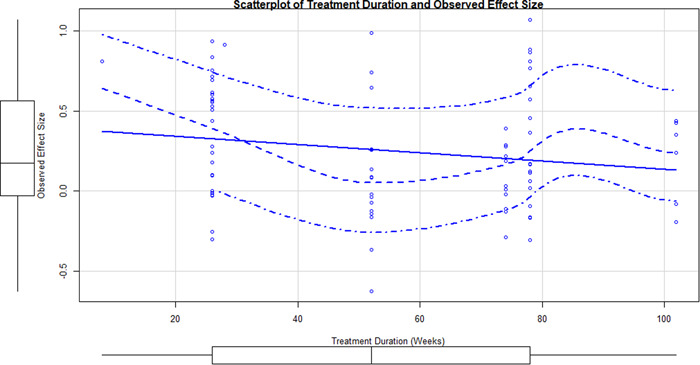
Scatterplot of treatment duration (weeks) and observed effect size for nonacademic outcomes. Positive effect sizes favor Montessori education over traditional education.

Table 16 and Figure 20 show the results of an examination of nonacademic effect sizes as a function of treatment duration. The within‐study and between‐study effects were contradictory; the within‐study effect showed a very slight treatment‐duration effect in favor of Montessori education (Hedges' *g* = 0.001, 95% CI [−0.089, 0.091)] and the between‐study effect showed the same treatment‐duration effect but in favor of traditional education, (Hedges' *g* = −0.004, 95% CI [−0.011, 0.003] for nonacademic outcomes. For the same reasons explained in the section on treatment duration for academic outcomes, we regard these results as inconclusive. There were too few studies to do a moderator analysis of follow‐up studies for nonacademic outcomes.

**Table 16:** Cluster‐robust meta‐regression for treatment duration (weeks) for nonacademic effect sizes.

Effect	Hedges’ *g*	95% CI of *g*	*df*	*p*
Intercept^a^	0.524	(0.126, 0.922)	9.63	0.015
Within‐study effect by week	0.001	(−0.089, 0.091)	1.00	0.913
Between‐study effect by week	−0.004	(−0.011, 0.003)	4.62	0.167

*Note*: *N* of studies = 15, *N* of effect sizes = 67, assumed *ρ* = 0.8, *I* ^2^ = 86.65. Cluster‐robust estimates with *df* < 4.00 should be interpreted with caution (Tanner‐Smith, Tipton, & Polanin, 2016). Positive effect sizes favor Montessori over traditional education.

Abbreviation: CI, confidence interval.

^a^ Reference category/intercept.

##### Sensitivity analysis for nonacademic outcomes

The results of the sensitivity analysis for nonacademic outcomes yielded nearly identical results as for academic outcomes: the difference in results between analytical methods was unremarkable. In summary, the results were consistent regardless of the value of the parameter for ρ that was chosen. The leave‐one‐out results differed only marginally from the effect sizes reported when all studies were included. For main effects analyses, when comparing random‐effects and cluster‐robust synthesis methods, the overall results were consistent in terms of point estimates of effect size; however, as expected, the variance estimates differed slightly between random‐effects and cluster‐robust models. Although not reported here, the fixed‐effect models had smaller 95% CIs and were less favorable of Montessori education, likely due to implementation in the large sample studies that will be discussed later.

In terms of the differences between effect sizes rendered using different estimation methods for nonacademic outcomes (see Table 17), there was a large effect size difference between the standardized mean raw score change method and the *other* method (Hedges' *g* = −0.45, 95% CI [−2.36, 1.45]) and between the standardized mean difference method and the *other* method (Hedges' *g* = −0.54, 95% CI [−2.11, 1.01]), as shown in Table 13. However, the mean difference between the two standardized methods, which consisted of the majority of effect sizes, was only 0.06 standard deviations and the Wald test for omnibus differences yielded a *p* value of 0.382, *F*(2, 1.57) = 1.57. This suggests we are justified in synthesizing results regardless of the method we used to calculate a study's effect size.

**Table 17:** Cluster‐robust meta‐regression for effect size calculation method for nonacademic effect sizes.

Effect (*n* of effect sizes)	Hedges’ *g*	95% CI of *g*	*df*	*p*
Other method^a^ (3)	0.78	(−1.95, 3.51)	1.00	0.171
Standardized mean raw score change (46)	−0.45	(−2.36, 1.45)	1.28	0.273
Standardized mean difference (42)	−0.54	(−2.11, 1.04)	1.37	0.197

*Note*: *N* of studies = 18, *N* of effect sizes = 91, assumed *ρ* = 0.8, *I* ^2^ = 83.46. Cluster‐robust estimates with *df* < 4.00 should be interpreted with caution (Tanner‐Smith, Tipton, & Polanin, 2016). Wald test for null difference between standardized mean raw score change and standardized mean difference effect size approaches, *F*(2, 1.9) = 2.12, *p* = 0.337. Positive effect sizes favor Montessori over traditional education.

Abbreviation: CI, confidence interval.

^a^ Reference category/intercept.

##### Possible publication bias for nonacademic outcomes

**Figure 21:**
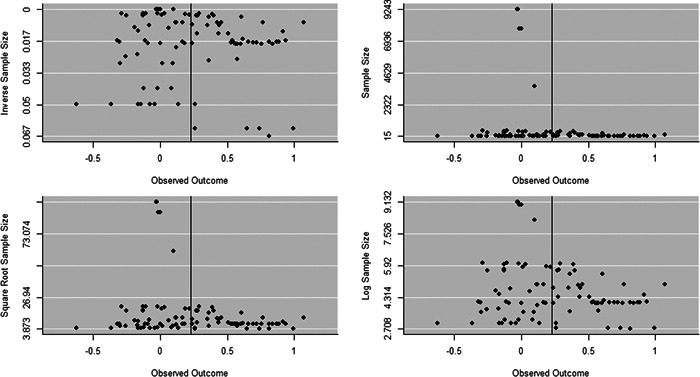
Four sample‐size based funnel plot variations for nonacademic outcomes.

Figure 21 is a four‐panel funnel plot of all nonacademic outcomes combined. The same asymmetry of small studies in favor of Montessori education that was seen for academic outcomes is also seen here for nonacademic outcomes. Similarly, we believe that some of the variance can be explained through an analysis of study characteristics of the extreme outlying studies and of the studies with a very large sample size, as explained in the Discussion. We conclude that there is some slight asymmetry in small studies in favor of Montessori education but it is unclear whether publication bias is the cause. Asymmetry alone is not sufficient evidence for publication bias (Higgins, 2021). Overall, because of the small degree of symmetry and the analytical methods used, we conclude that the asymmetry does not change the substantive conclusion that Montessori education is favorable for most nonacademic outcomes. Conservatively, we believe that the range of plausible values of the aggregate effect of Montessori education on nonacademic outcomes is represented well in the GOSH plot shown in Figure 15.

Table 18 shows the results with a trim and fill analysis to help interpret the degree of publication bias. Similar to the trim and fill analysis conducted for academic outcomes, we estimate that publication bias favors Montessori by about 1/10th of a standard deviation.

**Table 18:** Nonacademic effect sizes with and without left‐side trim and fill imputation.

Outcome	*N* of effect sizes	Estimated *N* of missing effect sizes (*SE* of *N*)	Random‐effects Hedges’ *g* (95% CI) without imputation	Random‐effects Hedges’ *g* (95% CI) with L0 trim and fill imputation
Composite of nonacademic outcomes	91	9 (6.14)	0.23 (0.15, 0.30)	0.16 (0.08, 0.25)
Creativity	24	0 (2.95)	0.25 (0.05, 0.45)	NA
Executive function	34	6 (3.88)	0.18 (0.07, 0.28)	0.09 (−0.03, 0.21)
Inner experience of school	10	3 (2.13)	0.33 (0.12, 0.54)	0.22 (0.03, 0.42)
Social skills	23	5 (3.21)	0.22 (0.07, 0.38)	0.10 (−0.07, 0.28)

*Note*: Outcomes with an *NA* indicate that there was enough symmetry that the trim and fill algorithm did not estimate any effect sizes to be missing and, therefore, no effect sizes were imputed. Random‐effects effect size estimates were for imputed and nonimputed estimates to facilitate comparison; there, these effect size estimates may differ from the cluster‐robust estimates presented elsewhere. L0 means left–side imputation.

Abbreviation: CI, confidence interval.

## 6 DISCUSSION

Accumulative evidence is required for scientists and policy decision‐makers to draw conclusions about the efficacy of an intervention (Popper, 1962/2014). When the evidence is mixed, as was concluded by two recent narrative reviews of Montessori outcomes (Ackerman, 2019; Marshall, 2017), meta‐analyses can resolve the ambiguity by providing a more precise effect size estimate based on a large set of observations drawn from multiple studies and samples (Rosenthal, 2002). The Campbell reviews reflect this view, and the current meta‐analysis was conducted to shed light on the efficacy of Montessori education for children's academic and nonacademic outcomes, testing the hypothesis that Montessori is at least as effective as traditional education. The meta‐analysis included only studies with evidence of baseline equivalence, through random assignment to Montessori, and/or through pretest, matching, or statistical adjustment on key variables. A search of the literature using the search term “Montessori” and snowballing from reference lists resulted in a list of over 2000 published and unpublished studies, but as with other school choice meta‐analyses (e.g., Cheng, 2017), the vast majority of the 173 studies deemed worthy of closer inspection were excluded for failing to meet the stringent inclusion criteria that are more suggestive of causal effects.

Thirty‐two studies that were conducted before February 2020 when data collection closed did meet the criteria. They examined a broad array of outcomes, from academic ones like mathematics and literacy to social‐emotional outcomes like creativity and social skills. Because single studies contributed several effects, most analyses used cluster–robust regression. The studies included public and private schools, and participants ranging from preschool through high school; subsequent analyses examined these and random/nonrandom assignment as moderators.

In contrast to the aforementioned recent narrative reviews that were neutral on the efficacy of the Montessori approach, this quantitative meta‐analysis showed that Montessori education had largely positive effects on outcomes, with effect sizes that ranged from small to large in the context of school‐based field research with standardized (non‐custom) measures (Kraft, 2020). Below we summarize these findings, separating academic from nonacademic outcomes. Note that we believe our findings might have been unduly influenced (in a direction that is unfavorable to Montessori) by a single study with a very large *N* that also contributed many effects; this study included schools with varying quality of Montessori implementation as assessed by that study's own fidelity of implementation measurement. This is discussed later.

### 6.1 Summary of main results

Here we summarize the academic and then the nonacademic findings of our analysis. The outcomes reported here refer generally to all students, not to particular subgroups such as lower‐income children. In addition, we note that several of the included studies have small *N*s, and studies with small *N*s typically have larger effect sizes to be published. However, our bias analyses revealed relatively little evidence of publication bias, rendering this less of a concern.

#### 6.1.1 Academic outcomes

Both overall and by individual outcome, the meta‐analysis showed that Montessori's average effect on academic outcomes was uniformly positive for Montessori, with Hedges' *g*s ranging from 0.26 (general academic ability, including composites) to 0.05 (social studies). Where math was reported separately, the effect size was 0.22 (based on 36 effects from 12 studies), and for literacy, it was 0.17 (based on 45 effects from 16 studies). For research on school programs (which 28 of the 32 included studies concerned), these are noteworthy effect sizes. In a discussion of effect sizes for school studies involving standardized test scores (most of the academic tests included here), the math finding would be considered large and the literacy effect size medium‐large (Kraft, 2020). One recent meta‐analysis using school lotteries found the effect of charter school attendance to be much smaller than the effects observed here (Cheng, 2017); specifically, it found average effects on ELA (English Language Arts) of about one‐tenth of a standard deviation (0.09), and on mathematics of about two‐tenths of a standard deviation (0.19). Results of charter schools using the “no excuses” model specifically were comparable to those achieved by Montessori (0.25 and 0.17). We note that the charter school analyses typically were of older grades, and effect sizes typically decline across school years (Hill, 2008). The Montessori effect sizes for science and social studies were considerably smaller, and the lower end of their confidence intervals was negative, but these analyses included only three and one studies (respectively) and just five and three effect sizes, thus we have less confidence in those findings.

Looked at another way, Montessori education's overall impact on academic performance is about a quarter of a standard deviation, equivalent to about 2 months of school in Grade 1, and the whole school year in Grade 6 (Hill, 2008). These equivalences were derived by Hill et al. by looking at the average change in children's scores across a school year; children change much more early in schooling, when learning to read and do elementary math, than they do in older grades. The month equivalence is derived from dividing the average school‐year change scores by the number of months children are in school (typically 9).

The academic results are important because they resolve existing ambiguity regarding Montessori's effects. While many studies of Montessori report positive outcomes, the domains attaining significance are not entirely consistent across studies, and there is at least one relatively highly cited published study with one negative academic effect (Lopata, 2005), which did not meet the inclusion criteria for this review. In such circumstances, meta‐analytic results are very important (Rosenthal, 2002). Montessori is a constructivist educational approach (Elkind, 2003) and is sometimes equated with discovery learning approaches because children have considerable independence, being free to choose what to work on each day (Lillard, 2017). Discovery learning approaches typically do not have positive academic outcomes when compared with more traditional direct instruction (Klahr, 2004; Mayer, 2004). Studies of Montessori outcomes, like those included in this meta‐analysis, most often use traditional direct instruction as the comparison, although some use a more specific counterfactual (like HighScope in the Ansari, 2014 study) and others used conventional U.S. preschools from an earlier era in which preschool was mostly free play. The results of this meta‐analysis indicate that on average, across studies, academic outcomes in Montessori are better than those of traditional education, however it was defined in a given study. The implementation range was broad, as described in Types of interventions, reflecting the broad range of programs dubbed “Montessori” in the real world; the same is true for the traditional school implementations. Thus, this study likely reflects the real‐world difference between Montessori and traditional school. The effect sizes are similar to those rendered in a meta‐analysis of oversubscribed “no excuses” charter schools, in which the counterfactual is typically underperforming urban public schools.

#### 6.1.2 Nonacademic outcomes

The average effect size was slightly stronger for nonacademic outcomes (0.33) than academic ones (0.24). Executive function yielded the largest effect, with a Hedges' *g* of 0.36 based on 34 effects from 11 studies. This is approaching the near transfer effect of targeted executive function training programs for children (0.44), and far exceeds the effect of far transfer training (0.11, Kassai, 2019), which Montessori arguably is: Montessori has no explicit training on common measures of executive function like opposites games. However, it may provide outsized experiences in exercising inhibitory control which is an aspect of executive function (see below). Because executive function is a significant predictor of both concurrent (Jacob, 2015) and future outcomes including health, wealth, and criminality (Moffitt, 2011), this Montessori result is highly significant. There are several potential routes by which Montessori might impact the development of executive function (Lillard, 2017). For example, it has many parallels to mindfulness training, such as emphasizing and cultivating concentrated attention, educating the senses to notice fine gradations in stimuli, taking great care in every movement of the body, and sometimes sitting or walking for a period of time in purposeful silence. Mindfulness training appears to influence executive function through an impact on sustained attention (Leyland, 2019; Zoogman, 2015), and was observed in a meta‐analysis to influence social‐emotional outcomes more generally (Maynard, 2017). Another, simpler route to inhibitory control specifically is that children have to wait in Montessori because there is only one of most resources. Because there is one copy of each material, if another child is using a material, other children who want to use that material must wait. In addition, properly implemented Montessori has only one teacher (Lillard, 2019a; Lillard, 2019b), who moves around the classroom helping or teaching children one by one; if a child needs the teacher's help, he or she often must wait for individual attention. Further research on individual children's behaviors in Montessori classrooms could shed light on what aspects of the program might influence executive function development.

Montessori also had a nontrivial impact on one's inner experience of school, which translates to well‐being at school (Hedges' *g* = 0.41); although this result stems from just 10 effects and five studies and has lower evidence quality, its size makes it likely that there is some impact. It makes theoretical sense that Montessori would lead to higher well‐being. For example, self‐determination is associated with higher well‐being (Ryan, 2000), and in Montessori environments, relative to conventional school environments, children are given considerable freedom as long as they use that freedom to constructive ends for their own and others' development (Montessori, 2017). People who attended Montessori as children have higher adult well‐being (Lillard, 2021) and recall liking school better during childhood (LeBoeuf, 2022).

Another nonacademic outcome that is related to Montessori was creativity, with a Hedges' *g* of 0.26; the 95% CI around this effect ranged from −0.21 to 0.74, so the result, to which six studies contributed 24 effect sizes, should be interpreted cautiously. It is conceivable, but not clear from this evidence, that the freedom to consider possibilities (Rinke, 2013), combined with a lack of extrinsic rewards and multiple‐choice tests (see Lillard, 2017), fosters creativity in Montessori. Another effect that is less clear is social skills, with a Hedges' *g* of 0.23 and a 95% CI from −0.02 to 0.49, from nine studies and 23 effect sizes. It is possible that the multi‐aged classrooms and sustained relationships with peers due to looping enable advances in social skills; alternately, advanced social skills could be a by‐product of increased executive function. Again, we have less confidence in the social skills and creativity effects.

In sum,

- Montessori education yielded strong and clear effects on math, literacy, general academic ability, and executive function,
- Montessori education's effects on aspects of well being, such as the inner experience of school and school liking, also were strong and appeared reliable, and
- Montessori education also appeared to affect social studies, science, creativity, and social skills, but these effects are less clear and need further study.

#### 6.1.3 Potential moderators

##### Study design: Random versus nonrandom assignment

The major obstacle to drawing conclusions about outcomes from freely chosen school programs is selection bias. One can control for family income, ethnicity, and other factors, but the possibility that families who choose Montessori differ in some other way that accounts for the results remains. Ensuring equality on key variables at pretest is helpful since presumably the family factors that cause a child to be higher on some outcome variable would be present at the outset as well. Controlling for the level of that variable at pretest is also helpful, but if the family produces an exponential growth pattern on that variable, it is uncontrolled. Because family effects that might positively affect child outcomes are confounded with Montessori enrollment, one would expect effects from nonrandom experimental designs to be greater than for random assignment studies. Interestingly, here this was not the case. Rather, results were considerably stronger for studies using random assignment for academic effects, whereby random assignment raised the effect size of 0.19 found with studies that used pretests or control variables to ensure baseline equivalence, to almost 1/3 of a standard deviation (i.e., 0.31). For nonacademic effects, the nonrandom assignment effect size of 0.28 more than doubled, adding 0.31 standard deviations. Just six studies had random assignment; three used data from children randomly assigned to a Montessori intervention at age four through 10th grade, with results published in three included papers (earlier reporting on that same study was not included because effect sizes could not be calculated from the information provided therein), and found strong sleeper effects for children previously enrolled in a Montessori Head Start, especially for boys. Two studies involved lotteries at oversubscribed American public schools. The sixth study was of 60 children enrolled in preschools in a city in Iran; it randomly assigned half the children to a targeted Montessori intervention. It contributed just two effects and the report is not sufficiently clear to allow speculation regarding the reason for its strong effects.

Focusing on the other studies, we speculate that their outcome effects stem from study timing in one case (representing three papers), and implementation of Montessori in the other two. Regarding the former, it might be that even weak Montessori implementation has long‐term effects because philosophical elements like free choice and order can exist even when the structural aspects of implementation are weak; these philosophical elements might not always manifest in differences in the near term, but could manifest later. The Miller studies, which contributed many effect sizes to the random effect, did not have particularly strong Montessori implementation (the original Miller, 1975 study reported an implementation score of 6.5/10), but it did provide for free choice and other philosophical elements, and this early experience at four might have led to long‐term outcome differences relative to the counterfactual (which was a traditional free play school). Another study of long‐term academic outcomes (not included here because it lacked appropriate controls) also found better academic performance for Montessori students (with unclear program implementation) even years after they had left the program (Dohrmann, 2007). Other studies suggest that without regard to implementation, Montessori predicts better nonacademic outcomes (LeBoeuf, 2022; Lillard, 2021).

The two Lillard random lottery studies looked at the immediate effects of Montessori, but the three oversubscribed public schools in the two studies were particularly strong examples of Montessori implementation. Although oversubscription is not itself a clear indicator of school quality (Tuttle, 2012; Weiland, 2020), here the oversubscribed schools were all AMI (Association Montessori Internationale) recognized Montessori schools. AMI schools adhere to the structural elements (i.e., they hire AMI‐trained teachers, which is an intensive 9‐month, standardized training with a highly prepared and vetted trainer; they have the specific 3‐year age spans, scarce adults and high ratios; and the long work periods and full set of Montessori materials) and they also implement the philosophical elements well. By contrast, teachers trained by the other major training organization, the American Montessori Society (AMS), founded to “Americanize” Montessori education (Rambusch, 1992), are relatively more supportive of conventional American education practices like tests, due dates, worksheets, and whole‐class activities (Daoust, 2018, April), which might dilute the immediate effects of the Montessori program as compared to traditional education. Two studies specifically examined the influence of implementation on outcomes and found that the more classic implementation espoused by AMI is associated with better outcomes than supplemented implementations (Lillard, 2012; Lillard, 2016).

In sum, we speculate that the randomized trials had better outcomes in this meta‐analysis because sleeper effects on outcomes occur in response to philosophical aspects of Montessori that exist even when structural implementation is weak, and because two of the random studies, while looking at immediate outcomes, had especially high fidelity Montessori implementation.

To shed further light on why random assignment had an unexpectedly stronger effect than nonrandom assignment in this meta‐analysis, more research is needed using random assignment while also coding for Montessori implementation. We would hypothesize that among studies using random assignment, those using a more classic Montessori implementation would have stronger effects.

##### Age level

Academic effects were strongest for children in Elementary school, at 0.36. As is typical, effects were smaller across middle and high school; effect sizes for academic achievement across a school year decline fairly steadily from K to 12 (Hill, 2008). However, for Montessori, effects at preschool (0.20) were also smaller than effects at Elementary school (0.36). Yet the preschool effects are still notable. For example, one random‐assignment study of Head Start, with children enrolled either at age three or age four, found that 13 of 22 effects on language, literacy, and math were significant, and *of those that achieved significance*, the average effect size was 0.18 *SD* (Barnett, 2011), similar to our preschool effect which was not limited to those that achieved significance. The greatest gains for most children are typically seen from Kindergarten to first grade (Hill, 2008) when children undergo the famous “5 to 7 shift” (Sameroff, 1996) with biological changes augmenting environmentally‐induced ones. In Elementary school, the average growth across each school year is 0.44 (Lipsey, 2012), thus Montessori education may add as much as 80% of the gains expected in an entire school year to children's achievement. Although effect sizes were calculated for middle and high school and were considerably smaller, very few studies contributed to effect sizes at those levels giving us less confidence in those effects. Nonacademic effects were strongest for young children, which may indicate that at those ages children's executive function, creativity, and so on are most malleable. There are few developmental studies of these abilities, thus we were unable to view these results comparatively.

##### Public versus private Montessori

As Table 7 showed, both public and private Montessori educational interventions had better results than traditional education on aggregated academic outcomes. Public Montessori schools were shown to have an effect size 0.13 standard deviations lower than private Montessori schools on aggregate academic outcomes. This difference is equivalent to 29% of the average growth expected in a school year for elementary students (Lipsey, 2012). Nonetheless, the 95% CIs showed that the range of plausible values for this estimate varied from public Montessori education's performing 0.36 standard deviations lower than private Montessori education, which is equivalent to 82% of the average yearly academic growth expected in school in elementary education, to public Montessori education's performing 0.12 standard deviations *above* private Montessori education, which is equivalent to 27% of the average academic growth expected in elementary education. Therefore, we conclude that private Montessori education is likely to achieve academic results as good as or somewhat better than public Montessori education.

As Table 15 showed, both public and private Montessori educational interventions had better results than traditional education on aggregated nonacademic outcomes. Private Montessori had an effect size of 0.43 for aggregated nonacademic outcomes; public Montessori had an effect size of 0.26 standard deviations less than private Montessori. The effect size differences between public Montessori and private Montessori were twice as great for nonacademic outcomes, which had a 0.26 effect size difference in favor of private Montessori, as academic outcomes, which had a 0.13 effect size difference in favor of private Montessori. Because there are few longitudinal studies of the nonacademic outcomes reported here, it is not possible to view these effects in terms of expected yearly growth.

In summary, we conclude that there is preliminary evidence that private Montessori education leads to moderately greater performance in both academic and nonacademic outcomes than public Montessori education. We can also conclude that there is preliminary evidence that this difference in improvement for private Montessori and public Montessori is likely to be greater for nonacademic outcomes than academic outcomes. We argue that the evidence is *preliminary* because of the statistical imprecision of our estimates.

The public–private difference might be due to implementation. Public schools are required to have children take state exams that are designed for traditional school programs; Montessori schools need to adjust their program to prepare for those tests, and this by necessity dilutes Montessori implementation at public schools. Public schools are also required to follow regulations about recess breaks, special classes in art and sports, add‐in curricula, and teacher‐child ratios and class sizes that compromise the integrity of the Montessori program. Private schools also sometimes make such compromises, but they are more free to determine their programs.

### 6.2 Overall completeness and applicability of evidence

Our searches included a variety of academic databases, sources known to publish gray literature, Montessori‐related journals, and manual searches of references in retrieved studies. This search led to over 1500 unique records and, of those, 173 were included for full‐text review. Of those, 32 studies met the criteria for inclusion. The exhaustiveness of our search procedure and the number of records found lead us to believe that the Montessori‐related research listed in the excluded or included studies sections here represents a nearly complete, or at least, representative set of the quantitative Montessori research literature published before the specified search period, which ended February 2020. The outcomes also represent a broad variety of academic and nonacademic outcomes.

Also, because of the thoroughness of the search method, we conclude that the results presented here are applicable over a wide variety of traditional education and Montessori settings. However, when deciding the degree to which these results might generalize to particular settings or to future studies, consider these notes:

- The majority of studies were conducted in elementary or pre‐K settings.
- North American studies were predominant, but there was some degree of international representation from Europe, the Middle East, and Asia.
- We only included studies that met strict requirements for demonstrating baseline equivalency; we suspect that including studies that met less stringent baseline‐equivalency requirements would have led to somewhat different effect sizes.

In summary, we are confident that the results presented here are drawn from a complete or nearly complete set of studies published during the search period and that the results are applicable over a wide variety of traditional and Montessori settings, to the most common academic and nonacademic outcomes, and across multiple measures of the given construct.

### 6.3 Quality of the evidence

The results of the risk of bias assessments indicated that the risk of bias in the included studies was low overall. Nonexperimental studies tend to have moderate or high risk of bias (Higgins, 2021), and we attribute our nonexperimental studies' having low risk of bias to our stringent inclusion criteria.

When applying the GRADE criteria, the quality of evidence was downgraded because of lack of precision (e.g., as measured by a nonstatistically significant result), high heterogeneity (e.g., a high *I* ^*2*^ value), and/or asymmetry of funnel plots, which can be an indicator of publication bias. If there were publication bias, we anticipate it would have led to marginally smaller effect sizes since there was a set of studies with small/medium sample sizes and larger effect sizes than would have been expected given chance. We suspect the treatment fidelity may have been compromised in large sample size studies, which tend to have more weight in a meta‐analysis than studies with small sample sizes and which tended to contribute more effect sizes than smaller studies. Therefore, we upgraded the quality of evidence for outcomes that included the large‐sample studies (Culclasure, 2018 and/or Ansari, 2014) because we believed it was a plausible confounding factor that may have underestimated the treatment effect. We assumed that greater treatment fidelity should result in greater effect sizes in favor of Montessori education over traditional education.

In summary, we conclude that there is moderate to high quality evidence to support our findings for most academic and nonacademic outcomes. See the Summary of Findings table 1 for more‐detailed information on the quality of evidence.

### 6.4 Potential biases in the review process

One limitation that could have biased these results is that we were unable to determine the quality of Montessori implementation in five of the included studies, and in several others we based our estimates on article descriptions that varied in completeness. We have noted that this does reflect the state of Montessori education in the real world. If a study claimed to be a test of Montessori education versus traditional education, and had evidence of equivalent baseline by accounting for key variables or by a lottery design in which children whose parents applied to oversubscribed schools were admitted or not admitted at random, then its findings were included. Some of the studies were done at AMI (Association Montessori Internationale) recognized schools (Denervaud, 2019; Denervaud, 2020; Lillard, 2006; Lillard, 2012; Lillard, 2017; Mix, 2017; Rathunde, 2005a; Rathunde, 2005b; and Yussen, 1980) and another at a school accredited by the Swiss Montessori Association, suggesting high fidelity implementation, but for some studies, implementation quality was known to be of lower quality; for example, both the Ansari (2014) and the three Miller studies (Jones, 1979; Miller, 1983; Miller, 1984) took place at Montessori preschools that had only 4‐year‐olds, missing the key ingredient of a 3‐year cycle and age grouping.

Culclasure (2018) is important to discuss in this regard, because it weighed heavily in our analysis due to its many effects (26) and very large sample (thousands of children contributing to each effect). Culclasure studied outcome differences for children in 23 public South Carolina Montessori schools. Only schools that passed minimal criteria for being Montessori were included in the study, but even among those included, implementation varied widely. Culclasure had trained Montessori teachers observe in a random selection of 126 classrooms and rate implementation, and they also had teacher surveys. Although half the programs were considered high fidelity by the study's own metrics, the other half were of medium or low fidelity. The teacher survey indicated that 35% of teachers did not think they had all or even most of the materials they needed to teach Montessori. Also, 90% said they used circle time centered around a weekly theme, suggesting they were more teacher‐directed than is optimal, and 43% said they supplemented the Montessori program with other materials (which Lillard (2012) and Lillard (2016) suggest leads to less positive outcomes than does using only Montessori materials). Only 30% introduced the Great Lessons in the first half of the school year, and over half of the teachers surveyed said they felt the quality of the Montessori was declining, and that public school test requirements were a major reason. Thus, although the Montessori implementation was proper in many ways (e.g., very few teachers used extrinsic rewards), this exemplary study that contributed many heavily weighted effect sizes to our study had weaknesses in its Montessori implementation. If those weaknesses reduce effect sizes, then this study alone might have biased results in a negative direction for Montessori.

Because implementation varies widely in Montessori schools (Daoust, 2004; Daoust, 2018; Daoust, 2019), and some studies suggest that better outcomes are achieved when implementation is more closely aligned with Montessori principles and practices (Lillard, 2016; Lillard, 2019a; Lillard, 2019b), the lack of attention to implementation, or sufficient reporting of implementation fidelity, in some of the studies included in this review is a limitation. The heterogeneity of implementation could be a reason for the asymmetry observed in some of our funnel plots. We expect that effect sizes would be greater had Montessori interventions been completed with greater fidelity. A future analysis might examine whether effect sizes increase as the level of implementation increases.

The effect sizes for the social studies outcome only came from one study (Culclasure, 2018). Therefore, we urge readers to note this important limitation when considering the result.

We did not extract data on whether control and experimental groups were assigned to the same school or whether the unit of randomization was at the individual, classroom, or school level. We suggest that this information be extracted and considered in future reviews.

Another potential bias is that the comparison samples/school programs are heterogeneous. It was outside of the scope of this review to compare Montessori to any specific alternative program. Thus, although virtually all the control children were receiving traditional or business–as–usual education, traditional schools vary. Although some specified that this meant teacher‐led, whole‐class learning using mostly lectures and textbooks, others did not. Our comparison could be described as average Montessori versus average traditional programs. We are unable to say which specific Montessori implementation is better or worse–only that compared to a range of other choices, on average, it produces positive effects.

Other sources of potential bias concern the categorization of the measures into the outcome categories we chose. Others might categorize some outcomes differently. Categorization was done without consideration of any potential impact on results, but it is possible that different categorization choices would yield significantly different results. Our database and codes are available for interested readers to review our categorization choices and redo the analyses with alterations to examine their impact.

Another potential limitation of the meta‐analysis is that many studies of Montessori education, including the ones studied here, had relatively small samples (two exceptions are the studies by Ansari, 2014 and Culclasure, 2018). Effect sizes tend to be larger with small‐*N* studies (Kraft, 2020), and this was evident in the funnel plots for many of the outcomes we presented here. Therefore, it is likely that if more studies had been done with large sample sizes, the effect sizes would have been smaller than those reported here. We believe that the GOSH plots (Figures 5 and 15) robustly display the plausible ranges of population effect size estimates. Furthermore, we have included the code and data set so that researchers can investigate methodological variations that are too cumbersome to report here (e.g., fixed vs. random effects).

There was some evidence (asymmetry in funnel plots) that may point to publication bias in favor of Montessori. The trim and fill estimates in Tables 11 and 18 provide a general estimate of what the population effect sizes might be if data were imputed to make the funnel plot of effect sizes be symmetrical. We encourage Montessori researchers to attempt to publish the results of methodologically sound studies regardless of whether the results are negative, null, or positive and, thereby, help create a more comprehensive research record.

One might be concerned that one of the authors of this meta‐analysis (Lillard) also authored three of the included studies. Lillard joined the team after the initial analyses were done, and was not involved in devising the selection criteria, nor in deciding which studies to include, nor in the analyses. Thus, the results were obtained without any opportunity for author bias. Her role was limited to interpretation and writing, summarizing the fidelity of implementation of the interventions, as well as reviewing the broader literature and contextualizing the results.

### 6.5 Agreements and disagreements with other studies or reviews

Very few reviews of the efficacy of Montessori education have been published in peer‐reviewed journals. The results of this meta‐analysis of 32 studies are consistent with an earlier meta‐analysis that only included two Montessori studies, both unpublished, and focused exclusively on achievement outcomes; it calculated a *d* of 0.27 (Borman, 2003), similar to our overall academic effect size (Hedges' *g* = 0.24). Considering qualitative reviews, which were neutral, the results of this analysis reflect more positively on Montessori. Ackerman (2019) concluded that “Montessori programs have the potential to enhance young children's learning and development” (p. 11) but that there was no consistent advantage. Her review had included studies with less rigorous designs than those included here, and lack of rigor in the existing experimental base was a main conclusion of the Marshall (2017) review as well. Marshall stated that random lottery experiments were essential and also that studies' transposing elements of Montessori into other systems is a useful way to figure out what in Montessori leads to benefits.^1^

## 7 AUTHORS' CONCLUSIONS

### 7.1 Implications for practice

Like the Marshall (2017) review, many discussions of Montessori end with the question of what causes the benefits. Given that we have a logical positivist science, this is a natural response. But we suggest instead adopting a systems perspective, recognizing that Montessori is a complex system, comprised of a set of attitudes and beliefs, practices, and materials (Lillard, 2019). Montessori was very concerned with the training of the teacher, and Montessori teacher training (at least in its traditional form) is said to require a spiritual transformation, creating a disposition of flexibility, restraint, and love of humanity (Whitescarver, 2007). The teacher takes a Rousseauian attitude that given the right conditions (including inspiration and curiosity, which the teacher helps to inspire), children will be kind and happy, and will behave in ways that are constructive for self and society. The teacher believes in, indeed loves, every child and is sure of the Montessori system's capacity to achieve this. The practices include structural elements such as specific 3‐year age groupings, a high teacher‐child ratio (about 30:1, plus an assistant for the youngest children), individual and small group lessons with a full set of specially constructed Montessori materials, and 2.5–3 h uninterrupted work periods sufficient for deep concentration. The practices also include philosophical elements like keeping the classroom beautiful and orderly, allowing children to choose freely as long as they are constructive, older children going out to explore the world and giving formal reports about their research activities to the class, and nothing beyond those specific Montessori practices (no grades, tests, or worksheets, no special outside teachers, and so on). Furthermore, in the practice of the AMI, the organization Montessori started to carry on her work, the teacher has gone through intensive training and examination, with a teacher‐trainer who themselves had intensive training in an apprenticeship to become a teacher‐trainer. The materials are sets of hundreds of mostly wooden and glass instruments and paper charts designed by Montessori and her collaborators for each age level to convey specific learning; children engage with these materials with their hands or even their full bodies (for further summary, see Lillard, 2019a; Lillard, 2019b). Children are also expected to have had Montessori at the prior level as well, at least from age three on, because Montessori confers specific learning that is bulit on later levels.

Which of these many elements is responsible for better academic and nonacademic outcomes? It may be the wrong question, although there are Montessori programs that eliminate one or more features. If enough programs were found that eliminated a specific one might compare those programs to programs that retain all the standard Montessori features.

For example, the Miller (Jones, 1979; Miller, 1983; Miller, 1984) and Ansari (2014) studies were not fully authentic implementations of Montessori for at least one reason: the classroom had only 4‐year‐olds. Some other studies also had limited age groups. The Mallett (2015) and Culclasure (2019) studies were done in public schools where Montessori preK was not necessarily offered. By contrast, some studies state that the school was recognized by AMI (Denervaud, 2019; Denervaud, 2020; Lillard, 2006; Lillard, 2012; Lillard, 2017; Mix, 2017; Rathunde, 2005a; Rathunde, 2005b), each of which suggests the full system was operating, or AMS, which has many elements but a different teacher training and often includes added practices (Daoust, 2018, April). However, most studies of Montessori do not give enough information for readers to discern if the program had all the elements just described. Measures of Montessori implementation are rare and have yet to be standardized. Furthermore, it is unclear whether objective observers without Montessori training could discern whether important aspects were being properly implemented. For example, an untrained observer would not likely recognize if a teacher was presenting a material properly.

It is the case that people have incorporated elements of Montessori, like looping or no grades or more specifically the practical life materials (Bhatia, 2015), in conventional classrooms and often seen better results (see Lillard, 2017 for a review), and this would seem to point at which elements of Montessori are responsible for the results. If conventional teachers could improve child outcomes by simply adapting certain elements of Montessori, that would be more practical than adopting the whole system, which requires retraining many thousands of teachers, purchasing vast amounts of new materials, and eliminating the textbook industry. Adopting even some elements might be worthwhile for improving outcomes. But if the systems perspective is correct, then adopting elements is a weak solution. An alternative, if Montessori is considered sufficiently superior to warrant widespread adoption, is to convert one school or district at a time to the full Montessori system, beginning in lower‐income districts where the need for improvement is greatest.

### 7.2 Implications for research

Further research on Montessori education should attend carefully to implementation; Montessori programs can vary widely, with those recognized by AMI having the strictest implementation, AMS the next most strict, and others sometimes using the name Montessori without implementing the program to any great degree (Daoust, 2004; Daoust, 2018; Daoust, 2019). Although we caution that Montessori is a system such that the whole is likely not the same as the sum of its parts (Lillard, 2019), determining whether and how different implementations influence outcomes is very important.

A second important question for further research not addressed here is examining subsamples such as lower‐income children and global majority children. Montessori education, after all, was first designed to serve the needs of lower income students (Montessori, 1964). We extracted some data on these variables for the included studies, but it was outside the scope of this review to do a detailed analysis. We encourage researchers to use our data (Randolph, 2021) to carry out this line of inquiry.

Future research should follow children in Montessori longitudinally. Montessori is most frequently a preschool program and follows a pyramid structure with the fewest programs available at high school. Most studies of Montessori have been limited to a single data collection point. A few have collected data twice, at pretest and posttest, and very few have followed a sample over several years (Culclasure, 2018; Lillard, 2017) or tested people several years after Montessori schooling was complete (Jones, 1979; Miller, 1983; Miller, 1984; see also Dohrmann (2007), not included here due to lack of baseline equivalency). Recent findings from the Tennessee study of prekindergarten, which showed initially positive gains for children randomly assigned to preK reversed by 3rd grade, raise concerns about early schooling in general (Lipsey, 2018). Lipsey (2018) suggested that this could be due to a lack of curricular alignment between preK and later experiences, with Elementary school teachers focusing on students who had not had preK because they had skill deficits; this lack of focus then led to their later problems. This issue would not be present for children continuing from Montessori preK to Montessori Elementary school programs, but that is very much a concern going from Montessori preK to traditional Elementary programs. Alternatively, Lipsey et al. suggest that the misalignment of preschoolers' developmental needs with the academic emphases of the Elementary schools in which public pre‐K programs are often housed might have contributed to the poor 3rd‐grade outcomes. The current analysis shows that Montessori's nonacademic effects at preschool were stronger than its academic effects (0.39, as opposed to 0.20). This might suggest that Montessori preschool would provide for more whole‐child development and thereby mitigate a later downturn, but longitudinal research is needed to ascertain if that is the case.

Another area important for future research involves experimental study design. There is a dearth of randomized trials and high‐quality nonexperimental designs that adequately account for baseline differences between treatment and control groups. The use of these designs will reduce the selection threat (Shadish, 2002), which we consider to be the most likely threat in the Montessori research because it is likely that students enrolled in private schools–the setting for most Montessori programs–will differ at baseline on important academic and nonacademic variables. We suggest that if researchers are unable to implement random assignment, they use a baseline variable, such as a pretest, that is a direct measure of the outcome and to account for baseline differences statistically by using the baseline variable as a covariate or by the use of gain scores.

Finally, in line with suggestions of the American Statistical Association (Wasserstein, 2019), we encourage more meta‐analyses of the Montessori research and are making our data publicly available (see Randolph, 2021) to encourage replications and extensions of our review. Permission is universally granted for the use of the inclusion/exclusion data and the data extracted from the included studies. Some ideas for follow‐ups to this review are provided below.

It may be meaningful for future Montessori meta‐analyses to be detailed examinations, in which potential moderators are explored, for each of the individual major outcomes with a sufficient number of studies to do so.

It may also be meaningful to use other analytical methods to see if results are consistent across analytical methods. We used a cluster–robust variance estimation procedure that accounts for dependencies in the data and makes accurate point estimates; however, that procedure is less powerful than other synthesis methods and, therefore, will yield variance estimates that are less precise (Tanner‐Smith, 2014; Tanner‐Smith, 2016; Tipton, 2015).

Furthermore, we used a strict methodological criterion in terms of baseline equivalency. It may be useful for authors to use a less strict inclusion criterion and, thereby, be able to include a large number of studies that were excluded because of a lack of strong evidence for baseline equivalency. Our inclusion/exclusion spreadsheet lists the studies that were excluded based on baseline equivalency criteria.

We found evidence of asymmetry in funnel plots, which may be an indicator of publication bias. We suggest that follow‐up studies examine the publication bias in further detail. Trim and fill methods like those discussed in Shi 2019 may be useful for imputing left‐side missing data (L_0_) and subsequently estimated the degree of bias resulting from study asymmetry.

Finally, we suggest that future research create a rating scale of Montessori and traditional implementation quality and use that as a potential moderator. We imagine that quality of treatment implementation could be an important variable in explaining the high amount of heterogeneity.

## CONTRIBUTIONS OF AUTHORS

Justus J. Randolph: Overall methodological design, statistical analysis, and programming, data collection, and extraction, supervision, grant writing and management, primary text writer of methods and results sections. Anaya Bryson: Data collection and extraction, primary text writer of introductory sections. Lakshmi Menon: Data collection and extraction. David K. Henderson: Data collection, management, and extraction, effect size calculation and confirmation, risk‐of‐bias coder, quality control of data. Austin Kureethara Manuel: Data collection, management, and extraction, effect size calculation, quality control of data, risk‐of‐bias coding. Stephen Michaels: Design and implementation of search strategy, study and data management, the primary writer of the search results section. Warren McPherson: Theoretical expert and contributor to the theoretical section. debra leigh walls rosenstein: Theoretical expert and contributor to the theoretical section. Rebecca O'Grady: Data collection and extraction. Angeline S. Lillard: Subject matter and theoretical expert, interpretation, supervision and funding acquisition, introduction and the primary writer of discussion and plain‐language summary.

## DECLARATIONS OF INTEREST

Justus J. Randolph's spouse is employed at a private Montessori school. Anaya Bryson has no declarations of interest. David K. Henderson has no declarations of interest. Lakshmi Menon has no declarations of interest. Austin Kureethara Manuel has no declarations of interest. Stephen Michaels has no declarations of interest. Warren McPherson and his spouse were cofounders of a private Montessori school. Mr. McPherson was the Director of the school for over 40 years until 2019. He and his spouse currently serve on the school's Board of Directors, without remuneration. He serves as a Montessori teacher‐trainer, consultant, and speaker and is remunerated for those activities. Debra leigh walls rosenstein has no declarations of interest. Angeline S. Lillard is the author of two studies in the meta‐analysis and was not involved in data extraction/coding/critical appraisal of those studies. She has spoken at many Montessori conferences and received remuneration. She has written a book on Montessori and its relation to developmental science and receives royalties. Finally, she attended Montessori from ages 3 to 6 and took a Montessori training course.

## PUBLISHED NOTES

Errata. In figures, the term Hedge's *g*, should be spelled Hedges' *g*.

**Characteristics of excluded studies** 2

**Table:** 

Ahmad 2018
**Reason for exclusion**
Ahmadpour 2015
**Reason for exclusion**
Baerman 2002
**Reason for exclusion**
Bagby 2012
**Reason for exclusion**
Baines 1973
**Reason for exclusion**
Bank 1969
**Reason for exclusion**
Banta 1970
**Reason for exclusion**
Bereiter 1967
**Reason for exclusion**
Berends 2018
**Reason for exclusion**
Berger 1969
**Reason for exclusion**
Besançon 2008
**Reason for exclusion**
Blanco‐Vega 2002
**Reason for exclusion**
Borman 2003
**Reason for exclusion**
Brand 1989
**Reason for exclusion**
Brophy 1973
**Reason for exclusion**
Brown 2015
**Reason for exclusion**
Brown 2016
**Reason for exclusion**
Byun 2013
**Reason for exclusion**
Castellanos 2002
**Reason for exclusion**
Chattin‐McNichols 1981
**Reason for exclusion**
Chisnall 2007
**Reason for exclusion**
Claxton 1982
**Reason for exclusion**
Coopmans 1984
**Reason for exclusion**
Corry 2006
**Reason for exclusion**
Cox 2000
**Reason for exclusion**
Dawson 1987
**Reason for exclusion**
De Luca 2006
**Reason for exclusion**
Dhiksha 2015
**Reason for exclusion**
Di Lorenzo 1968
**Reason for exclusion**
Di Lorenzo 1969
**Reason for exclusion**
Diamond 2010
**Reason for exclusion**
Diamond 2011
**Reason for exclusion**
Dohrmann 2003
**Reason for exclusion**
Dohrmann 2007
**Reason for exclusion**
Donabella 2008
**Reason for exclusion**
Dreyer 1969
**Reason for exclusion**
Duax 1989
**Reason for exclusion**
Epstein 1996
**Reason for exclusion**
Ervin 2010
**Reason for exclusion**
Esposito 2010
**Reason for exclusion**
Fero 1997
**Reason for exclusion**
Findlay 1913
**Reason for exclusion**
Fleming 2019
**Reason for exclusion**
Flower 2006
**Reason for exclusion**
Flynn 1991
**Reason for exclusion**
Franc 2015
**Reason for exclusion**
Franczak 2016
**Reason for exclusion**
Galliger 2009
**Reason for exclusion**
Glenn 1993
**Reason for exclusion**
Glenn 2003
**Reason for exclusion**
Gross 1970
**Reason for exclusion**
Grubb 2000
**Reason for exclusion**
Guidubaldi 1974
**Reason for exclusion**
Guven 2020
**Reason for exclusion**
Hanson 2009
**Reason for exclusion**
Haq 2015
**Reason for exclusion**
Harris 1995
**Reason for exclusion**
Harris 2007
**Reason for exclusion**
Heise 2010
**Reason for exclusion**
Hickerson 1983
**Reason for exclusion**
Hobbs 2008
**Reason for exclusion**
Hojnoski 2008
**Reason for exclusion**
İman 2017
**Reason for exclusion**
Jarvis 2015
**Reason for exclusion**
Johnston 2013
**Reason for exclusion**
Jones 2005
**Reason for exclusion**
Judge 1974
**Reason for exclusion**
Kamakil 2013
**Reason for exclusion**
Karnes 1969
**Reason for exclusion**
Karnes 1977
**Reason for exclusion**
Karnes 1978
**Reason for exclusion**
Karnes 1983
**Reason for exclusion**
Kayili 2011a
**Reason for exclusion**
Kendall 1993
**Reason for exclusion**
Kimmins 1915
**Reason for exclusion**
Kimmins 1915a
**Reason for exclusion**
Kohlberg 1968
**Reason for exclusion**
Krafft 1998
**Reason for exclusion**
Krogh 1979
**Reason for exclusion**
Lail 2017
**Reason for exclusion**
LaRue 2010
**Reason for exclusion**
Laski 2015
**Reason for exclusion**
Laski 2016
**Reason for exclusion**
Lillard 2005
**Reason for exclusion**
Lillard 2013
**Reason for exclusion**
Lillard 2016
**Reason for exclusion**
Lillard 2021
**Reason for exclusion**
Lopata 2005
**Reason for exclusion**
Mallett 2013
**Reason for exclusion**
Mallett 2014
**Reason for exclusion**
Manner 2007
**Reason for exclusion**
McCladdie 2006
**Reason for exclusion**
McDurham 2011
**Reason for exclusion**
McKinnon 1982
**Reason for exclusion**
Miezitis 1971
**Reason for exclusion**
Miller 1970
**Reason for exclusion**
Miller 1971
**Reason for exclusion**
Miller 1972
**Reason for exclusion**
Miller 1975
**Reason for exclusion**
Morfitt 1937
**Reason for exclusion**
Morgan 1978
**Reason for exclusion**
Mroczkowski 2014
**Reason for exclusion**
Murphy 1976
**Reason for exclusion**
Boehnlein 1990
**Reason for exclusion**
Pate 2014
**Reason for exclusion**
Peng 2009
**Reason for exclusion**
Peng 2014
**Reason for exclusion**
Phillips‐Silver 2018
**Reason for exclusion**
Rathunde 2003
**Reason for exclusion**
Reed 2000
**Reason for exclusion**
Reich 1974
**Reason for exclusion**
Reuter 1973
**Reason for exclusion**
Roberts 1976
**Reason for exclusion**
Rodriguez 2003
**Reason for exclusion**
Rose 2012
**Reason for exclusion**
Ruijs 2017
**Reason for exclusion**
Salazar 2013
**Reason for exclusion**
Sciarra 1976
**Reason for exclusion**
Sebastian 2016
**Reason for exclusion**
Seefeldt 1977
**Reason for exclusion**
Seefeldt 1981
**Reason for exclusion**
Shankland 2009
**Reason for exclusion**
Shernoff 2013
**Reason for exclusion**
Shivakumara 2016
**Reason for exclusion**
Simmons 1983
**Reason for exclusion**
Simons 1984
**Reason for exclusion**
Somorin 2016
**Reason for exclusion**
Stallings 1987
**Reason for exclusion**
Stephens 1973
**Reason for exclusion**
Stern 1968
**Reason for exclusion**
Stevens 1976
**Reason for exclusion**
Stodolsky 1972
**Reason for exclusion**
Szobiová 2014
**Reason for exclusion**
Tamminen 1967
**Reason for exclusion**
Toot 2019
**Reason for exclusion**
Weikart 1981
**Reason for exclusion**
Wexley 1974
**Reason for exclusion**
White 1976
**Reason for exclusion**
Witte 2013
**Reason for exclusion**
Yen 2000
**Reason for exclusion**

## SUMMARY OF FINDINGS

**1 Summary of findings**

**Table:** 

**Montessori education compared to traditional education on academic and nonacademic outcomes**				
**Population**: Students in grade levels ranging from preschool to high school				
**Settings**: Public or private schools				
**Intervention**: Montessori education				
**Comparison:** Traditional education				

**Outcome**	**Standardized mean difference (95% CI)** a	**Number of studies (Number of effect sizes) [Number of observations]**	**Certainty of the evidence (GRADE)**	**Explanation for certainty of evidence**
All academic outcomes combined	0.24 (0.13‐0.36)	24 (113) [83,910]	Moderate	Downgraded once for possible publication bias and once for inconsistency. Upgraded because we believe the studies with very large sample sizes were not implemented with fidelity.
General academic ability	0.26 (0.06‐0.46)	9 (24) [6636]	High	Downgraded once for inconsistency. Upgraded because we belive the intervention in the study with the very large sample size was not consistently implemented with fidelity.
Language/literacy	0.17 (0.03, 0.31)	16 (45) [36,254]	High	Downgraded once for possible publication bias. Upgraded because we believe the studies with very large sample sizes were not implemented with fidelity.
Mathematics	0.22 (0.06, 0.39)	12 (36) [20,280]	High	Upgraded because we believe the studies with very large sample sizes were not implemented with fidelity.
Science	0.14 (−0.61, 0.90)	3 (5) [10,301]	Low	Downgraded twice for imprecision (large CIs and *df* < 4.00) and once for inconsistency. Publication bias was not applicable since there were less than 10 effect sizes for this outcome. Upgraded because we believe the intervention in the study with the very large sample size was not consistently implemented with fidelity.
Social studies	0.05 (−0.02, 0.12)	1 (3) [10,440]	Moderate	Downgraded once for imprecision based on large CIs and once for inconsistency. Publication bias was not applicable since there were less than 10 studies. Imprecision based on a *df* < 4.00 was not applicable here because a cluster‐robust model was not used. Upgraded because we believe the intervention in the large sample size study was not consistently implemented with fidelity.
All nonacademic outcomes combined	0.33 (0.16‐0.50)	18 (91) [48,339]	Moderate	Downgraded once because of inconsistency and once for possible publication bias. Upgraded because we believe the studies with very large sample sizes were not implemented with fidelity.
Creativity	0.26 (−0.21, 0.74)	6 (24) [1218]	Moderate	Downgraded twice for the imprecision of the 95% CI and once for inconsistency. Upgraded because we believe the largest study's intervention was not consistently implemented with fidelity.
Executive Function	0.36 (0.15, 0.58)	11 (34) [9091]	Moderate	Downgraded once because of possible publication bias and once for inconsistency. Upgraded because we believe the studies with very large sample sizes were not implemented with fidelity.
Inner experience of school	0.41 (0.19, 0.62)	5 (10) [2002]	Low	Downgraded for imprecision based on *df* < 4.00 and once for publication bias.
Social skills	0.23 (−0.02, 0.49)	9 (23) [36,028]	Low	Downgraded for imprecision of 95% CI, once for inconsistency, and once for publication bias. Upgraded because we believe the largest study's intervention was not consistently implemented with fidelity.

*Note*: Positive standardized mean differences favor Montessori education over traditional education. Because some studies collected data longitudinally in both control and experimental groups and/or used multiple measures of an outcome, we report the total number of *observations* instead of the number of participants.

^a^ The standardized mean difference effect size reported here is Hedges' *g* using a cluster‐robust method (Tanner‐Smith, 2014; Tanner‐Smith, 2016; Tipton, 2015).

## SOURCES OF SUPPORT

- Wend Collective, USA

The Wend Collective provided partial support of this review through a grant to Angeline Lillard.

- Tift College of Education Seed Grant (2015–2016), USA

A Tift College of Education Seed Grant (Mercer University) provided $2500 to hire Graduate Research Assistants to assist with data collection and extraction (2015–2016).

- Tift College of Education Seed Grant (2016–2017), USA

A Tift College of Education Seed Grant (Mercer University) provided $2500 to hire Graduate Research Assistants to assist with data collection and extraction (2015–2016).

## DIFFERENCES BETWEEN PROTOCOL AND REVIEW

There were several differences between the protocol and review:

- We made some small changes to the introductory sections because another subject matter expert (Lillard) was added as a co‐author after the protocol had been published.
- We updated our overall procedures to comply with the latest version of the MECCIR conduct standards (Campbell Collaboration, 2019a) and MECCIR reporting standards (Campbell Collaboration, 2019b) and the second edition of the Cochrane Handbook for Systematic Reviews (Higgins, 2021).
- We created more specific inclusion and exclusion criteria to help define what qualifies as baseline equivalency.
- Studies excluded because there was insufficient information to compute the relevant effect size were not narratively described because of the large number of studies excluded for this reason. However, we have provided readers with access to a spreadsheet that shows reasons for exclusion and notes.
- We adopted the ROBINS‐I (Sterne, 2016) tool to assess risk of bias in nonrandomized studies.
- We included Benjamini‐Hochberg (Benjamini, 1995) corrected α values in an online supplement for those interested in null hypothesis statistical testing.
- Because almost all studies used continuous outcomes, we used a standardized mean difference effect size (specifically Hedges' *g*) as the sole effect size metric.
- We provided additional information about three different methods of effect size calculation that were used.
- Because multiple methods of effect‐size estimation were used, we conducted a meta‐regression sensitivity analysis to see the degree to which those estimation methods covaried with outcomes.
- For outcomes with more than 15 studies, we originally intended to deal with unit‐of‐analysis issues using multilevel meta‐analysis methods described in Viechtbauer (2010) and the methods in Konstantopoulos (2011) or the “pre‐defined hierarchy of outcomes” approach. However, because of the strict assumptions of multilevel approaches, we adopted cluster‐robust methods (Tanner‐Smith, 2014; Tanner‐Smith, 2016; Tipton, 2015) that have become more accessible since the protocol had been published.
- Similarly, we adopted cluster‐robust models instead of the proposed simple, random effects models for outcomes with 15 or fewer outcomes. The exception is the social studies, in which there was just one study with multiple effect sizes so a random‐effects model was used.
- We conducted a sensitivity analysis as suggested in Tanner‐Smith (2016) and Tipton (2015) to see how different assumptions about the correlation between within‐study effect sizes (*ρ*) might affect cluster‐robust results.
- Because leave‐one‐out sensitivity analyses have become easy to implement since the publication of the protocol, we conducted a leave‐one‐out sensitivity analysis.
- We used GOSH plots instead of forest plots when there were too many effect sizes to create a legible forest plot in R.
- We visually examined Bajaut plots, radial plots, and various types of residual and fit plots to examine study heterogeneity.
- An examination of funnel plots revealed an unexpectedly high degree of asymmetry with the bias in favor of Montessori education (i.e., studies were missing on the left/traditional education side of the funnel plots) on some outcomes. To make better decisions about the quality of evidence, we took the position of Duval (2000) that a trim‐and‐fill analysis would help us quantify the degree of publication bias while steering away from binary null hypothesis statistical significance tests of publication bias, as suggested by Higgins (2021). Although we did not intend to conduct a sophisticated analysis for publication bias, we think that the ability to quantify the effects of the potential publication bias on effect sizes warrants this deviation from the protocol.
- The protocol was somewhat unclear in the moderator/subgroup analyses that would be conducted, so we clarified them here.
- In the protocol, we proposed examining academic and behavioral outcomes. After using an emergent approach to identify specific outcomes, we determined that the term *nonacademic outcomes* is a more accurate descriptor than *behavioral outcomes*. We found that the variety of nonacademic outcomes measured in the Montessori literature was so broad, and not limited to just behavioral outcomes, that the best descriptor is simply *nonacademic*.

## Supporting information

Supporting information.

## Footnotes

1 As the current review was going to press, we became aware of the publication of another Montessori meta‐analysis, which, largely, had effect sizes similar to the ones presented here. See Demangeon, A., Claudel‐Valentin, S., Aubry, A., & Tazouti, Y. (2023). A Meta‐Analysis of the Effects of Montessori Education on Five Fields of Development and Learning in Preschool and School‐Age Children. Contemporary Educational Psychology, 102182. doi:10.1016/j.cedpsych.2023.102182

2 See the supplemental information (Randolph, 2021) for informaton on reasons for exclusion.
