# Supplementary material for: Montessori education's impact on academic and nonacademic outcomes: A systematic review
Source: Campbell Syst Rev. 2023 Aug 7;19(3):e1330. doi: 10.1002/cl2.1330 (PMC10406168; doi:10.1002/cl2.1330)
Supplement: Supplementary file 1 — Supporting information. [file CL2-19-e1330-s001.docx]

Appendices

1 Search Syntax by Database

| **Database** | Search Strings |
| --- | --- |
| Web of Science Arts & Humanities Citation Index (1975-current) | ab(montessori) AND (student* OR school* OR class*) AND ((achievement OR performance OR abilit*) OR (behavior* OR behaviour* OR social*)) AND (traditional OR conventional OR regular OR public OR mainstream OR standard) AND (compar* OR outcome*)  Limits: type=Dissertations and theses OR scholarly journals |
| EBSCO Academic Search Complete (1887-current) | montessori AND ( (student* OR school* OR class*)) AND ( (achievement OR performance OR abilit*) OR (behavior* OR behaviour* OR social*)) AND ( (traditional OR conventional OR regular OR public OR mainstream OR standard)) AND ( (compar* OR outcome*)) |
| EBSCO Education Full‐Text [Education Research Complete] (1881-current) | (montessori) AND ((“academic achievement*” OR “achievement rating*” OR “achievement test*” OR “academic abilit*” OR “educational indicator*” OR “academic outcome*” OR “knowledge level” OR “educational assessment*” OR “outcome* of education” OR “outcome measure*” OR “social indicator*” OR “summative evaluation*” OR “program evaluation*” OR “program effectiveness” OR “student evaluation*” OR test* OR effective*) |
| EBSCO PsycINFO (1887-current) | montessori AND ( student* OR school*) AND ( traditional OR conventional) AND ( (achievement OR performance OR abilit*) OR behavior* OR behaviour* OR social*) |
| EBSCO Professional Development Collection (1940-current) | montessori AND ( traditional OR conventional OR regular OR public OR mainstream OR standard) |
| EBSCO SocINDEX with Full Text (1881-current) | montessori AND ( traditional OR conventional OR regular OR public OR mainstream OR standard) |
| EBSCO Sociological Collection (1947-current) | montessori AND ( traditional OR conventional OR regular OR public OR mainstream OR standard) |
| EBSCO Teacher Reference Center (1984-present) | (montessori) AND ((“academic achievement*” OR “achievement rating*” OR “achievement test*” OR “academic abilit*” OR “educational indicator*” OR “academic outcome*” OR “knowledge level” OR “educational assessment*” OR “outcome* of education” OR “outcome measure*” OR “social indicator*” OR “summative evaluation*” OR “program evaluation*” OR “program effectiveness” OR “student evaluation*” OR test* OR effective*) |
| ERIC [EBSCO and web] (1966-current) | (montessori) AND ((“academic achievement*” OR “achievement rating*” OR “achievement test*” OR “academic abilit*” OR “educational indicator*” OR “academic outcome*” OR “knowledge level” OR “educational assessment*” OR “outcome* of education” OR “outcome measure*” OR “social indicator*” OR “summative evaluation*” OR “program evaluation*” OR “program effectiveness” OR “student evaluation*” OR test* OR effective*) |
| JSTOR (1800s-current) | (ab:(montessori)) AND disc:(education-discipline) |
| ProQuest Education Journals [ProQuest Education Database] (1991-current) | ab(montessori) AND (student* OR school* OR class*) AND ((achievement OR performance OR abilit*) OR (behavior* OR behaviour* OR social*)) AND (traditional OR conventional OR regular OR public OR mainstream OR standard) AND (compar* OR outcome*) |
| ProQuest Dissertations & Theses (1637-current) | ab((montessori AND (traditional OR conventional OR regular OR public OR mainstream OR standard)) |
| ProQuest Research Library (1971-current) | ab((montessori AND (traditional OR conventional OR regular OR public OR mainstream OR standard)) |
| ProQuest Social Sciences Journals [Social Science Database] (1994-current) | (montessori) AND (student* OR school* OR class*) AND ((achievement OR performance OR abilit*) OR (behavior* OR behaviour* OR social*)) AND (traditional OR conventional OR regular OR public OR mainstream OR standard) AND (compar* OR outcome*)  Limiters: Source type=Scholarly Journals OR Dissertations & Theses OR Conference Papers & Proceedings OR Other Sources OR Reports |
| Web of Science Social Sciences Citation Index (1900-current) | (montessori) AND ((“academic achievement*” OR “achievement rating*” OR “achievement test*” OR “academic abilit*” OR “educational indicator*” OR “academic outcome*” OR “knowledge level” OR “educational assessment*” OR “outcome* of education” OR “outcome measure*” OR “social indicator*” OR “summative evaluation*” OR “program evaluation*” OR “program effectiveness” OR “student evaluation*” OR test* OR effective*) |
| **Grey Literature Source** | **Search Strategy** |
| AERA Online Paper Repository (2010-current)  https://www.aera.net/Publications/Online-Paper-Repository/AERA-Online-Paper-Repository | Simple search: montessori |
| American Montessori Society Montessori Research Library (coverage undetermined)  https://amshq.org/Research/Research-Library | Simple search: traditional  Limiters: Topic=Outcome |
| Google Scholar (coverage undetermined)  https://scholar.google.com | Montessori AND comparison AND traditional |
| Open Grey [discontinued] (1980-2020)  http://www.opengrey.eu/ | Simple search: montessori |

## 2 Description of Risk of Bias Domains

| **Bias domain** | **Issues addressed*** |
| --- | --- |
| *Bias arising from the randomization process* | Whether:   - the allocation sequence was random; - the allocation sequence was adequately concealed; - baseline differences between intervention groups suggest a problem with the randomization process. |
| *Bias due to deviations from intended interventions* | Whether:   - participants were aware of their assigned intervention during the trial; - carers and people delivering the interventions were aware of participants’ assigned intervention during the trial.   *When the review authors’ interest is in the effect of assignment to intervention*   - (if applicable) deviations from the intended intervention arose because of the experimental context (i.e. do not reflect usual practice); and, if so, whether they were unbalanced between groups and likely to have affected the outcome; - an appropriate analysis was used to estimate the effect of assignment to intervention; and, if not, whether there was potential for a substantial impact on the result.   *When the review authors’ interest is in the effect of adhering to intervention*   - (if applicable) important non-protocol interventions were balanced across intervention groups; - (if applicable) failures in implementing the intervention could have affected the outcome; - (if applicable) study participants adhered to the assigned intervention regimen; - (if applicable) an appropriate analysis was used to estimate the effect of adhering to the intervention. |
| *Bias due to missing outcome data* | Whether:   - data for this outcome were available for all, or nearly all, participants randomized; - (if applicable) there was evidence that the result was not biased by missing outcome data; - (if applicable) missingness in the outcome was likely to depend on its true value (e.g. the proportions of missing outcome data, or reasons for missing outcome data, differ between intervention groups). |
| *Bias in measurement of the outcome* | Whether:   - the method of measuring the outcome was inappropriate; - measurement or ascertainment of the outcome could have differed between intervention groups; - outcome assessors were aware of the intervention received by study participants; - (if applicable) assessment of the outcome was likely to have been influenced by knowledge of intervention received. |
| *Bias in selection of the reported result* | Whether:   - the trial was analysed in accordance with a pre-specified plan that was finalized before unblinded outcome data were available for analysis; - the numerical result being assessed is likely to have been selected, on the basis of the results, from multiple outcome measurements within the outcome domain; - the numerical result being assessed is likely to have been selected, on the basis of the results, from multiple analyses of the data. |
| This table was reproduced from Higgins (2021) pp. 209-210. | |
